# Single-cell transcriptome analysis illuminating the characteristics of species-specific innate immune responses against viral infections

**DOI:** 10.1093/gigascience/giad086

**Published:** 2023-10-17

**Authors:** Hirofumi Aso, Jumpei Ito, Haruka Ozaki, Yukie Kashima, Yutaka Suzuki, Yoshio Koyanagi, Kei Sato

**Affiliations:** Division of Systems Virology, Department of Microbiology and Immunology, The Institute of Medical Science, The University of Tokyo, Tokyo 1088639, Japan; Institute for Life and Medical Sciences, Kyoto University, Kyoto 6068507, Japan; Graduate School of Pharmaceutical Sciences, Kyoto University, Kyoto 6068501, Japan; Department of AI Systems Medicine, M&D Data Science Center, Tokyo Medical and Dental University, Tokyo 1138510, Japan; Division of Systems Virology, Department of Microbiology and Immunology, The Institute of Medical Science, The University of Tokyo, Tokyo 1088639, Japan; Bioinformatics Laboratory, Faculty of Medicine, University of Tsukuba, Tsukuba 3050821, Japan; Center for Artificial Intelligence Research, University of Tsukuba, Tsukuba 3058577, Japan; Laboratory of Systems Genomics, Graduate School of Frontier Sciences, The University of Tokyo, Kashiwa 2778561, Japan; Laboratory of Systems Genomics, Graduate School of Frontier Sciences, The University of Tokyo, Kashiwa 2778561, Japan; Institute for Life and Medical Sciences, Kyoto University, Kyoto 6068507, Japan; Graduate School of Pharmaceutical Sciences, Kyoto University, Kyoto 6068501, Japan; Division of Systems Virology, Department of Microbiology and Immunology, The Institute of Medical Science, The University of Tokyo, Tokyo 1088639, Japan; International Research Center for Infectious Diseases, The Institute of Medical Science, The University of Tokyo, Tokyo 1088639, Japan; International Vaccine Design Center, The Institute of Medical Science, The University of Tokyo, Tokyo 1088639, Japan; Graduate School of Medicine, The University of Tokyo, Tokyo 1130033, Japan; Graduate School of Frontier Sciences, The University of Tokyo, Kashiwa 2778561, Japan; Collaboration Unit for Infection, Joint Research Center for Human Retrovirus infection, Kumamoto University, Kumamoto 8600811, Japan; CREST, Japan Science and Technology Agency, Kawaguchi 3320012, Japan

**Keywords:** innate immunity, mammal, virus infection, single-cell RNA sequencing, tensor

## Abstract

**Background:**

Bats harbor various viruses without severe symptoms and act as their natural reservoirs. The tolerance of bats against viral infections is assumed to originate from the uniqueness of their immune system. However, how immune responses vary between primates and bats remains unclear. Here, we characterized differences in the immune responses by peripheral blood mononuclear cells to various pathogenic stimuli between primates (humans, chimpanzees, and macaques) and bats (Egyptian fruit bats) using single-cell RNA sequencing.

**Results:**

We show that the induction patterns of key cytosolic DNA/RNA sensors and antiviral genes differed between primates and bats. A novel subset of monocytes induced by pathogenic stimuli specifically in bats was identified. Furthermore, bats robustly respond to DNA virus infection even though major DNA sensors are dampened in bats.

**Conclusions:**

Overall, our data suggest that immune responses are substantially different between primates and bats, presumably underlying the difference in viral pathogenicity among the mammalian species tested.

## Background

Although a virus can infect various animal species, the pathogenicity of the infection can differ among host species. For example, Old World monkeys, including rhesus macaques (*Macaca mulatta*), are naturally infected with Cercopithecine herpesvirus 1 (also known as B virus) without any observable disorders, while humans (*Homo sapiens*) exhibit severe disorders after infection [1]. Bat species are naturally infected with a variety of viruses and behave as natural reservoirs of human pathogenic viruses [2]. For example, Marburg virus infection causes severe symptoms in humans but not in Egyptian fruit bats (*Rousettus aegyptiacus*), a putative natural host of this virus [3]. One possible factor that could define the differences in viral pathogenicity among host species is the difference in innate immune responses. For example, a previous study reported that Egyptian fruit bats lack the induction of proinflammatory cytokines, including *CCL8, FAS*, and *IL6*, which are related to disease severity in humans, upon Marburg virus infection, suggesting that the lack of cytokine induction is one of the reasons why Egyptian fruit bats exhibit asymptomatic infection with Marburg virus [4].

Pathogen sensing is the initial step in triggering innate immune signaling. In a broad range of animals, including vertebrates, pathogen-associated molecular patterns (PAMPs) are recognized by pattern recognition receptors (PRRs) to induce subsequent immune responses [5–8]. In humans and mice (*Mus musculus*), double-stranded RNAs (dsRNAs), a PAMP for RNA viruses, are recognized by RNA sensors, such as RIG-I, MDA5, LGP2, TLR3, and TLR7/8 [5, 6]. Extrachromosomal DNAs, a PAMP for DNA viruses, are recognized by cytosolic DNA sensors (e.g., cGAS, AIM2, and IFI16) and endosomal DNA sensors (e.g., TLR9) [5, 6, 9]. Lipopolysaccharide (LPS), a PAMP for bacteria, is recognized by TLR4 [5, 6, 10]. Once PAMPs are recognized by PRRs, type I interferons (IFNs) are produced, leading to the induction of IFN-stimulated genes (ISGs), which include many antiviral genes [5, 6].

In contrast to the similarities in the immune system between humans and mice, the immune system of bats is assumed to be quite different from that of humans in various aspects [11–13]. Genome analysis of Egyptian fruit bats showed expansion and diversification of immune-related genes, including type I IFN genes [14]. Transcriptome analysis showed that type I IFNs in the Australian black flying fox (*Pteropus alecto*) are constitutively expressed in unstimulated tissues, leading to the constitutive expression of ISGs [15]. These observations suggest that immunity in bats may be stronger than that in other mammals. In contrast, some studies have proposed that immune responses in bats are dampened, resulting in bats exhibiting stronger tolerance to various viruses [12, 14, 16]. In particular, it is known that critical molecules involved in viral DNA sensing, such as cGAS, AIM2, and IFI16, are dampened or genetically lost in some bat species, including Egyptian fruit bats [16, 17]. These differences in innate immunity between humans and bats could be one of the reasons why viral pathogenicity differs between these 2 mammals.

Previous works have highlighted the uniqueness of the bat immune system using genomic analysis [14, 15, 17], transcriptome analysis [4, 18–20], and molecular biological experiments that reconstituted a part of the bat immune system in cell culture systems [16, 21, 22]. However, it remains unclear how and to what extent the innate immune response to pathogenic stimuli varies among mammals. Particularly, it is unclear how different innate immune responses are elicited by viral infections in different cell types in each mammal. Here, we used peripheral blood mononuclear cells (PBMCs) from 4 mammalian species including the abovementioned Egyptian fruit bats and 3 pathogenic stimuli and conducted single-cell RNA sequencing (scRNA-seq) analysis to elucidate the differences in innate immune responses against pathogenic stimuli.

## Results

### Experimental design

**Figure 1:**
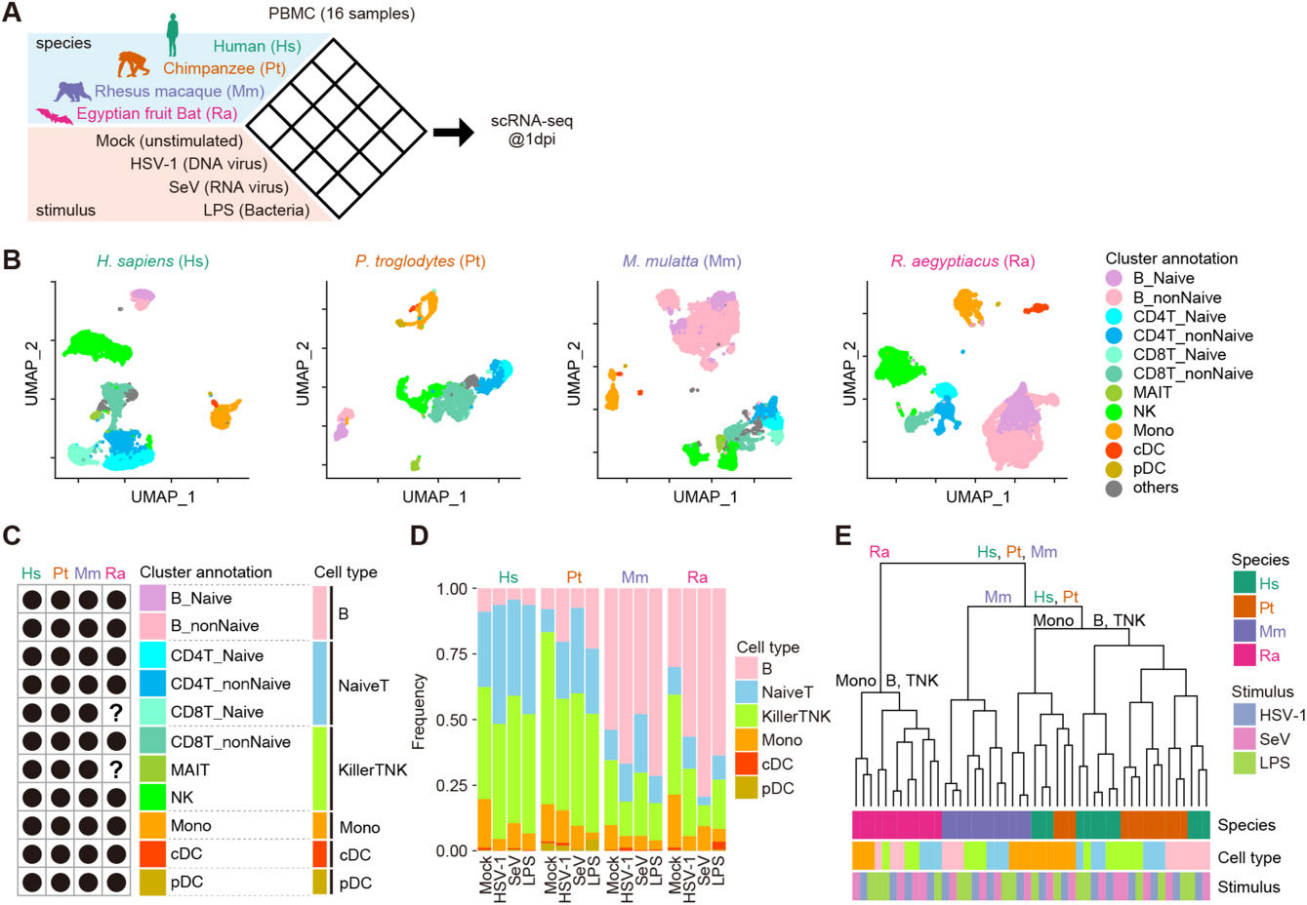
scRNA-seq analysis of PBMCs from 4 animal species inoculated with pathogenic stimuli. (A) Schematic of the experimental design. See also Supplementary Fig. S1. (B) Uniform manifold approximation and projection (UMAP) plots representing the gene expression patterns of the cells from the 4 species. Each dot is colored according to the cell type. Gray dots indicate cells unassigned into any cell type. See also Supplementary Fig. S2. (C) Comparison of identified cell types among the species. Dot: detected, question mark: undetected. The definitions of 6 species-common cell types are shown on the right side. See also Supplementary Fig. S2H. (D) The cellular compositions of PBMC samples. The compositions according to the 6 common cell types are shown. (E) Hierarchical clustering analysis of 48 pseudobulk datapoints (4 animal species × 3 stimuli × 4 cell types = 48 conditions) based on PC1-30 calculated from the fold-change values (respective stimulus versus unstimulated) for gene expression.

To illuminate the differences in immune responses to infectious pathogens among mammalian species, we isolated PBMCs from 4 mammals, including humans (*H. sapiens*, Hs), chimpanzees (*Pan troglodytes*, Pt), rhesus macaques (*M. mulatta*, Mm), and Egyptian fruit bats (*R. aegyptiacus*, Ra) (Fig. 1A). In this study, the Egyptian fruit bat was used as a representative model organism for bat species because this bat species is bred and available in captivity and is known to be a natural host of human pathogenic viruses, such as Marburg virus [3]. These PBMCs were inoculated with herpes simplex virus type 1 (HSV-1; a DNA virus), Sendai virus (SeV; an RNA virus), or LPS (a proxy for bacterial infection). We verified that these PBMCs could be infected with and/or respond to these viruses and LPS stimulation by quantifying viral RNAs and the upregulation of proinflammatory cytokines (e.g., IL1B and IL6), ISGs (e.g., EIF2AK2 and DDX58), and IFNB1 (Fig. S1A–C) at the level of messenger RNA (mRNA) transcripts.

To analyze immune responses to stimuli at single-cell resolution, we performed scRNA-seq analysis of 16 types of PBMC samples: 4 mammalian species (Hs, Pt, Mm, and Ra) versus 4 conditions (mock infection/stimulation, HSV-1 infection, SeV infection, and LPS stimulation) using the 10X Genomics Chromium platform at 1 day postinfection. Next, quality control (QC) was performed to exclude both cells with lower data quality and cells not targeted in this study (Supplementary Fig. S1D–G) (see Methods). Before QC, there was a group of cells with low genes per cell and counts per cell in PBMCs of SeV-infected bats (Supplementary Fig. S1D–E). Although 1 possible interpretation of this could be that SeV infection may have suppressed the gene expression in these cells, these cells were excluded to ensure the integrity of the downstream quantitative analysis. After filtering low-quality cells, a total of 40,717 cells from the 16 samples were used in the following analysis.

### The cellular composition of PBMCs from primates and bats

We characterized the cellular composition of PBMCs from each mammalian species by annotating the cell type of individual single cells using tools available in Seurat [23, 24] and Azimuth [25] (see Methods). To establish a common classification system for the cells from the different mammalian species, we first identified cell types present in multiple species (Fig. 1B, C). As cell types detected in multiple species, naive B cells, nonnaive B cells (including memory B cells and intermediate B cells), naive CD4^+^ T cells, nonnaive CD4^+^ T cells (including central memory CD4^+^ T cells, effector memory CD4^+^ T cells, proliferating CD4^+^ T cells, and regulatory T cells), naive CD8^+^ T cells, nonnaive CD8^+^ T cells (including central memory CD8^+^ T cells, effector memory CD8^+^ T cells, and proliferating CD8^+^ T cells), natural killer (NK) cells, mucosal-associated invariant T cells (MAITs), monocytes (Monos), conventional dendritic cells (cDCs), and plasmacytoid DCs (pDCs) were identified (Fig. 1C). Known marker genes for each cell type in humans were detected in the corresponding cell type in the unstimulated samples from the other animal species (Supplementary Fig. S2G). Although most cell types were detected in all 4 species investigated, naive CD8^+^ T cells and MAITs were undetectable in bat PBMCs, presumably because the cell numbers of these populations were relatively low in bats and/or the transcriptomic signatures of naive CD4^+^ T cells and nonnaive CD8^+^ T cells were too similar in bats (hereafter we simply referred to Egyptian fruit bats as “bats”) (Fig. 1C). This result was consistent with a previous study, in which clear clusters of naive CD8^+^ T cells and MAITs were not detected [26]. To establish a cellular classification system for the comparative transcriptome analysis, we defined 6 species-common cell types—namely, B cells, naive T cells, killer TNK cells, Monos, cDCs, and pDCs—according to similarities in expression patterns (Supplementary Fig. S2H).

The ratio of the 6 cell types exhibited different changes upon exposure to the stimuli in the different species (Fig. 1D). The frequency of monocytes decreased after stimulation in all 4 species, whereas the frequency of B cells and killer TNK cells changed differently within and across the animal species. Upon stimulation, there was generally a notable increase in B cells and a decrease of killer TNK cells in the bat (and nonhuman primates) samples, but not in the human samples.

### Immune response differs largely among animal species

To describe the differences in immune responses to various stimuli in specific cell types among animal species, we first calculated the average expression levels of appropriate genes in each condition (4 animal species × 4 stimuli × 6 cell types = 96 conditions). Using this “pseudobulk” transcriptome dataset, we first investigated which axis (i.e., animal species, stimulus, and cell type) was the most impactful element in shaping the expression patterns of immune cells. Thereby, we calculated the fold-change (FC) values of gene expression levels between unstimulated and corresponding stimulated conditions and performed principal component analysis (PCA) on the FC values. Hierarchical clustering analysis was subsequently performed according to principal components (PCs) 1–30. The transcriptome data branch according to the animal species and then branch according to the cell type followed finally by the stimulus (Fig. 1E). This suggests the difference in host species is the more impactful element in shaping the immune system, having a greater impact than the type of stimulus and cell type. In particular, bat PBMCs exhibited different transcriptomic patterns irrespective of the type of stimulus and cell type compared to the PBMCs from the other 3 species used. These results suggest that bats respond to pathogens in a different manner than primates.

### Extraction of species-specific immune responses

**Figure 2:**
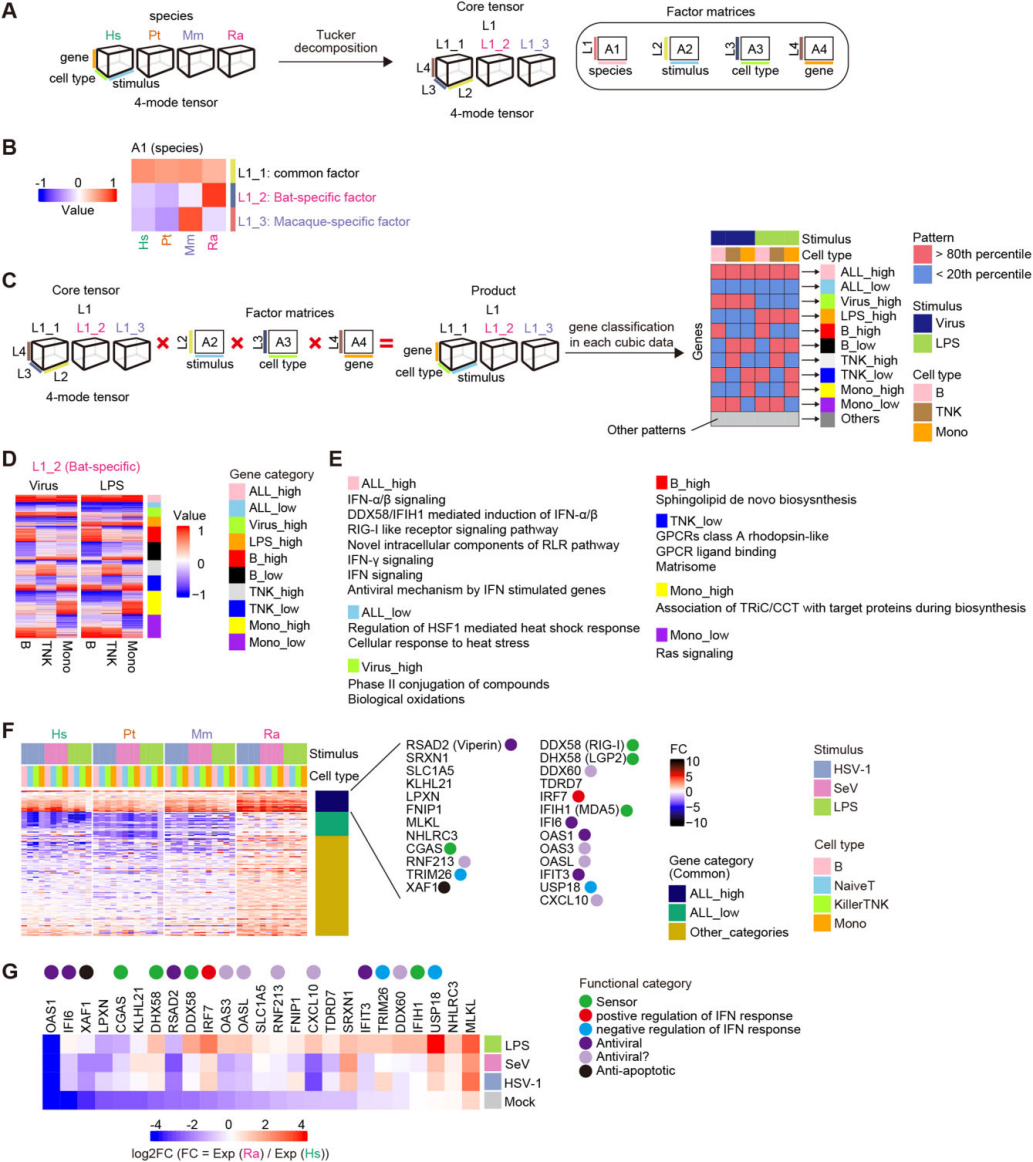
Characterization of species-specific immune responses using a tensor decomposition framework. (A) Tensor decomposition of the fold-change values for pseudobulk transcriptome data. (B) Heatmap representing a latent factor matrix relating to species. Columns indicate the animal species, and rows indicate the latent factors representing species-common (L1_1), bat-specific (L1_2), and macaque-specific (L1_3) factors. See also Supplementary Fig. S3A, B. (C) Classification of genes according to the differential patterns of the latent factors related to species. For each of the species-common (L1_1), bat-specific (L1_2), and macaque-specific (L1_3) factors, the product of the core tensor and 3 latent factor matrices related to stimulus, cell type, and gene was calculated (left), and the genes were classified into 11 categories according to the binary patterns for each calculated product (right). See also Supplementary Fig. S3C–F. (D) Heatmap representing the values of the products calculated in Fig. 2C. From the 3 products, the data related to the bat-specific factor (L1_2) are shown. Each row indicates the respective gene. The color keys shown on the right of the heatmap indicate gene categories. See also Supplementary Fig. S3G–L. (E) GO terms enriched in each gene category relating to the bat-specific factor. GO terms with a false discovery rate (FDR) ⇐ 0.1 and an odds ratio ≥1 are shown. (F) Heatmap representing the induction levels of ALL_high genes for the bat-specific factor. Additional classification according to the gene classification of the species-common factors is shown to the right of the heatmap. Genes categorized as ALL_high in both the species-common factor and the bat-specific factor are shown on the right side. The colored circle indicates the functional category of the gene. (G) Heatmap representing the relative expression levels (bats versus humans) of the genes shown in Fig. 2F.

We next characterized the differences in the immune responses to pathogenic stimuli among animal species. The FC values of our pseudobulk transcriptome dataset were represented by a 4-mode tensor (4 animal species × 3 stimuli × 6 cell types × 7,557 orthologous genes). To characterize this extraordinary high-dimensionality transcriptome dataset, we utilized Tucker decomposition, a method of tensor decomposition (Fig. 2A). In this analysis, we excluded cDC and pDC data due to many missing values. Tucker decomposition generated a core tensor and 4-factor matrices (A1–A4) related to the 4 axes (animal species, stimulus, cell type, and gene). For example, the factor matrix A1 (for host species) included 3 latent factors (L1_1, L1_2, and L1_3), which could be regarded to represent common, bat-specific, and macaque-specific expression patterns, respectively (Fig. 2B).

To characterize species-specific immune responses, we developed a gene classification system according to the pattern of the species-associated latent factor in the tensor decomposition framework. First, we calculated the product of a core tensor and the 3-factor matrices A2 (for stimulus), A3 (for cell type), and A4 (for gene) (Fig. 2C and Supplementary Fig. S3A, B). Consequently, we obtained 3 cubic datasets with 3 axes—stimulus, cell type, and gene. These cubic data were related to L1_1 (for the common factor), L1_2 (for the bat-specific factor), or L1_3 (for the macaque-specific factor). Subsequently, we classified the genes into 10 categories according to their expression patterns in each cubic dataset (the results for the bat-specific [L1_2] and other factors [L1_1 and L1_3] are shown in Fig. 2D, Supplementary Fig. S3G, and Supplementary Fig. S3I, respectively). In the factor matrix A2 (for stimulus), the values for the latent factors related to HSV-1 and SeV were similar (Supplementary Fig. S3A). Therefore, these 2 categories were integrated into the category “Virus” in the gene classification. Additionally, 2 cell type categories, NaiveT and KillerTNK, were integrated into the category “TNK” (Supplementary Fig. S3B). The pattern for raw FC values supported that the gene classification by the tensor decomposition framework succeeded in extracting the characteristic patterns of gene expression alterations upon pathogenic stimuli (Supplementary Fig. S3J–L).

### Differential dynamics of pathogen sensing and immune responses

To highlight the uniqueness of immunity in bats compared to that in primates, we focused on the expression pattern represented by the bat-specific factor (L1_2) and performed Gene Ontology (GO) analysis on the 10 gene categories (Fig. 2E). In the gene category “ALL_high,” which included genes upregulated particularly in bats regardless of the stimulus and cell type, GO terms related to innate immune responses, such as IFN signaling, DDX58/IFIH1-mediated induction of IFN, RIG-I–like receptor (RLR) signaling pathways, and the antiviral mechanism by ISGs, were overrepresented.

To dissect the “ALL_high” genes in the bat-specific factor, we further extracted the genes that belonged not only to the “ALL_high” category in the bat-specific factor but also to that in the common factor (L1_1). This fraction represented genes that were upregulated by stimuli in all species but whose induction levels were highest in bats. These genes included various PPRs, such as RLRs (RIG-I, LGP2, and MDA5) and cGAS, a DNA sensor, suggesting that these genes were upregulated to higher levels in bats than in the other species across the cell types and stimuli (Fig. 2F). There are 2 possible scenarios that could potentially explain these higher FC values observed in bats. One possibility is that expression levels of these genes after stimulation are higher than in primates. The second possibility is that basal expression levels of these genes in bats are lower than those in primates. Therefore, we calculated the relative expression levels of these genes in bats compared to humans and showed that the basal expression levels of these genes were lower in bats than in humans (Fig. 2G). These results suggest that the induction dynamics of these PRRs in bats are likely different from those in primates, possibly leading to the differences in the induction of immune responses.

### Robust immune responses to a DNA virus in bats

**Figure 3:**
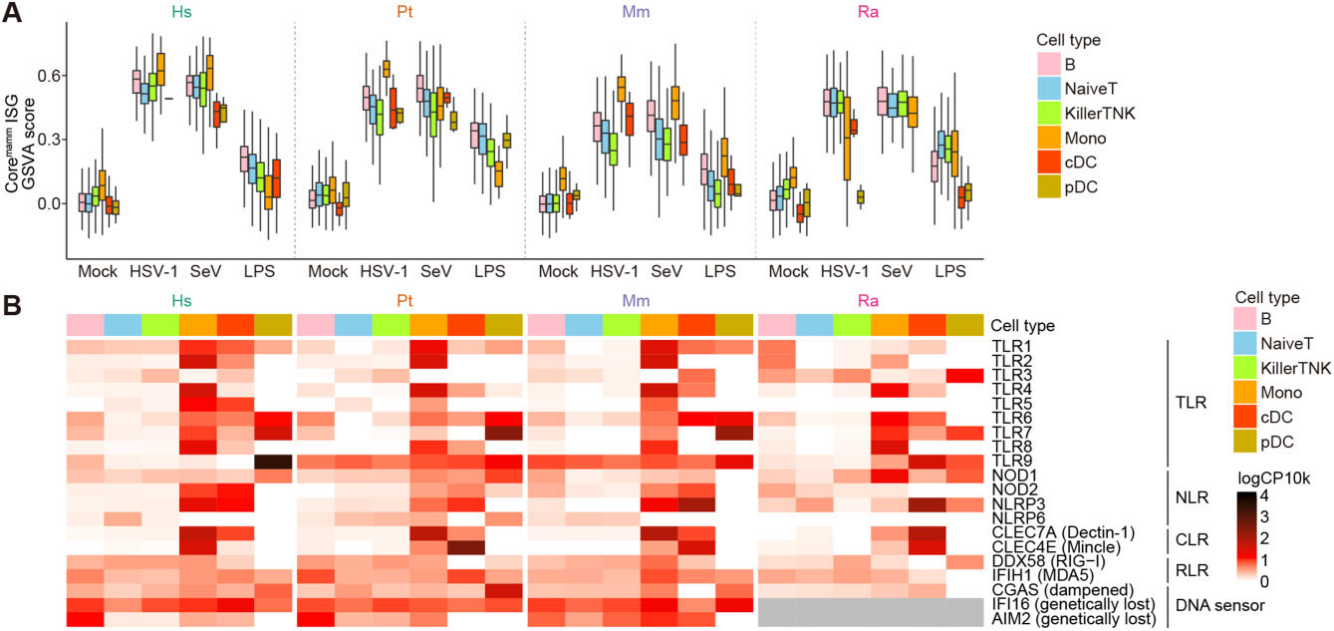
Robust immune responses to a DNA virus in bats. (A) Boxplot of the expression levels of core^mamm^ ISGs in every single cell. The y-axis indicates the global expression level (GSVA score) of the core^mamm^ ISGs. (B) Heatmap representing the mean expression levels of sensor genes. The mean values were calculated without using the information for the stimulus.

As critical DNA sensors, such as cGAS, AIM2, IFI16, and TLR9, are dampened or genetically lost in bat species [16, 17, 27], it has been hypothesized that bats, including Egyptian fruit bats, cannot efficiently activate innate immune responses against DNA viruses. To test this hypothesis, we analyzed the IFN response upon HSV-1 (a DNA virus) infection. However, the expression levels of IFN-α genes were not examined because they were not annotated in the transcript model for the Egyptian fruit bat used in this study, and the expression of IFN-β genes was too low. Thus, even though the expression level of IFN-I is the primary factor to examine the activity of the IFN response, we instead analyzed the induced levels of “core^mamm^ ISGs”—a set of genes that are commonly induced by type I IFNs across mammals that were defined in a previous study [28]. Intriguingly, we found that the core^mamm^ ISGs were upregulated upon HSV-1 infection in most cell types in bats (Fig. 3A). The induced levels were comparable to those induced by SeV (an RNA virus) infection and higher than those induced by LPS stimulation. Furthermore, the induced levels in bats were comparable to those in primates. This suggests that immune cells in bats can sense and respond to HSV-1 infection even though critical DNA sensors are dampened.

To address the possibility that pathogen sensors other than DNA sensors contribute to the sensing of HSV-1 infection in bats, we examined the expression levels of various PRRs (Fig. 3B). The expression of some PRRs, including TLR3, a dsRNA sensor associated with HSV-1 sensing in humans and mice [29], was detected not only in primates but also in bats, suggesting the possibility that these PRRs compensate in the response to HSV-1 infection in bats (see Discussion).

### Identification of bat-specific subsets of monocytes

**Figure 4:**
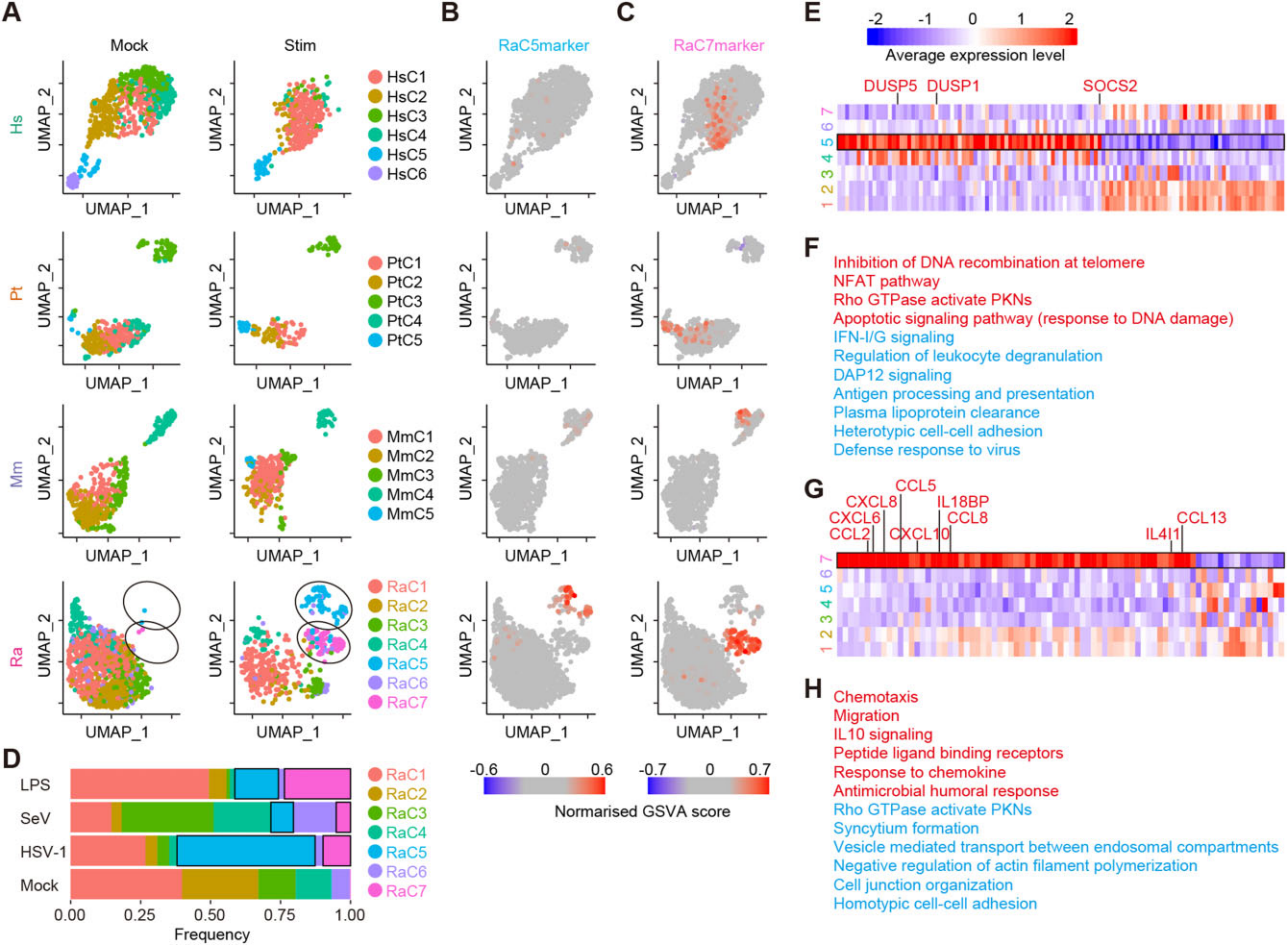
Identification of bat-specific subsets of monocytes. (A) UMAP plots representing the gene expression patterns of monocytes from the 4 species. The dots are colored according to the cell cluster defined for each animal species. See also Supplementary Fig. S4A. (B, C) UMAP plots representing the average expression levels of marker genes for cluster 5 [C5markers] (B) and cluster 7 [C7markers] (C). See also Supplementary Fig. S4B. (D) The cellular composition of bat monocytes. The composition is shown according to the cluster. The black frame indicates clusters 5 and 7 in stimulated samples. (E) Heatmap representing the mean expression levels of differentially expressed genes (DEGs) in cluster 5 of bat monocytes. (F) Summary of the GO terms enriched in DEGs in cluster 5. GO terms enriched in up- and downregulated genes are shown in red and blue, respectively. (G) Heatmap representing the mean expression levels of differentially expressed genes (DEGs) in cluster 7 of bat monocytes. (H) Summary of the GO terms enriched in DEGs in cluster 7. GO terms enriched in up- and downregulated genes are shown in red and blue, respectively.

Next, we investigated cellular subsets within the cell types that are characteristic in bats to explain the differences in immune responses among the species. We particularly searched for cellular subsets that specifically appeared after pathogenic stimulus exposure in each species according to the dimensionality reduction analysis of transcriptome data. In humans, chimpanzees, and macaques, no subset appeared in any cell type after stimulation (Supplementary Fig. S4A). Similarly, such subsets were not identified in T/NK or B cells in bats. In contrast, we found that 2 subsets of bat monocytes (referred to as clusters 5 and 7) specifically appeared after stimulation (Fig. 4A). To validate whether these subsets (clusters 5 and 7) are unique in bats, we identified marker genes for these clusters and subsequently examined whether the marker genes were expressed in monocytes from the other animal species. The marker genes for cluster 5 (referred to as C5 markers) were not highly expressed in any cluster of monocytes from primates (Fig. 4B). Furthermore, high expression levels of C5 markers in bat monocytes were found only after stimulation. This suggested that cluster 5 was not only bat specific but also specifically induced by pathogenic stimuli. Unlike the C5 markers, the marker genes for cluster 7 (C7 markers) were highly expressed not only in bat cluster 7 but also in some monocytes in primates (Fig. 4C). Although cells with higher expression of C7 markers were induced upon stimulation in both bats and primates, these cells in primates did not form a separate cluster similar to cluster 7 in bats (Supplementary Fig. S4B). Furthermore, the proportions of clusters 5 and 7 differed depending on the stimulus: HSV-1–infected and LPS-stimulated samples showed the highest frequencies of clusters 5 and 7, respectively (Fig. 4D).

To characterize these 2 clusters, we identified differentially expressed genes (DEGs) in clusters 5 and 7 compared to the other clusters of bat monocytes. According to GO analysis, cluster 5 is characterized by lower expression of ISGs (Fig. 4E, F). Additionally, cluster 5 highly expresses known suppressors of the inflammatory response, such as DUSP1, DUSP5, and SOCS2 [30–32]. On the other hand, cluster 7 can be characterized by a higher expression of various cytokines related to chemotaxis (Fig. 4G), including CXCL6, IL18BP, CXCL8, CCL2, CCL8, CCL13, CCL5, CXCL10, IL15, and IL4I1 (MSigDB [33]: GO:0060326) (Fig. 4G, H). Overall, we established that there are 2 unique subsets of bat monocytes with different characteristics (see Discussion).

## Discussion

Differences in viral pathogenicity among host species are thought to be attributed to differences in immune responses against viral infections among the species [34]. However, it remains unclear how immune responses, particularly innate immunity against viral infections, differ among host species. In the present study, we performed scRNA-seq on 16 types of PBMC samples, derived from a combination of 4 host species and 4 infection conditions (Fig. 1A), and showed that the differences in the immune responses among the host species were more impactful than those among both the stimuli and the cell types (Fig. 1E). In particular, the transcriptomic changes resulting from pathogenic stimulation in bats differed from those in primates. It is also noteworthy that poststimuli changes in the ratio of cell types differed between humans and bats (Fig. 1D). For further analysis, we established a bioinformatic pipeline to characterize species-specific immune responses from transcriptome profiles with extraordinarily high dimensions (4 animal species × 3 stimuli × 4 cell types × 7,557 orthologous genes) (Fig. 2A). We illuminate differences in innate immune systems among mammalian species that partly explain the differences in viral pathogenicity among host species.

It is known that 2 DNA-sensing pathways mediated by the cGAS-STING pathway [16] and PYHIN proteins, including AIM2 and IFI16 [17], are dampened in bats, including Egyptian fruit bats. In addition, a previous study using a cell line derived from big brown bats (*Eptesicus fuscus*) suggested that the TLR9-mediated DNA-sensing pathway is also weakened in bats [27]. Based on these observations, it was hypothesized that the ability to sense DNA virus infection is weakened in bats [12, 13]. However, we showed that bat PBMCs robustly induced IFN responses upon infection with the DNA virus HSV-1 (Fig. 3A). This suggests that bats can initiate an innate immune response after infection with DNA viruses (at least HSV-1) and that bats have another pathway to sense DNA viruses. An alternative possibility is that the IFN response in response to HSV-1 infection was triggered by sensing viral molecules other than DNAs. It is known that, in humans and mice, dsRNA sensing by TLR3 plays an important role in responding to HSV-1 infection [29, 35]. Additionally, the Egyptian fruit bat genome encodes an intact TLR3 gene (NCBI Gene ID: 107510436), and bat immune cells express TLR3 (Fig. 3B). Furthermore, other RNA sensors, such as RIG-I, LGP2, and MDA5, were upregulated in bat cells similarly as in primate cells upon HSV-1 infection (Fig. 3B). These data suggest that TLR3 or other RNA sensors in bats may compensate for weakened DNA-sensing pathways, leading to IFN responses to HSV-1 infection.

To characterize the bat-specific innate immune responses based on ultrahigh-dimensionality transcriptome data (4 animal species × 4 stimuli × 6 cell types × 7,557 orthologous genes), we established an analytical framework utilizing tensor deconvolution (Fig. 2A). This framework could (i) extract a species-specific effect on gene expression changes, (ii) compare the effects among the cell types and the stimuli, and (iii) classify genes according to the differential pattern of a species-specific effect among the cell types and the stimuli. Using this framework, we found that the expression levels of key DNA and RNA sensors, including cGAS, RIG-I, MDA5, and LGP2, were highly induced in bats compared with primates, regardless of the cell type or stimulus (Fig. 2F). Furthermore, the basal expression levels of these PRRs in bats were lower than those in humans (Fig. 2G). On the other hand, after stimulation, the expression levels of these PRRs in bats were comparable to those in humans. These results suggest that the induction dynamics of these PRRs in bats are likely different from those in primates, leading to the differences in the induction of immune responses. Indeed, several antiviral ISGs, such as IFI6 and IFIT3, exhibited expression dynamics similar to those of these PRRs (Fig. 2F, G). These differences could be one of the reasons why immune responses differ between bats and primates.

Another factor that can explain the differences in immune responses among host species is the presence of species-specific cellular subsets. In bat monocytes, we identified 2 subsets that were specifically induced by stimuli (i.e., clusters 5 and 7) (Fig. 4A). Cluster 5 was a bat-specific subset induced preferentially by HSV-1 infection (Fig. 4B, D). Interestingly, even though cluster 5 was induced after stimulation, cluster 5 exhibited lower expression of ISGs and higher expression of immunosuppressive genes (DUSP1, DUSP5, and SOCS2) [30–32] (Fig. 4E, F). This observation suggests that the immune responses in cluster 5 are downregulated presumably by negative feedback signaling and that cluster 5 may contribute to controlling excessive immune activation in bats. On the other hand, cluster 7 was identified as a monocyte subset that was mainly induced by LPS stimulation (Fig. 4C, D). Cluster 7 highly expressed several proinflammatory cytokines and chemokines (CXCL6, IL18BP, CXCL8, CCL2, CCL8, CCL13, CCL5, CXCL10, IL15, and IL4I1) (Fig. 4G, H). Cluster 7 may contribute to the recruitment of leukocytes since these cytokines are associated with the chemotaxis of neutrophils (CCL8, CXCL6, and CXCL8), basophils (CXCL8, CCL2, CCL5, CCL8, and CCL13), eosinophils (CCL5, CCL8, and CCL13), monocytes (CCL5, CCL8, and CCL13), T cells (CCL5, CCL8, CCL13, CXCL8, and CXCL10), and NK cells (CCL5 and CCL8) in humans and mice [36, 37]. Based on the expression pattern of the marker genes for cluster 7 (Fig. 4C and Supplementary Fig. S4B), cellular subsets corresponding to cluster 7 were also present in primate monocytes. However, these primate cells did not form a separate cluster in the dimensionality reduction analysis based on the transcriptome profile (Fig. 4A). These results suggest that the monocyte subset represented by cluster 7 exhibits unique gene expression and thus may exert unique functions in bats. Although the specific functions of these monocyte subsets (clusters 5 and 7) in immune responses in bats are still unclear, these unique subsets may contribute to bat-specific host immune responses.

### Limitations of the study

In the present study, we elucidated differences in innate immune responses among host species from various aspects. However, we did not address differences in the outcomes of the innate immune responses, such as differences in viral pathogenicity. Another limitation is that the bioinformatic resources we used, such as gene annotation, gene ontology, and cellular annotation, have been developed in a human-centric way. Therefore, there is the possibility that immune responses induced by species-specific genes and cell types were overlooked. Moreover, because the results of this study rely on an analysis using a single bat species, the Egyptian fruit bat, it is unclear whether the observed bat-specific characteristics are conserved across bat species. Furthermore, we did not perform biological replicates of scRNA-seq in this study. Despite these limitations, we present valuable resources to illuminate differences in immune responses among host species, including Egyptian fruit bats, and clues to elucidate differences in viral pathogenicity among species. Further study to elucidate the functional consequences of these differences is needed to reveal the mechanisms by which bats can tolerate infections with various viruses.

## Methods

### Cells

Vero cells (obtained from the Laboratory of Bernard Roizman, University of Chicago, USA).

LLC-MK2 cells (rhesus macaque kidney epithelial cells) (CCL-7, ATCC).

### PBMC collection

Human peripheral blood was obtained from the arm vein. To obtain chimpanzee peripheral blood, a chimpanzee was anesthetized for a regular health examination. Anesthesia was induced with intramuscular administration of the combination of 0.012 mg/kg medetomidine (Meiji Seika Pharma Co., Ltd.), 0.12 mg/kg midazolam (Sand Co., Ltd.), and 3.5 mg/kg ketamine (Fujita Pharm) and maintained with constant rate infusion (4–10 mg/kg/h) of propofol (1% Diprivan;, Sand Co., Ltd.). Peripheral blood was obtained from the femoral vein. To obtain rhesus macaque peripheral blood, a rhesus macaque was anesthetized. Anesthesia was induced with intramuscular administration of 8 mg/kg ketamine followed by deep anesthetization using an intravenous injection of sodium pentobarbital (30 mg/kg) (Kyoritsu Seiyaku). Peripheral blood was obtained by cardiac puncture before exsanguination and perfusion. Bat peripheral blood was obtained from the cephalic vein in the patagium. PBMCs were isolated from peripheral blood by density gradient centrifugation using Ficoll-Paque Plus (Cytiva, cat. 17144003).

### HSV-1 preparation and titration

HSV-1 (strain F; GenBank accession number: GU734771) [38] was prepared as previously described [29] and kindly provided by Dr. Yasushi Kawaguchi (The Institute of Medical Science, The University of Tokyo, Japan). Briefly, Vero cells were infected with HSV-1 and the supernatant was collected and used without purification. To titrate viral infectivity, prepared virus was diluted 10-fold in Medium 199 (Thermo Fisher Scientific, cat. 11825015) containing 1% fetal calf serum (FCS) (Nichirei Biosciences, cat. 175012), and Vero cells were infected with dilutions of the virus at 37°C. At 1 hour postinfection, the culture medium was replaced with Medium 199 containing 160 μg/mL human γ-globulin (Sigma Aldrich, G4386-25 G), and the cells were cultured at 37°C for 2 to 3 days. To calculate the viral titer (plaque-forming unit [PFU]), the number of plaques per well was counted.

### SeV preparation and titration

SeV (strain Cantrell, clone cCdi; GenBank accession number: AB855654) was prepared as previously described [39] and kindly provided by Dr. Takashi Irie (Hiroshima University, Japan). Briefly, LLC-MK2 cells were infected with SeV and the supernatant was collected and used without purification. To titrate viral infectivity, prepared virus was diluted 10-fold in Dulbecco’s modified Eagle’s medium (DMEM) (Sigma-Aldrich, cat. D6046-500ML) containing 10% FCS, and LLC-MK2 cells were infected with dilutions of the virus at 37°C. At 1 hour postinfection, the cells were washed with phosphate-buffered saline (PBS) and cultured with DMEM containing 10% FCS at 37°C. At 1 day postinfection, the infected cells were fixed with acetone (Nacalai Tesque, cat. 21914–03)/methanol (Nacalai Tesque, cat. 00310–95). To calculate the viral titer (cell infectious unit [CIU]), the fixed cells were stained with a rabbit anti-SeV polyclonal antibody [40] as the primary antibody and an Alexa 488–conjugated goat anti-rabbit IgG antibody (Thermo Fisher Scientific, cat. A-11008) as the secondary antibody, and the number of fluorescent foci per well was counted.

### Infection and stimulation

One million PBMCs were maintained in 500 μL RPMI 1640 medium (Sigma-Aldrich, cat. R8758-500ML) and infected with HSV-1 or SeV at a multiplicity of infection of 0.1. To mimic microbial infection, LPS (Sigma-Aldrich, cat. L5024-10MG) was added at a final concentration of 200 ng/mL. At 1 day postinfection, all types of infected/stimulated PBMCs were centrifuged, resuspended in PBS, and used for bulk quantitative reverse transcription polymeraes chain reaction (RT-qPCR) and scRNA-seq (see below).

### RT-qPCR

RT-qPCR was performed as previously described [41]. Briefly, cellular RNA was extracted using the QIAamp RNA Blood Mini Kit (Qiagen, cat. 52304) and then treated with an RNase-free DNase set (Qiagen, cat. 79254). Complementary DNA (cDNA) was synthesized using SuperScript III reverse transcriptase (Thermo Fisher Scientific, cat. 18080044) and random primers (Thermo Fisher Scientific, cat. 48190011). RT-qPCR was performed using Power SYBR Green PCR Master Mix (Thermo Fisher Scientific, cat. 4367659) and the primers listed in Supplementary Table S1. For RT-qPCR, the CFX Connect Real-Time PCR Detection System (Bio-Rad) was used.

### Sequencing of scRNA-seq libraries

scRNA-seq libraries were constructed using the Chromium Next GEM Single Cell 3′ Kit according to the manufacturer’s instructions (10X Genomics). Briefly, cells, gel beads, and oil were loaded onto the Chromium platform to generate single-cell gel beads-in-emulsion (GEMs). Before loading, cell numbers and viability were confirmed. To acquire 5,000 cells recovery, 8,000 cells were loaded. Barcoded cDNAs were pooled for amplification, and adaptors and indices for sequencing were added. The evaluation was conducted using a BioAnalyzer (Agilent Technologies). The libraries were sequenced with paired-end reads using the Illumina NovaSeq6000 platform (RRID:SCR_016387).

### Genome sequence dataset

Genome sequences of the animal species, including humans (GRCh38.p13, RefSeq accession: GCF_000001405.39), chimpanzees (Clint_PTRv2, RefSeq accession: GCF_002880755.1), rhesus macaques (Mmul_10, RefSeq accession: GCF_003339765.1), and Egyptian fruit bats (mRouAeg1.p, RefSeq accession: GCF_014176215.1), were obtained from NCBI RefSeq [42]. From the genome sequences, ALT contig sequences were excluded. The genome sequences of viruses including HSV-1 (strain: F, accession: GU734771.1) and SeV (strain: Cantell clone cCdi, accession: AB855654.1) were also obtained from NCBI RefSeq. A custom reference genome sequence for each animal species was generated by adding the genome sequences of HSV-1 and SeV to the genome sequence of the animal species.

### Gene annotation and ortholog information

Gene annotations of humans (GRCh38.p13, Release 109.20200228), chimpanzees (Clint_PTRv2, Release 105), rhesus macaques (Mmul_10, Release 103), and Egyptian fruit bats (mRouAeg1.p, Release 101) were obtained from NCBI RefSeq. From the gene annotations, only the records for protein_coding, transcribed_pseudogene, lncRNA, pseudogene, antisense_RNA, ncRNA_pseudogene, V_segment, V_segment_pseudogene, C_region, C_region_pseudogene, J_segment, J_segment_pseudogene, and D_segment were extracted according to the CellRanger tutorial [43]. In addition, to quantify viral RNA abundance, the records for viruses were added. The whole viral genome was treated as a single exon, and a total of 4 lines (the positive and negative strands of HSV-1 and SeV) were added.

A list of orthologous genes between humans and the other animal species (chimpanzees, rhesus macaques, and Egyptian fruit bats) was obtained from NCBI on 26 July 2021 [44]. From the file, the records for orthologs between humans (taxonomy ID: 9606) and chimpanzees (taxonomy ID: 9598), rhesus macaques (taxonomy ID: 9544), or Egyptian fruit bats (taxonomy ID: 9407) were extracted.

The ortholog list from NCBI lacked information on some critical immune-related genes of Egyptian fruit bats, such as CD4 and IRF1. Therefore, we retrieved information from the Bat1K gene annotation [45, 46] downloaded from the UCSC genome browser [47]: first, we made a custom gene annotation for Egyptian fruit bats by adding information from the Bat1K gene annotation to the RefSeq gene annotation. Second, we extracted exons in the Bat1K gene annotation that overlapped with exons in the RefSeq gene annotation by using the bedtools intersect command with the wao option (v2.30.0) [48]. In this step, the exons in the Bat1K gene annotation that did not overlap with the exons in the RefSeq gene annotation were also extracted and added to custom gene annotations as additional genes. Next, the exons that contained overlaps and had the same gene name (the same symbol or known to be an ortholog) were added to custom gene annotations as an alternative splicing variant of the gene. Then, the remaining overlapping exons were processed by determining which information (RefSeq or Bat1K) should be used preferentially. The criteria were as follows: (i) genes whose symbols are not prefixed with “LOC” were given priority, (ii) genes whose symbols are included in the human gene list were given priority, and (iii) information from RefSeq was given priority otherwise. According to these criteria, the annotation with the higher priority (RefSeq or Bat1K) was selected and used in the custom gene annotation.

As a result of the integration of gene annotations, the number of orthologous genes in the custom gene annotation of bats increased from 16,374 to 16,903. Importantly, immune-related genes that were not defined in the RefSeq gene annotation, such as TLR1, IRF1, and CD4, were added to the custom gene annotation.

Considering the orthologous relationships, we prepared 3 types of gene sets for each animal species: (i) “all genes,” including all genes in the animal species; (ii) “genes shared with humans,” including genes with orthologs in humans; and (iii) “common genes,” genes shared among the 4 analyzed animal species. Unless otherwise noted, “all genes” were used up to cell annotation, and “common genes” were used after cell annotation.

### Processing scRNA-seq data for generating count matrices

Gene expression count matrices for scRNA-seq data were generated using CellRanger (RRID:SCR_023221) (v6.0.1) (10X Genomics) [49, 50]. First, we built a custom reference for each animal species from the custom reference genome sequence and custom gene annotation using the “cellranger mkref” command. Subsequently, we generated unique molecular identifier (UMI)–based count matrices from the raw scRNA-seq data and custom references using the “cellranger count” command with default settings.

### QC of scRNA-seq data

First, we removed cells with abnormal genes per cell (genes/cell) and counts per cell (counts/cell) values using the Seurat package (RRID:SCR_016341) (v4.0.4) [23, 24]: cells with 800 to 5,000 genes/cell or 1,200 to 25,000 counts/cell were extracted. The thresholds were determined based on the distributions of genes/cell and counts/cell before QC (Supplementary Fig. S1D, E). Second, we annotated the cell type of individual cells using Azimuth (v0.4.3) [25], a reference-based cell annotation prediction program, and then, cells annotated as erythrocytes, platelets, hematopoietic stem cells, or innate lymphoid cells were excluded as nontargeted cells in the present study. This is because erythrocytes and platelets are probably residuals after experimental PBMC extraction, and hematopoietic stem cells and innate lymphoid cells are not the major cell types in the analysis of innate immune responses using PBMCs. In this step, the gene annotation “genes shared with humans” (see Gene annotation and ortholog information) for each animal species was used. Finally, regarding genes/cell and counts/cell values, cells with >3 |*z* score| were excluded as outliers.

### Data integration, visualization, and cell clustering

Data integration, visualization, and cell clustering for each animal species were performed using the Seurat package. In these processes, the expression levels of HSV-1 and SeV were not used.

Data integration is a method merging the gene expression count matrices obtained from different experimental conditions while removing batch effects. We integrated the count matrices from the 4 different conditions for each animal species. In the data integration, SCTransform (RRID:SCR_022146) (a modeling framework for the normalization and variance stabilization of molecular count data from scRNA-seq data) was performed using the SCTransform function for each count matrix. Next, to extract 2,000 genes with higher variance and thus greater information for integration, the 4 count matrices were processed using the SelectIntegrationFeatures function. Next, we used the PrepSCTIntegration function to transform normalized counts into counts per 10,000 counts in the cell (CP10k). After that, we used the FindIntegrationAnchors function with the setting Mock as a reference to find “Integration anchors.” Finally, we integrated the 4 normalized count matrices using the IntegrateData function with the option normalization.method=“SCT”.

For visualization, we first performed PCA using the RunPCA function. Then, UMAP (RRID:SCR_018217) [51] was performed with the RunUMAP function. In this step, PCs 1 to 50 were used, and the parameter “n.neighbors” was set individually for each animal species (Hs: 20, Pt: 20, Mm: 50, and Ra: 40).

To define cell clusters in each animal species, we performed graph-based unsupervised clustering (Supplementary Fig. S2A). First, the FindNeighbors function was used, and then, the FindClusters function was used. In these steps, the parameter “k.param” for FindNeighbors was set individually for each animal species (Hs: 12, Pt: 10, Mm: 10, and Ra: 20). The parameter “resolution” for FindClusters was also set individually for each animal species (Hs: 2.0, Pt: 2.2, Mm: 1.7, Ra: 1.2).

### Cell annotation

Regarding each cluster identified by graph-based unsupervised clustering in the section “Data integration, visualization, and cell clustering” (Supplementary Fig. S2A), 11 cell types were manually annotated according to (i) the predicted cell type by Azimuth (Supplementary Fig. S2B), (ii) the distances between each cluster (Supplementary Fig. S2C), and (iii) the correspondence of clusters between animal species (Supplementary Fig. S2D–F). First, reference-based cell type prediction was performed using Azimuth for the mock data from each animal species (Supplementary Fig. S2B). In this step, the gene annotation “genes shared with humans” (see Gene annotation and ortholog information) for each animal species was used. We checked the enrichment of each predicted cell type in each cluster by Azimuth. Second, we checked the similarities between clusters by hierarchical clustering (Supplementary Fig. S2C) using the mean values of PCs 1 to 50 among the individual cells (see Data integration, visualization, and cell clustering) in each cluster. Notably, PCA was performed using the expression levels of “all genes” (see Gene annotation and ortholog information). The Euclidean distance was used for clustering by Ward’s method. Third, to check the correspondence between clusters in each animal species, we performed data integration, clustering, and visualization for mock data from all 4 animal species (Supplementary Fig. S2D–F). In the integration, the mock data from humans were used as reference data. In this step, the gene annotation “common genes” (see Gene annotation and ortholog information) was used.

After categorizing cells into 11 cell types, the 11 cell types were coarse-grained into 6 cell types based on the results of hierarchical clustering analysis (see Hierarchical clustering). The 6 cell types were used in the subsequent analysis.

### Hierarchical clustering

To examine the similarities in expression patterns among the conditions (4 animal species × 4 stimuli × 6 cell types = 96 conditions), hierarchical clustering analysis was performed. In this analysis, the 5,000 genes with the highest median absolute deviation (mad) values were used (Supplementary Fig. S2H). First, the average expression levels of the respective genes in each condition were calculated. Next, PCA was performed using the average expression profiles. Third, using PCs 1 to 30, the distance matrix for the 96 conditions was generated using 1 − Pearson’s correlation coefficient. Finally, hierarchical clustering by Ward’s method was performed using the distance matrix.

To determine which factor (e.g., animal species, stimulus, or cell type) was the most impactful on the gene expression in immune cells, hierarchical clustering was performed using induction patterns upon stimulation (Fig. 1E). Unlike for the results shown in Supplementary Fig. S2H, FC values were used to perform PCA. This analysis used 7,557 genes, the union of the top 6,000 genes related to total expression levels in the expression profiles of each animal species. The FC expression values (stimulated vs. unstimulated conditions) of those genes were calculated for each cell type in each animal species. To avoid generating infinite FC values, the data for genes with zero expression in mock data were set at the minimum nonzero expression level in the mock data. Finally, hierarchical clustering was performed using the method described above.

### Tensor decomposition

To extract species-specific/common induction patterns upon stimulation from transcriptome data with complex structures (4 animal species × 3 stimuli × 4 cell types × 7,557 orthologous genes), we used tensor decomposition (Fig. 2A). As the input data for tensor decomposition, the FC values of 7,557 genes, the union of the top 6,000 genes related to total expression levels in the expression profiles of each animal, were used. The calculation method for FC values is described in the section “Hierarchical clustering.” The standardized FC values for each condition were represented as a 4-mode tensor (animal species × stimulus × cell type × orthologous gene). To perform Tucker decomposition (TD), a method of tensor decomposition, we used TensorLy (v0.6.0) [52]. We performed TD via higher-order orthogonal iteration (HOI) with the parameter init=“svd”. In HOI, the size of the core tensor (ranks) was set as [animal species: 3, stimulus: 2, cell type: 3, gene: 15]. The number of iterations was set as 100.

### Gene classification using the tensor decomposition results

A schematic of the gene classification using tensor decomposition is shown in Fig. 2C and Supplementary Fig. S3C–F. Briefly, we selected the candidate gene categories that had patterns of values (high, mid, or low) (Supplementary Fig. S3C) that matched the ideal pattern (Supplementary Fig. S3D) and then selected the gene category with the best “similarity score” (Supplementary Fig. S3E) from the candidates as the gene category for that gene (Supplementary Fig. S3F).

Initially, the product of the core tensor and the 3 factor-matrices, A2 (for stimulus), A3 (for cell type), and A4 (for gene), was calculated to obtain 3 cubic data with 3 axes, stimulus, cell type, and gene, using the ttl function of rTensor (v1.4.8) [53]. Each cubic data point indicated information related to species-common, bat-specific, and macaque-specific factors (Fig. 2B). Next, since the values of latent factors related to HSV-1 and SeV were similar (Supplementary Fig. S3A), these 2 categories were integrated into the category “Virus” by calculating mean values. Additionally, since the values of latent factors related to NaiveT and KillerTNK were similar (Supplementary Fig. S3B), these 2 categories of cell types were integrated into the category “TNK” by calculating mean values. Thus, hereafter, the category of stimuli included virus and LPS, and the category of cell types included B cells, TNK cells, and Monos.

Then, in each cubic data, genes were classified into 11 categories (Fig. 2C) through the following 3 steps. Briefly, from the candidate gene categories that had patterns of values (high, mid, or low) (Supplementary Fig. S3C) that matched the ideal pattern (Supplementary Fig. S3D), the gene category with the lowest “similarity score” (Supplementary Fig. S3E) was selected as the gene category for that gene (Supplementary Fig. S3F).

In the first step (Supplementary Fig. S3C), the values in each cubic data were normalized, and the genes were classified into 3 classes (high, mid, and low) according to the ranking of values in each condition (stimulus × cell type). First, 6 column vectors in the TD results for the 6 conditions (2 stimuli × 3 cell types) were normalized by dividing them by the 90th percentile for the individual vectors. After the division step, to suppress the effect of abnormally high or low values, data with >1 or <−1 were assigned as 1 and −1, respectively. Next, the genes were categorized into 3 classes based on the rule that if the rank of a value was greater than the 80th percentile or smaller than the 20th percentile, it was categorized as “high” or “low,” respectively; otherwise, it was categorized as “mid.”

In the second step (Supplementary Fig. S3E), a “similarity score” was calculated to represent the similarity between the genewise pattern of the TD results and the “ideal patterns” for each gene category. The “ideal patterns” were defined as vectors composed of 1, 0, and −1 for 16 gene categories (Virus_high, LPS_low, Virus_low, LPS_high, B_high, TNKM_low, B_low, TNKM_high, TNK_high, BM_low, TNK_low, BM_high, M_high, BTNK_low, M_low, and BTNK_high) (Supplementary Fig. S3D). The “similarity score” was defined as the sum of the residual squares between the 2 vectors, the genewise vector of normalized values from the TD results (Supplementary Fig. S3C) and the “ideal patterns” (Supplementary Fig. S3D). According to the definition, the “similarity scores” for every combination of genes and gene categories were calculated. After calculating all similarity scores, to obtain the threshold for checking if a gene should be recognized as a gene in that category, the 20th percentile of the similarity score in the vector for each gene category was calculated.

In the third step (Supplementary Fig. S3F), the gene category for each gene was determined. First, the candidate gene categories for each gene were filtered according to the pattern assigned in the first step (Supplementary Fig. S3C). If the pattern (high/mid/low) of all 6 conditions was high or low, the gene was categorized as ALL_high or ALL_low, respectively. If the pattern of a gene matched the “ideal pattern” of a gene category, the gene category was added as a candidate gene category for the gene. For example, if the pattern of gene A was (Virus_B: high, Virus_TNK: high, Virus_M: high, LPS_B: high, LPS_TNK: low, LPS_M: mid), the candidate gene category for gene A was “Virus_high” and “B_high” because all virus-infected data were assigned as “high” and all B-cell data were assigned as “high” (Supplementary Fig. S3D). Second, the gene category with the lowest “similarity score” among the candidate gene categories was selected as the tentative gene category. In this selection, if the “similarity score” was higher than the threshold of the gene category (Supplementary Fig. S3E), the gene was categorized as “Others” (see gene B in Supplementary Fig. S3F) because the pattern for the gene was recognized as being too different from the “ideal pattern.” If no candidate gene category was available, the gene was also classified as “Others” (see gene C in Supplementary Fig. S3F). Finally, the final gene category was determined by integrating similar gene categories (Supplementary Fig. S3F). For instance, the categories Virus_high and LPS_low were integrated into the category Virus_high because both categories indicated that virus-infected data were higher than LPS-stimulated data (see gene D in Supplementary Fig. S3F). As a result of the gene classification process, genes were categorized into one of 11 categories (Fig. 2C and Supplementary Fig. S3D).

### GO term enrichment analysis

GO analysis was performed with Fisher’s exact test. This analysis used the GO canonical pathways and GO biological processes defined by MSigDB (RRID:SCR_022870) (v7.3) [30]. Adjusted *P* values were calculated using the Benjamini‒Hochberg (BH) method.

### Calculation of gene set variation analysis (GSVA) scores

The gene set-wise expression scores used in Fig. 3A, Fig. 4B, C, and Supplementary Fig. S4B were calculated using GSVA (RRID:SCR_021058) (v1.38.2) [54, 55] with the algorithm “ssgsea.”

### Identification of DEGs and marker genes

In bat monocytes, DEGs were identified in cluster 5 or cluster 7 compared to the other clusters using the FindMarkers function of Seurat packages. A gene that met the following 3 criteria was considered a DEG: (i) the false discovery rate (FDR) calculated using the BH method was less than 0.05, (ii) the average log2FC was greater than 1 or less than −1, and (iii) the proportion of expressing cells was greater than 0.2.

The marker genes of cluster 5 and cluster 7 of bat monocytes (RaC5marker and RaC7marker, respectively) were defined as upregulated DEGs in cluster 5 (Fig. 4E) and cluster 7 (Fig. 4G), respectively.

## Availability of Source Code and Requirements

Project name: scRNA-seq_PBMC_Animals_Aso_et_al

Project homepage: https://github.com/TheSatoLab/scRNA-seq_PBMC_Animals_Aso_et_al [56]

Operating system: Linux

Programming languages: bash, R, Python

License: CC0-1.0

## Supplementary Material

giad086_GIGA-D-23-00007_Original_Submission

giad086_GIGA-D-23-00007_Revision_1

giad086_Response_to_Reviewer_Comments_Original_Submission

giad086_Reviewer_1_Report_Original_Submission Urs Greber -- 3/14/2023 Reviewed

giad086_Reviewer_1_Report_Revision_1 Urs Greber -- 8/24/2023 Reviewed

giad086_Reviewer_2_Report_Original_Submission Doreen Ikhuva Lugano -- 5/23/2023 Reviewed

giad086_Supplemental_Files

## Data Availability

The raw and processed single-cell RNA-seq data have been deposited in the Gene Expression Omnibus (GEO) database (GSE218199) and are publicly available. Original data to describe figures in this article have been deposited at Mendeley [58] and are publicly available. All additional supporting data are available in the *GigaScience* database [59].

## Additional Files

**Supplementary Fig. S1**. Validation of viral infectivity and the innate immune response (related to Fig. 1). (A) Heatmap of the induction levels of genes related to the IFN response and inflammation. The rows indicate genes, and the columns indicate combinations of species, stimulus, and dose. The color represents the log2 fold change of ddCt upon stimulation measured by qRT-PCR. “rep. 1” and “rep. 2” indicate biological replicates. (B, C) Heatmap of the expression levels of viral genes (B: HSV-1; C: SeV) measured by qRT-PCR. The rows indicate viral genes, and the columns indicate combinations of species and doses. “rep. 1” and “rep. 2” indicate biological replicates. The color represents the ddCt values based on the expression levels of GAPDH. (D–G) Violin plots of (D) the numbers of detected genes per cell before QC, (E) numbers of counted reads per cell before QC, (F) numbers of detected genes per cell after QC, and (G) numbers of counted reads per cell after QC.

**Supplementary Fig. S2**. Heterogeneous expression patterns in the 4 animal species (related to Fig. 1). (A, B) UMAP plots representing the gene expression patterns of PBMCs from the 4 species. Each dot is colored according to the results of unsupervised clustering (A) and reference-based label transfer (B). (C) Heatmaps showing pairwise Euclid distances representing the gene expression differences among clusters. The distances were calculated using PCs 1 to 50 of the gene expression data. (D, E) UMAP plots representing the gene expression patterns of PBMCs from the mock samples for the 4 species. Each dot is colored according to the results of unsupervised clustering using the integrated data for the 4 mock samples (D) or the 4 samples from each animal shown in Supplementary Fig. S2A (E). (F) Heatmaps showing pairwise Euclid distances representing the gene expression differences among clusters shown in Supplementary Fig. S2D. The distances were calculated using PCs 1 to 30 of the gene expression data. (G) Dot plots representing the expression patterns of marker genes for each cell type defined by Azimuth [57]. (H) Hierarchical clustering analysis of 48 pseudobulked FC gene expression datapoints (4 animal species × 4 stimuli × 11 cell types = 176 conditions).

**Supplementary Fig. S3**. Classification of genes according to species-specific expression patterns (related to Fig. 2). (A) Heatmap representing a latent factor matrix related to stimuli. The columns indicate stimuli, and the rows indicate latent factors representing stimulus-common (L2_1) and virus vs. LPS (L2_2) factors. (B) Heatmap representing a latent factor matrix related to cell types. The columns indicate cell types, and the rows indicate latent factors representing cell type–common (L3_1), monocyte-specific (L3_2), and B-cell–specific (L1_3) factors. (C) Summary of the normalization of values and patterning according to the ranking of the values. First, 6 column vectors (2 stimuli × 3 cell types) in the TD results were normalized by dividing them by the 90th percentile of the individual vectors. Then, data with >1 or <−1 were assigned as 1 and −1, respectively. Next, the genes were categorized into 3 classes (high, mid, and low) based on the rule that if the rank of a value was greater than the 80th percentile or smaller than the 20th percentile, it was categorized as “high” or “low,” respectively; otherwise, it was categorized as “mid.” (D) Summary of the ideal patterns for each gene category used in the gene classification in Fig. 2C. (E) Summary of the calculation of the similarity score and establishment of the threshold for the gene classification in Supplementary Fig. S3F. The sum of the residual squares between 2 vectors, the genewise vector of normalized values from the TD results (Supplementary Fig. S3C), and the “ideal patterns” (Supplementary Fig. S3D) were calculated. Then, the threshold used in Supplementary Fig. S3F was obtained by calculating the 20th percentile of the similarity score for the vector for each gene category. (F) Summary of gene classification. By comparing patterns from the TD results (Supplementary Fig. S3C) and the ideal patterns (Supplementary Fig. S3D), candidate gene categories were selected. Next, the gene category with the lowest “similarity score” among the candidate gene categories was selected as the tentative gene category. In this selection, if the “similarity score” was higher than the threshold of the gene category (Supplementary Fig. S3E), the gene was categorized as “Others” (gene B). If no candidate gene category was available, the gene was also classified as “Others” (gene C). Finally, the final gene category was determined by integrating similar gene categories (genes A and D). (G–I) Heatmap representing the values of the products calculated in Fig. 2C. The data relating to (G) the species-common factor (L1_1), (H) the bat-specific factor (L1_2), and (I) the macaque-specific factor (L1_3) are shown. Each row indicates the respective gene. The color keys shown on the right of the heatmap indicate gene categories. (J–L) Heatmap representing the FC values in the input tensor. The orders of the rows are the same as in (J) Supplementary Fig. S3G, (K) Supplementary Fig. S3H, and (L) Supplementary Fig. S3I. Each row indicates the respective gene. The color keys shown on the right of the heatmap indicate gene categories.

**Supplementary Fig. S4**. Identification of species-specific cell types (related to Fig. 4). (A) UMAP plots representing the expression patterns of every single cell. Dimensionality reduction was performed for each combination of the 4 species and 3 cell types. (B) UMAP plots representing the average expression levels of marker genes for cluster 7 [C7markers].

**Supplementary Table S1**. Primers used for RT-qPCR (related to the Methods). The sequences of the primers used for RT-qPCR are listed.

## Abbreviations

cDCs: conventional dendritic cells; CIU: cell infectious unit; CP10k: counts per 10,000 counts in the cell; DEGs: differentially expressed genes; DMEM: Dulbecco’s modified Eagle’s medium; dsRNAs: double-stranded RNAs; FC: fold change; FCS: fetal calf serum; FDR: false discovery rate; GEMs: gel beads-in-emulsion; GEO: Gene Expression Omnibus; GSVA: gene set variation analysis; GO: Gene Ontology; HOI: higher-order orthogonal iteration; Hs: *Homo sapiens*; HSV-1: herpes simplex virus type 1; IFNs: interferons; ISGs: IFN-stimulated genes; LPS: lipopolysaccharide; mad: median absolute deviation; MAITs: mucosal-associated invariant T cells; Mm: *Macaca mulatta*; Monos: monocytes; NK: natural killer; PAMPs: pathogen-associated molecular patterns; PBMCs: peripheral blood mononuclear cells; PC: principal component; PCA: principal component analysis; pDCs: plasmacytoid dendritic cells; PFU: plaque-forming unit; QC: quality control; PRRs: pattern recognition receptors; Pt: *Pan troglodytes*; Ra: *Rousettus aegyptiacus*; RLRs: RIG-I–like receptors; scRNA-seq: single-cell RNA sequencing; SeV: Sendai virus; TD: Tucker decomposition; UMAP: uniform manifold approximation and projection; UMI: unique molecular identifier.

## References

[1] Huff JL, Barry PA. B-virus (Cercopithecine herpesvirus 1) infection in humans and macaques: potential for zoonotic disease. Emerg Infect Dis. 2003;9(2):246–50.. 10.3201/eid0902.020272. 12603998 PMC2901951

[2] Letko M, Seifert SN, Olival KJ, et al. Bat-borne virus diversity, spillover and emergence. Nat Rev Micro. 2020;18(8):461–71.. 10.1038/s41579-020-0394-z. PMC7289071 32528128

[3] Towner JS, Amman BR, Sealy TK, et al. Isolation of genetically diverse Marburg viruses from Egyptian fruit bats. PLoS Pathog. 2009;5(7):e1000536. 10.1371/journal.ppat.1000536. 19649327 PMC2713404

[4] Guito JC, Prescott JB, Arnold CE, et al. Asymptomatic infection of Marburg virus reservoir bats is explained by a strategy of immunoprotective disease tolerance. Curr Biol. 2021;31(2):257–70..e5. 10.1016/j.cub.2020.10.015. 33157026

[5] Akira S, Uematsu S, Takeuchi O. Pathogen recognition and innate immunity. Cell. 2006;124(4):783–801.. 10.1016/j.cell.2006.02.015. 16497588

[6] Takeuchi O, Akira S. Pattern recognition receptors and inflammation. Cell. 2010;140(6):805–20.. 10.1016/j.cell.2010.01.022. 20303872

[7] Hansen JD, Vojtech LN, Laing KJ. Sensing disease and danger: a survey of vertebrate PRRs and their origins. Dev Comp Immunol. 2011;35(9):886–97.. 10.1016/j.dci.2011.01.008. 21241729

[8] Lu Y, Su F, Li Q et al. Pattern recognition receptors in Drosophila immune responses. Dev Comp Immunol. 2020;102:103468. 10.1016/j.dci.2019.103468. 31430488

[9] Dempsey A, Bowie AG. Innate immune recognition of DNA: a recent history. Virology. 2015;479-480:146–52.. 10.1016/j.virol.2015.03.013. 25816762 PMC4424081

[10] Poltorak A, He X, Smirnova I, et al. Defective LPS signaling in C3H/HeJ and C57BL/10ScCr mice: mutations in Tlr4 gene. Science. 1998;282:2085–8.. 10.1126/science.282.5396.2085. 9851930

[11] Schountz T, Baker ML, Butler J, et al. Immunological control of viral infections in bats and the emergence of viruses highly pathogenic to humans. Front Immunol. 2017;8:1098. 10.3389/fimmu.2017.01098. 28959255 PMC5604070

[12] Gorbunova V, Seluanov A, Kennedy BK. The world goes bats: living longer and tolerating viruses. Cell Metab. 2020;32(1):31–43.. 10.1016/j.cmet.2020.06.013. 32640245 PMC7341951

[13] Banerjee A, Baker ML, Kulcsar K et al. Novel insights into immune systems of bats. Front Immunol. 2020;11:26. 10.3389/fimmu.2020.00026. 32117225 PMC7025585

[14] Pavlovich SS, Lovett SP, Koroleva G, et al. The Egyptian rousette genome reveals unexpected features of bat antiviral immunity. Cell. 2018;173(5):1098–110..e18. 10.1016/j.cell.2018.03.070. 29706541 PMC7112298

[15] Zhou P, Tachedjian M, Wynne JW, et al. Contraction of the type I IFN locus and unusual constitutive expression of IFN-α in bats. Proc Natl Acad Sci USA. 2016;113(10):2696–701.. 10.1073/pnas.1518240113. 26903655 PMC4790985

[16] Xie J, Li Y, Shen X et al. Dampened STING-dependent interferon activation in bats. Cell Host Microbe. 2018;23(3):297–301..e4. 10.1016/j.chom.2018.01.006. 29478775 PMC7104992

[17] Ahn M, Cui J, Irving AT, et al. Unique loss of the PYHIN gene family in bats amongst mammals: implications for inflammasome sensing. Sci Rep. 2016;6:21722. 10.1038/srep21722. 26906452 PMC4764838

[18] Irving AT, Zhang Q, Kong PS et al. Interferon regulatory factors IRF1 and IRF7 directly regulate gene expression in bats in response to viral infection. Cell Rep. 2020;33(5):108345. 10.1016/j.celrep.2020.108345. 33147460 PMC8755441

[19] De La Cruz-Rivera PC, Kanchwala M, Liang H, et al. The IFN response in bats displays distinctive IFN-stimulated gene expression kinetics with atypical RNASEL induction. J Immunol. 2018;200(1):209–17. 29180486 10.4049/jimmunol.1701214 PMC5736455

[20] Lin HH, Horie M, Tomonaga K. A comprehensive profiling of innate immune responses in Eptesicus bat cells. Microbiol Immunol. 2022;66(3):97–112.. 10.1111/1348-0421.12952. 34842304

[21] Ahn M, Anderson DE, Zhang Q, et al. Dampened NLRP3-mediated inflammation in bats and implications for a special viral reservoir host. Nat Microbiol. 2019;4(5):789–99.. 10.1038/s41564-019-0371-3. 30804542 PMC7096966

[22] Goh G, Ahn M, Zhu F, et al. Complementary regulation of caspase-1 and IL-1β reveals additional mechanisms of dampened inflammation in bats. Proc Natl Acad Sci USA. 2020;117(46):28939–49.. 10.1073/pnas.2003352117. 33106404 PMC7682399

[23] Seurat . Seurat (version 4.0.4). *GitHub*. 2021. https://github.com/satijalab/seurat/releases/tag/v4.0.4

[24] Hao Y, Hao S, Andersen-Nissen E, et al. Integrated analysis of multimodal single-cell data. Cell. 2021;184(13):3573–87..e29. 10.1016/j.cell.2021.04.048. 34062119 PMC8238499

[25] Azimuth . Azimuth (version 0.4.3). *GitHub*. 2020. https://github.com/satijalab/azimuth/releases/tag/v0.4.3

[26] Friedrichs V, Toussaint C, Schäfer A et al. Landscape and age dynamics of immune cells in the Egyptian rousette bat. Cell Rep. 2022;40(10):111305. 10.1016/j.celrep.2022.111305. 36070695

[27] Banerjee A, Rapin N, Bollinger T et al. Lack of inflammatory gene expression in bats: a unique role for a transcription repressor. Sci Rep. 2017;7(1):2232. 28533548 10.1038/s41598-017-01513-w PMC5440382

[28] Shaw AE, Hughes J, Gu Q et al. Fundamental properties of the mammalian innate immune system revealed by multispecies comparison of type I interferon responses. PLoS Biol. 2017;15(12):e2004086. 10.1371/journal.pbio.2004086. 29253856 PMC5747502

[29] Sato R, Kato A, Chimura T et al. Combating herpesvirus encephalitis by potentiating a TLR3-mTORC2 axis. Nat Immunol. 2018;19(10):1071–82.. 10.1038/s41590-018-0203-2. 30201994

[30] Shah S, King EM, Chandrasekhar A, et al. Roles for the mitogen-activated protein kinase (MAPK) phosphatase, DUSP1, in feedback control of inflammatory gene expression and repression by dexamethasone. J Biol Chem. 2014;289(19):13667–79.. 10.1074/jbc.M113.540799. 24692548 PMC4036371

[31] Li QJ, Chau J, Ebert PJ et al. miR-181a is an intrinsic modulator of T cell sensitivity and selection. Cell. 2007;129(1):147–61.. 10.1016/j.cell.2007.03.008. 17382377

[32] Posselt G, Schwarz H, Duschl A et al. Suppressor of cytokine signaling 2 is a feedback inhibitor of TLR-induced activation in human monocyte-derived dendritic cells. J Immunol. 2011;187(6):2875–84.. 10.4049/jimmunol.1003348. 21844389

[33] MSigDB (version 7.3) . GSEA and MSigDB Team, Broad Institute, Massachusetts. 2022.; https://www.gsea-msigdb.org/gsea/msigdb/. Accessed 1 Sept 2022.

[34] Seal S, Dharmarajan G, Khan I. Evolution of pathogen tolerance and emerging infections: a missing experimental paradigm. eLife. 2021;10:e68874. 10.7554/eLife.68874. 34544548 PMC8455132

[35] Lafaille FG, Pessach IM, Zhang SY et al. Impaired intrinsic immunity to HSV-1 in human iPSC-derived TLR3-deficient CNS cells. Nature. 2012;491:769–73.. 10.1038/nature11583. 23103873 PMC3527075

[36] López-Cotarelo P, Gómez-Moreira C, Criado-García O, et al. Beyond chemoattraction: multifunctionality of chemokine receptors in leukocytes. Trends Immunol. 2017;38(12):927–41.. 10.1016/j.it.2017.08.004. 28935522

[37] Abcam . Chemokines and Their Receptors. Cambridge, UK: Abcam; 2021. https://docs.abcam.com/pdf/immunology/chemokines_poster.pdf. Accessed 1 Sept 2022.

[38] Ejercito PM, Kieff ED, Roizman B. Characterization of herpes simplex virus strains differing in their effects on social behaviour of infected cells. J Gen Virol. 1968;2(3):357–64.. 10.1099/0022-1317-2-3-357. 4300104

[39] Yoshida A, Kawabata R, Honda T, et al. A single amino acid substitution within the paramyxovirus Sendai virus nucleoprotein is a critical determinant for production of interferon-beta-inducing copyback-type defective interfering genomes. J Virol. 2018;92(5):e02094–17.. 10.1128/JVI.02094-17. 29237838 PMC5809723

[40] Kiyotani K, Sakaguchi T, Kato A et al. Paramyxovirus Sendai virus V protein counteracts innate virus clearance through IRF-3 activation, but not via interferon, in mice. Virology. 2007;359(1):82–91.. 10.1016/j.virol.2006.08.053. 17027894

[41] Yamada E, Nakaoka S, Klein L, et al. Human-specific adaptations in Vpu conferring anti-tetherin activity are critical for efficient early HIV-1 replication in vivo. Cell Host Microbe. 2018;23(1):110–20.e7.. 10.1016/j.chom.2017.12.009. 29324226

[42] National Center for Biotechnology Information . RefSeq. 2020. https://www.ncbi.nlm.nih.gov/refseq/. Accessed 6 Nov 2020.

[43] 10X Genomics . Build a custom reference (cellranger mkref). 2020. https://support.10xgenomics.com/single-cell-gene-expression/software/pipelines/latest/using/tutorial_mr. Accessed 6 Nov 2020.

[44] National Center for Biotechnology Information . The Report of Orthologous Genes. 2021. https://ftp.ncbi.nih.gov/gene/DATA/gene_orthologs.gz. Accessed 26 July 2021.

[45] Jebb D, Huang Z, Pippel M, et al. Six reference-quality genomes reveal evolution of bat adaptations. Nature. 2020;583:578–84.. 10.1038/s41586-020-2486-3. 32699395 PMC8075899

[46] Bat1K Project . 2018. https://bat1k.com. Accessed 14 July 2021.

[47] University of California, Santa Cruz . UCSC Genome Browser. 2000. https://genome.ucsc.edu. Accessed 14 July 2021.

[48] Quinlan AR, Hall IM. BEDTools: a flexible suite of utilities for comparing genomic features. Bioinformatics. 2010;26(6):841–2.. 10.1093/bioinformatics/btq033. 20110278 PMC2832824

[49] Cell Ranger . Cell Ranger (version 6.0.1). 2021. https://support.10xgenomics.com/single-cell-gene-expression/software/downloads/6.0. Accessed 20 May 2021.

[50] Zheng GXY, Terry JM, Belgrader P et al. Massively parallel digital transcriptional profiling of single cells. Nat Commun. 2017;8:14049. 10.1038/ncomms14049. 28091601 PMC5241818

[51] McInnes L, Healy J, Melville J. UMAP: uniform manifold approximation and projection for dimension reduction. 2018. arXiv:1802.03426. 10.48550/arXiv.1802.03426.

[52] TensorLy . TensorLy (version 0.6.0). *GitHub*. 2021. https://github.com/tensorly/tensorly/releases/tag/0.6.0.

[53] rTensor . rTensor (version 1.4.8). *GitHub*. 2022. https://github.com/rikenbit/rTensor/releases/tag/v1.4.8.

[54] GSVA . GSVA (version 1.38.2). *Bioconductor*. 2021. https://doi.org/doi:10.18129/B9.bioc.GSVA.

[55] Hänzelmann S, Castelo R, Guinney J. GSVA: gene set variation analysis for microarray and RNA-seq data. BMC Bioinform. 2013;14:7. 10.1186/1471-2105-14-7. PMC3618321 23323831

[56] Aso H, Ito J, Ozaki H et al. scRNA-seq_pbmc_animals_aso_et_al. GitHub. 2023. https://github.com/TheSatoLab/scRNA-seq_PBMC_Animals_Aso_et_al.

[57] National Institutes of Health . The Human BioMolecular Atlas Program (HuBMAP). 2020. https://azimuth.hubmapconsortium.org/references/#Human%20-%20PBMC. Accessed 26 July 2021.

[58] Aso H, Ito J, Ozaki H, et al. Single-cell_transcriptome_analysis_illuminating_the_characteristics_of_species-specific_innate_immune_responses_against_viral_infections_Aso_et_al. Mendeley Data. 2023. 10.17632/kg3dfkyjv5.2

[59] Aso H, Ito J, Ozaki H, et al. Supporting data for “Single-Cell Transcriptome Analysis Illuminating the Characteristics of Species-Specific Innate Immune Responses against Viral Infections.”. GigaScience Database. 2023.; 10.5524/102448. PMC10580374 37848618

