# Supplementary material for: Single-cell transcriptome analysis illuminating the characteristics of species-specific innate immune responses against viral infections
Source: Gigascience. 2023 Oct 17;12:giad086. doi: 10.1093/gigascience/giad086 (PMC10580374; doi:10.1093/gigascience/giad086)
Supplement: giad086_GIGA-D-23-00007_Revision_1 [file giad086_giga-d-23-00007_revision_1.pdf]

GigaScience

Single-cell transcriptome analysis illuminating the characteristics of species-specific innate immune responses against viral infections

--Manuscript Draft--

**Manuscript Number:**	GIGA-D-23-00007R1
**Full Title:**	Single-cell transcriptome analysis illuminating the characteristics of species-specific innate immune responses against viral infections
**Article Type:**	Research
**Funding Information:**	Japan Science and Technology Corporation (CREST JPMJCR20H4) — Professor Kei Sato
**Abstract:**	Background<br>Bats harbor various viruses without severe symptoms and act as their natural reservoirs. The tolerance of bats against viral infections is assumed to originate from the uniqueness of their immune system. However, how immune responses vary between primates and bats remains unclear. Here, we characterized differences in the immune responses by peripheral blood mononuclear cells to various pathogenic stimuli between primates (humans, chimpanzees, and macaques) and bats (Egyptian fruit bats) using single-cell RNA sequencing.<br><br>Results<br>We show that the induction patterns of key cytosolic DNA/RNA sensors and antiviral genes differed between primates and bats. A novel subset of monocytes induced by pathogenic stimuli specifically in bats was identified. Furthermore, bats robustly respond to DNA virus infection even though major DNA sensors are dampened in bats.<br><br>Conclusions<br>Overall, our data suggest that immune responses are substantially different between primates and bats, presumably underlying the difference in viral pathogenicity among the mammalian species tested.
**Corresponding Author:**	Kei Sato<br>Tokyo Daigaku<br>Minato-ku, Tokyo JAPAN
**Corresponding Author Secondary Information:**	
**Corresponding Author's Institution:**	Tokyo Daigaku
**Corresponding Author's Secondary Institution:**	
**First Author:**	Hirofumi Aso
**First Author Secondary Information:**	
**Order of Authors:**	Hirofumi Aso
	Jumpei Ito
	Haruka Ozaki
	Yukie Kashima
	Yutaka Suzuki
	Yoshio Koyanagi
	Kei Sato
**Order of Authors Secondary Information:**	
**Response to Reviewers:**	*'Respond to Reviewers' is also prepared as a supplemental material (230812ResponseLetter_v12.docx). Please also find this file as well.

Powered by Editorial Manager® and ProduXion Manager® from Aries Systems Corporation

Reviewer #1: Hirofumi Aso and colleagues provide a manuscript entitled 'Single-cell transcriptome analysis illuminating the characteristics of species specific innate immune responses against viral infections'. The aim was to describe differences in innate immune responses of peripheral blood mononuclear cells (PBMCs) from different primates and bats against various pathogenic stimuli (different viruses and LPS). A major conclusion from the study is that differences in the immune response between primate and bat PBMCs are more pronounced than those between DNA, RNA viruses or LPS, or between the cell types.

The topic is of interest as the immunological basis for how bats appear to be largely disease resistant to some viruses that cause severe infections in humans is not well understood. One notion by others has been that bats have a larger spectrum of interferon (IFN) type I related genes, some of which are expressed constitutively even in unstimulated tissue, and there, trigger the expression of IFN stimulated genes (ISGs). Alongside, enhanced ISG levels may need to be compensated for in bats. Accordingly, bats may exhibit reduced diversity of DNA sensing pathways, as well as absence of a range of proinflammatory cytokines triggered in humans upon encountering acute disease causing viruses.

The study here uses single-cell RNA sequencing (scRNA-seq) analysis, and transcript clustering algorithms to explore the profile of different innate immune responses upon viral infections of PBMCs from H sapiens, Chimpanzee, Rhesus macaque, and Egyptian fruit bat. Most commonly referred to cell types were detected in all four species, although naïve CD8+ T cells were not detected in bat PBMCs, which led the authors to focus on B cells, naïve T cells, killer T/NK cells, monocytes, cDCs, and pDCs. The study used three pathogenic stimuli, Herpex simplex virus 1 (HSV1), Sendai virus (SeV), and lipopolysaccharide (LPS).

Specific comments

The text is well written, concise, and per se interesting, but I have a few questions for clarification.
Thank you very much for your high evaluation. We have responded to the points you raised and hope you recognize the improvements we have made.

1) Can the authors provide quality and purity control data for the virus inocula to document virus homogeneity? E.g., neither the methods, nor the indicated ref 26 specify if or how HSV1 was purified. Same is true for SeV where the provided ref 34 does not indicate if virus was purified or not. If virus inocula were not purified then it remains unclear to what extent the effects on the PBMCs described in the study here were due to virus or some other component in the inoculum. Conditions using inactivated inoculum might help to clarify this issue.
Thank you for pointing this out and we apologize for the lack of clarity in our descriptions regarding this point. In this study, we did not purify either HSV-1 or SeV. Thus, it is possible that something in the supernatant of virus-producing cells (including viruses) could affect the innate immune responses in PBMCs. However, given that the use of the supernatants without purification is a conventional virological method, any unanticipated effects would be much smaller than the effects of viral infection. To make it clear, we have added a description about this information in the revised manuscript in Materials and Methods (Lines 509-510, 522-523).

Lines 509-510: “Briefly, Vero cells were infected with HSV-1 and the supernatant was collected and used without purification.”

Lines 522-523: “Briefly, LLC-MK2 cells were infected with SeV and the supernatant was collected and used without purification.”

2) What was the infection period? Was it the same for all viruses?
Samples were analyzed at one day post infection. This is a condition shared by all viruses and stimuli, described in Lines 539-541 (Method: Infection and stimulation) in the revised manuscript. To emphasize that they are the same conditions, minor

Powered by Editorial Manager® and ProduXion Manager® from Aries Systems Corporation

corrections have been made to the revised manuscript (Lines 129-133 and Lines 539-541).

Lines 129-133: “To analyze immune responses to stimuli at single-cell resolution, we performed scRNA-seq analysis of 16 types of PBMC samples: four mammalian species (Hs, Pt, Mm, and Ra) versus four conditions (mock infection/stimulation, HSV-1 infection, SeV infection, and LPS stimulation) using the 10x Genomics Chromium platform at one day post infection.”

Lines 539-541: “At one day post infection, all types of infected/stimulated PBMCs were centrifuged, resuspended in PBS, and used for bulk RT–qPCR and scRNA-seq (see below).”

3) Upon stimuli application, there was a noteable expansion of B cells and a compression of killer T / NK cells in the bat but not the human samples, as well as compression of monocytes, the latter observed in all four species. Can the authors comment on this observation?
Thank you for your valuable suggestion. According to the reviewer’s comment, we have edited the main text in the revised manuscript as follows (Lines 168-175, 326-328):

Lines 168-175: “The frequency of monocytes decreased after stimulation in all four species, whereas the frequency of B cells and killer TNK cells changed differently among the animal species and stimuli. Upon stimulation, there was generally a notable increase in B cells and a decrease of killer TNK cells in the bat (and non-human primates) samples, but not in the human samples. After SeV infection, the frequency of B cells was decreased in all four species. On the other hand, after HSV-1 infection, the frequency of B cells was decreased in only humans.”

Lines 326-328: “It is also noteworthy that changes in cell composition differed between humans and bats (Fig. 1D).”

4) Lines 78-79: I do not think that TLR9 ought to be classified as a cytosolic DNA sensor. Please clarify.
Thank you for pointing this out. It is true that this wording was incorrect. Therefore, we fixed the issues in the manuscripts (Lines 80-82).

Lines 80-82: “Cytosolic Extrachromosomal DNAs, a PAMP for DNA viruses, are recognized by cytosolic DNA sensors, such as (e.g., cGAS, AIM2, and IFI16), and endosomal DNA sensors (e.g., TLR9)5,6,9 [5, 6, 9].”

5) Line 117: please clarify that the upregulation of proinflammatory cytokines, ISGs and IFNB1 was measured at the level of transcripts not protein.
Thank you for pointing this out. According to the reviewer’s suggestion, we clarified that the upregulation of those factors was measured at the level of mRNA transcripts (Lines 127-128).

Lines 127-128: "IFNB1 (Fig. S1A–C) at the level of mRNA transcripts.“

6) Line 244: DNA sensors. Authors report that bats responded well to DNA viruses, although some of their DNA sensing pathways (e.g., STING downstream of cGAS, AIM2 or IFI16) were attenuated compared to primates (H sapies, Chimpanzee, Macaque). And they elute to the dsRNA PRR TLR3. But I am not sure if TLR3 is the only PRR to compensate for attenuated DNA sensing pathways. The authors might want to explicitly discuss if other RNA sensors, such as RIG-I-like receptors (RIG-I, LGP2, MDA5) were upregulated similarly in bats as in primate cells upon inoculation with HSV1.
Thank you for the important suggestions. As you have pointed out, it is possible that PRRs other than TLR3 compensates for weak DNA sensing pathways for HSV-1. We specially mentioned TLR3 in the original manuscript because the importance of human TLR3 in the response to HSV-1 has been demonstrated (Sato, Kato et al., Nature Immunology, 2018). However, we agree with your opinion that other possibilities should also be mentioned and have added them in the revised manuscript (Lines 344-354).

Powered by Editorial Manager® and ProduXion Manager® from Aries Systems Corporation

Lines 344-354: “An alternative possibility is that the IFN response in response to HSV-1 infection was triggered by sensing viral molecules other than DNAs: iIt is known that, in humans and mice, dsRNA sensing by TLR3 plays an important role in responding to HSV-1 infection26,31. Furthermore Additionally, the Egyptian fruit bat genome encodes an intact TLR3 gene (NCBI Gene ID: 107510436), and bat immune cells express TLR3 (Fig. 3B). Furthermore, other RNA sensors, such as RIG-I, LGP2, and MDA5, were upregulated in bat cells similarly as in primate cells upon HSV-1 infection (Fig. 3B). These data suggest that in bats, bat TLR3 or other RNA sensors in bats may compensate for the immune responses induced by weakened DNA sensing pathwaysors, leading to IFN responses to HSV-1 infection.”

7) Is it known how much TLR3 protein is expressed in bat PBMCs under resting and stimulated conditions? Same question for the DNA and RNA sensor proteins, e.g., cGAS, AIM2 or IFI16, RIG-I, LGP2, MDA5, or effector proteins, such as STING.
To our knowledge, there are no publications quantifying the expression of TLR3 or other sensor genes in bats at the protein level. This is mainly due to the lack of antibodies recognizing these proteins of bats.

8) Can authors clarify if cGAS is part of the attenuated DNA sensors in the bat samples under study here? And it would be nice to see the attenuated response of DNA sensing pathways in the bat samples, as suspected from the literature, including STING downstream of cGAS, or AIM2 and IFI16.
Thank you for these suggestions. We address these two points as follows:

> Can authors clarify if cGAS is part of the attenuated DNA sensors in the bat samples under study here?
In a previous study (Xie et al., Cell Host & Microbe, 2018), the cGAS signaling pathway is reduced by a mutation in STING (not in cGAS), and this mutation is conserved in bat species including Rousettus aegyptiacus, the bat species analyzed in this study. Therefore, the cGAS signaling pathway should be reduced in the bat PBMC samples in this study. Thus, to clarify this point, we modified the text in the revised manuscript (Lines 335-337).

Lines 335-337: “It is known that two DNA sensing pathways mediated by cGAS-STING pathway [16]16 and PYHIN proteins, including AIM2 and IFI16 [17]17, are dampened in bats, including Egyptian fruit bats.”

> And it would be nice to see the attenuated response of DNA sensing pathways in the bat samples, as suspected from the literature, including STING downstream of cGAS, or AIM2 and IFI16.
We agree this is a good idea. Unfortunately, it is difficult to evaluate the activity of each DNA sensing pathway using the limited number of bat samples in this study. Further studies are required.

9) What are the expression levels of IFN-I and related genes in the bat cells among the different stimuli?
Thank you very much for your constructive comments. We agree it is important to examine the expression level of IFN-I itself when discussing innate immune responses. Unfortunately, however, we could not obtain the expression level of IFN-α genes in bat samples because IFN-α genes were not included in the gene annotations of Rousettus aegyptiacus (both RefSeq and Bat1K). In addition, although we tried to measure the expression level of IFN-α genes in bat samples using the custom gene annotation including bat IFN-α genes, we failed to detect the expression of these genes (Figure R1A–F). Therefore, we did not focus on the expression of IFN-I genes in this study. Instead, we discussed the expression level of ISGs as a surrogate for the activity of IFN-I pathway as shown in Fig. 3A. We added this explanation in Results in the revised manuscript (Lines 261-268).

Lines 261-268: “To test this hypothesis, we analyzed the IFN response upon HSV-1 (a DNA virus) infection. However, the expression levels of IFN-α genes were not examined because they were not annotated in the transcript model for the Egyptian fruit bat used in this study, and the expression of IFN-β genes were too low. Thus, even though the expression levels of IFN-I is the primary factor to examine the activity

of the IFN response, we instead by analyzing analyzed the induced levels of "coremamm ISGs"—a set of genes that are commonly induced by type I IFNs across mammals that were defined in a previous study 25 [28]."

Note: Although HSV-1-infected bat sample showed high expression levels of IFNA3 and IFNB1 (Figure R1E), only one cell expressed most of IFNA3 and IFNB1 (Figure R1F). Therefore, this cell was determined to be an outlier.

Figure R1. Difficulties in quantitative analysis of expression levels of IFN-α in bat samples
(A) Schematic of the IFN-I loci near the KLHL9 locus, originated from Zhou et al. [PMID: 26903655]. (B) Information extracted from the transcript model (gtf file) of Rousettus aegyptiacus in the initial manuscript. (C) Results of blastn for IFN-α sequences near the KLHL9 locus. Query: Rousettus aegyptiacus IFN-α (GenBank: AB259762.1). Subject: mRouAeg1.p "NW_023416306.1:61702666-62433637". Red indicates the alignment patterns with high mapping scores. (D) Information extracted from custom gene annotation used in E and F. (E, F) Results of mapping and counting by CellRanger using custom gene annotation. (E) Sum of counts for each bat sample. (F) Distribution of counts per cell for IFNA3 (top) and IFNB1 (bottom) in HSV-1-infected bat sample.

10) Technical point: where can the raw scRNA-seq data be found?
As stated in Data and code availability, the raw scRNA-seq data is found in NCBI GEO (GSE218199). To clarify this, we modified the text in the revised manuscript (Lines 837-838).

Lines 837-838: "The raw and processed Ssingle-cell RNA-seq data have been deposited in the Gene Expression Omnibus (GEO) database (GSE218199) and are publicly available."

Reviewer #2: This paper gives a good introduction on bats as reservoirs of several viral infections, which studies have shown is due to the uniqueness of their immune system. They and others suggest that bats immune system is dampened exhibiting tolerance to various viruses. This gives the study a good rationale as to why study the bats immune system, compared to other mammals. They also give a good rationale as to why they used single-cell sequencing, to allow the identification of various cell types and the differences in these cell types. From their finding the main conclusions are that differences in the host species are more impactful; than those among the different stimuli. They also suggest that bats initiate an innate immune response after infection with DNA viruses through an alternative pathway. For example, the induction dynamics of PRRs seems to be different in their dataset. They also suggest this could be due to the presence of species-specific cellular subsets.

1. Interesting model system and a good comparison of bats with other mammals.
2. Good technique in using single-cell sequencing, with a clear rationale as to why it was chosen. This advances knowledge on what was already known about bats immune system, but the species-specific cellular subsets are new.
3. Interesting technique to go through the bulk transcriptomic data in four species and four conditions. This allowed findings of the most important genes/pathways.
4. Good rationale / flow of experiments from one to another
5. I liked that they investigated stimuli from different pathogens , including DNA, RNA virus and bacteria and still show that bats had a different immune system, in the different stimuli.
Thank you for your appreciation on various points. Based on your comments, we have made modifications to improve the manuscript.

Powered by Editorial Manager® and ProduXion Manager® from Aries Systems Corporation

Minor comments
1. Do they speculate this occurrence in is this just in Egyptian Fruit bats or all species of bats?
Although we consider that the differences between Rousettus aegyptiacus and primates observed in this study mainly originate from those between bats and primates, we agreed that there should be differences among bat species. To clarify this point, we added some descriptions to the Discussion (Limitations of the study) in the revised manuscript (Lines 407-410).

Lines 407-410: "Moreover, because the results of this study rely on an analysis using a single bat species, the Egyptian fruit bat, it is unclear whether the observed bat-specific characteristics are conserved across bat species."

2. Mentioned in the introduction why they used the egyptian fruit bats - which are a model organism, but this could help people who are not in this field understand exactly why use these bats. Advantages? Location? Proximity to the various viruses based on the fact they are mostly found in endemic regions such as Africa etc.
Thank you for mentioning this important point. Indeed, the description of the reason for using the Egyptian Fruit bats was insufficient. The first reason why we selected this bat species is that they were available because they were captive bred. The second reason is that they are a species known to be asymptomatically infected with human pathogenic viruses, such as Marburg virus. We have stated this point in the Introduction and Results in the revised manuscript (Lines 107-111 and Lines 119-122).

Lines 107-111: "Here, we used peripheral blood mononuclear cells (PBMCs) from four mammalian species including the Egyptian fruit bats and three pathogenic stimuli and conducted single-cell RNA sequencing (scRNA-seq) analysis to elucidate the differences in innate immune responses against pathogenic stimuli."

Lines 119-122: "In this study, the Egyptian fruit bat was used as a representative model organism for bat species because this bat species is bred and available in captivity and is known as natural host of human pathogenic viruses, such as Marburg virus [3]."

3. Can they include viral load in each species?
"Viral load" was not quantified, but the amount of RNA in the collected samples was quantified by RT-qPCR (Fig. S1B–C).

4. It is not clear which scRNAseq tools were used for data analysis in identifying the types of cells. Or did they use already established database based on markers?
Thank you for this comment. Details of the method can be found in the Cell Annotation section of Methods. In short, this study uses Seurat and Azimuth to annotate each cluster resulting from unsupervised clustering based on several pieces of information (Fig. S2A–F). In Azimuth, we also use reference data created based on markers (https://azimuth.hubmapconsortium.org). However, since it was written only in Methods not written in Results, we have now added a description in Results in the revised manuscript (Lines 143-145).

Lines 143-145: "We characterized the cellular composition of PBMCs from each mammalian species by annotating the cell type of individual single cells using tools available in Seurat [23, 24] and Azimuth [25] (See Methods)."

Anonymous Reviewer: I find after reading the MS that under my normal reviewer conditions I would have no choice but to reject the manuscript.

The structure and design of the MS is ok normally however the actual data has problems in that there is only n=1 sample. For each species (n=4) and each condition (n=4) there are indeed n=16 lots of single cell data. This however means there isn't even a single repeat. There is so much variation within bats, let alone between treatments or between species that simply n=1 for any scientific experiment is not acceptable. Further there are 40,717 cells detected between 16 separate experiments leaving a very low number of cells.

If you look at the raw data for fig S1D, you can see the cell number for bats is incredibly low.

Powered by Editorial Manager® and ProduXion Manager® from Aries Systems Corporation

Rousettus_aegyptiacusMockB1549
Rousettus_aegyptiacusMockNaiveT541
Rousettus_aegyptiacusMockKillerTNK1960
Rousettus_aegyptiacusMockMono1035
Rousettus_aegyptiacusMockcDC57
Rousettus_aegyptiacusMockpDC10
Rousettus_aegyptiacusHSV1B767
Rousettus_aegyptiacusHSV1NaiveT166
Rousettus_aegyptiacusHSV1KillerTNK347
Rousettus_aegyptiacusHSV1Mono71
Rousettus_aegyptiacusHSV1cDC3
Rousettus_aegyptiacusHSV1pDC2
Rousettus_aegyptiacusSeVB1160
Rousettus_aegyptiacusSeVNaiveT48
Rousettus_aegyptiacusSeVKillerTNK116
Rousettus_aegyptiacusSeVMono137

Gigascience prides itself on high-quality datasets but this set is way too minimal. I feel the data quality is well below GigaScience's level. Its a real pity as the manuscript is otherwise written and presented well - with a logical flow and decent structure for analysing the data.
Regarding biological replicates:
We agree with the importance of biological replicates, but it is cost-prohibitive to perform scRNA-seq for 16 samples with replicates. For clarity, we have now added the fact that we did not have biological replicates in the Limitations of the study section (Line 410).

Line 410: "Furthermore, we did not perform biological replicates of scRNA-seq in this study."

Regarding your point about low cell numbers:
The average cell numbers were about 2,500 cells/run after quality control (QC). Technically, it is possible to obtain higher cell numbers (e.g. more than 10,000 cells/run) before QC, but the higher the value, the higher the percentage of cell multiplets. That's why in this study, we targeted a concentration preparation of 5,000 cells/run before QC. Furthermore, since this study used stimulation by viral infection and cells from animals other than humans, it was expected that the value would be lower than the theoretical value. Considering these factors, we believe that the average of 2500 cells/run after QC, which is 1/2 of the target value before QC, is sufficiently high. Based on these considerations and your feedback, we have now added descriptions of conditions (cell concentration and number of target cells) to Methods in the revised manuscript (Lines 557-558).

Lines 557-558: "Before loading, cell numbers and viability were confirmed. To acquire 5,000 cells recovery, 8,000 cells were loaded."

There are problems with the methods aswell when the authors made certain decisions without any explanation or discussion (or reference for similar) e.g. "Cells with 800–5,000 genes/cell or 1,200–25,000 counts/cell were extracted." "Second, we removed nontargeted cells in the present study." "Finally, regarding genes/cell and counts/cell values, cells with >3 |Z score| were excluded.". This could then bias the data (and indeed seems to whereby the majority of lower-count cells for SeV are removed before and after QC - this obviously suggests Sev-infection reduces gene transcription in cells but these cells are filtered out.
Thank you for pointing this out. The cell filtering pipeline and its threshold have been set to ensure valid comparisons. As you mentioned, there was a group of cells with significantly low genes/cell and counts/cell values in Bat SeV. One possible interpretation of this could be that SeV infection may suppress the gene expression of these cells. However, it is reasonable to exclude these cells in which lower number of genes are detected from the analysis because it would be problematic to perform the analysis if these cells are included in the downstream analysis. Thus, we added the explanation why these cells were excluded in Results (Lines 133-139) and Methods (Lines 637-649) in the revised manuscript. Next, the cell types excluded from the analysis are erythrocytes, platelets, hematopoietic stem cells, and innate lymphoid

	cells. For erythrocytes and platelets, we removed them because these cell types are not members of PBMCs. Hematopoietic stem cells and innate lymphoid cells were excluded because they are not major cell types when discussing innate immune responses in PBMCs. We have also now added an explanation about this in Methods in the revised manuscript (Lines 637-649).<br><br>Lines 133-139: “Next, quality control (QC) was performed to exclude both cells with lower data quality and cells not targeted in this study (Fig. S1D–G) (See Methods). Before QC, there was a group of cells with low genes-per-cell and counts-per-cell in PBMCs of SeV-infected bats (Fig. S1D–E). Although one possible interpretation of this could be that SeV infection may suppressed the gene expression in these cells, these cells were excluded to ensure the integrity of the downstream quantitative analysis.”<br><br>Lines 637-649: “The thresholds were determined based on the distributions of genes/cell and counts/cell before QC (Fig. S1D–E). Second, we removed nontargeted cells in the present study. We annotated the cell type of individual cells using Azimuth (v0.4.3) [25], a reference-based cell annotation prediction program (https://azimuth.hubmapconsortium.org), and then, cells annotated as erythrocytes, platelets, hematopoietic stem cells, or innate lymphoid cells, and platelets were excluded as nontargeted cells in the present study. This is because erythrocytes and platelets are probably residuals after experimental PBMC extraction, and hematopoietic stem cells and innate lymphoid cells are not the major cell types in the analysis of innate immune responses using PBMCs. In this step, the gene annotation “genes shared with humans” (see Gene annotation and ortholog information) for each animal species was used. Finally, regarding genes/cell and counts/cell values, cells with >3 |Z score| were excluded as outliers.”<br><br>The very limited dataset then also has different methods for PBMC extraction applied to different species (particularly whereby the anaesthesia used in chimpanzee is known to affect profile of PBMCs). Yet these cells are all dumped together for comparisons of human only or bat only genes.<br>As you pointed out, the method of obtaining PBMCs differs from animal to animal. However, this is in accordance with the characteristics of the animals, so it is unavoidable from an ethical point of view. Also, the main analysis is about the differences between bats and mammals and does not mention chimpanzee-specific characteristics. Furthermore, the results that chimpanzee cells showed similar expression patterns to those of humans and different from those of macaques and bats are consistent with the fact that chimpanzees are evolutionally close to humans. The results also support the idea that anesthesia did not have a significant effect. In conclusion, we believe that the method of PBMC acquisition in this study was appropriate.<br><br>There are other minor problems whereby the count normalization (post-transformation) is merged for all 4 species but after that differen settings (by species) a used in FindClusters (the idea of normalization should overcome this and allow similar settings to be used).<br>Because the hyperparameters of FindClusters are particularly sensitive to cell number, we used the hyperparameters that were adjusted for the cell number in each animal in this study.<br><br>They also perform statistics via FIshers exact test (despite n=1) for GO analysis in a rather unusual and creative way for GO of X against all others (rather than mock vs treatment) due to the lack of replicates.<br>In this study, we used an overlap-based (over-representation-based) GO enrichment analysis, which tests whether the list of genes with a specific GO term are significantly overlapped with the list of genes of interest using Fisher’s exact test. In our knowledge, this is a widely used method of GO enrichment analysis as described in Zhao and Rhee et al. (https://doi.org/10.1016/j.tig.2023.01.003). In Fig. 2E, GO term enrichment analysis was performed to check which GO terms are enriched in each gene list (ALL_high, Virus_high, TNK_low, etc.). In Fig. 4F and 4H, GO term enrichment analysis was performed on the list of DEGs in RaC5 and RaC7. Given the nature of GO term enrichment analysis, the issue of biological replicates is irrelevant because the number of genes (not the number of samples) is considered by Fisher’s exact test in this context.

Powered by Editorial Manager® and ProduXion Manager® from Aries Systems Corporation

Additional Information:	
**Question**	**Response**
Are you submitting this manuscript to a special series or article collection?	No
**Experimental design and statistics**<br><br>Full details of the experimental design and statistical methods used should be given in the Methods section, as detailed in our Minimum Standards Reporting Checklist. Information essential to interpreting the data presented should be made available in the figure legends.<br><br>Have you included all the information requested in your manuscript?	Yes
**Resources**<br><br>A description of all resources used, including antibodies, cell lines, animals and software tools, with enough information to allow them to be uniquely identified, should be included in the Methods section. Authors are strongly encouraged to cite Research Resource Identifiers (RRIDs) for antibodies, model organisms and tools, where possible.<br><br>Have you included the information requested as detailed in our Minimum Standards Reporting Checklist?	Yes
**Availability of data and materials**<br><br>All datasets and code on which the conclusions of the paper rely must be either included in your submission or deposited in publicly available repositories (where available and ethically appropriate), referencing such data using a unique identifier in the references and in the "Availability of Data and Materials" section of your manuscript.	Yes

Powered by Editorial Manager® and ProduXion Manager® from Aries Systems Corporation

Have you have met the above requirement as detailed in our Minimum Standards Reporting Checklist?	

Powered by Editorial Manager® and ProduXion Manager® from Aries Systems Corporation

Manuscript Click here to access/download;Manuscript;Manuscript_v3-final.docx

Single-cell transcriptome analysis illuminating the characteristics of species-specific innate immune responses against viral infections

Hirofumi Aso[1,2,3], Jumpei Ito[1], Haruka Ozaki[4,5], Yukie Kashima[6], Yutaka Suzuki[6], Yoshio Koyanagi[2,3], and Kei Sato[1,7,8,9,10,11,12,13,*]

1. Division of Systems Virology, Department of Microbiology and Immunology, The Institute of Medical Science, The University of Tokyo, Tokyo 1088639, Japan
2. Institute for Life and Medical Sciences, Kyoto University, Kyoto 6068507, Japan
3. Graduate School of Pharmaceutical Sciences, Kyoto University, Kyoto 6068501, Japan
4. Bioinformatics Laboratory, Faculty of Medicine, University of Tsukuba, Tsukuba 3050821, Japan
5. Center for Artificial Intelligence Research, University of Tsukuba, Tsukuba 3058577, Japan
6. Laboratory of Systems Genomics, Graduate School of Frontier Sciences, The University of Tokyo, Kashiwa 2778561, Japan
7. International Research Center for Infectious Diseases, The Institute of Medical Science, The University of Tokyo, Tokyo 1088639, Japan
8. International Vaccine Design Center, The Institute of Medical Science, The University of Tokyo, Tokyo 1088639, Japan
9. Graduate School of Medicine, The University of Tokyo, Tokyo 1130033, Japan
10. Graduate School of Frontier Sciences, The University of Tokyo, Kashiwa 2778561, Japan
11. Collaboration Unit for Infection, Joint Research Center for Human Retrovirus infection, Kumamoto University, Kumamoto, Japan
12. CREST, Japan Science and Technology Agency, Kawaguchi 3320012, Japan
13. Twitter: @SystemsVirology

*Correspondence address. Kei Sato, Division of Systems Virology, Department of Microbiology and Immunology, The Institute of Medical Science, The University of Tokyo, Tokyo 1088639, Japan. E-mail: keisato@g.ecc.u-tokyo.ac.jp

Hirofumi Aso [0000-0002-2826-3836];
Jumpei Ito [0000-0003-0440-8321];
Haruka Ozaki [0000-0002-1606-2762];
Yutaka Suzuki [0000-0003-4852-1879];
Yoshio Koyanagi [0000-0002-3007-6642];
Kei Sato [0000-0003-4431-1380]

Abstract

Background

Bats harbor various viruses without severe symptoms and act as their natural reservoirs. The tolerance of bats against viral infections is assumed to originate from the uniqueness of their immune system. However, how immune responses vary between primates and bats remains unclear. Here, we characterized differences in the immune responses by peripheral blood mononuclear cells to various pathogenic stimuli between primates (humans, chimpanzees, and macaques) and bats (Egyptian fruit bats) using single-cell RNA sequencing.

Results

We show that the induction patterns of key cytosolic DNA/RNA sensors and antiviral genes differed between primates and bats. A novel subset of monocytes induced by pathogenic stimuli specifically in bats was identified. Furthermore, bats robustly respond to DNA virus infection even though major DNA sensors are dampened in bats.

Conclusions

Overall, our data suggest that immune responses are substantially different between primates and bats, presumably underlying the difference in viral pathogenicity among the mammalian species tested.

Keywords: innate immunity; mammal; virus infection; single-cell RNA-sequencing; tensor

Background

Although a virus can infect various animal species, the pathogenicity of the infection can differ among host species. For example, Old World monkeys, including rhesus macaques (*Macaca mulatta*), are naturally infected with Cercopithecine herpesvirus 1 (also known as B virus) without any observable disorders, while humans (*Homo sapiens*) exhibit severe disorders after infection [1]. Bat species are naturally infected with a variety of viruses and behave as natural reservoirs of human pathogenic viruses [2]. For example, Marburg virus infection causes severe symptoms in humans but not in Egyptian fruit bats (*Rousettus aegyptiacus*), a putative natural host of this virus [3]. One possible factor that could define the differences in viral pathogenicity among host species is the difference in innate immune responses. For example, a previous study reported that Egyptian fruit bats lack the induction of proinflammatory cytokines, including *CCL8*, *FAS,* and *IL6*, which are related to disease severity in humans, upon Marburg virus infection, suggesting that the lack of cytokine induction is one of the reasons why Egyptian fruit bats exhibit asymptomatic infection with Marburg virus [4].

Pathogen sensing is the initial step in triggering innate immune signaling. In a broad range of animals, including vertebrates, pathogen-associated molecular patterns (PAMPs) are recognized by pattern recognition receptors (PRRs) to induce subsequent immune responses [5–8]. In humans and mice (*Mus musculus*), double-stranded RNAs (dsRNAs), a PAMP for RNA viruses, are recognized by RNA sensors, such as RIG-I, MDA5, LGP2, TLR3, and TLR7/8 [5, 6]. Extrachromosomal DNAs, a PAMP for DNA viruses, are recognized by cytosolic DNA sensors (e.g., cGAS, AIM2, and IFI16) and endosomal DNA sensors (e.g., TLR9) [5, 6, 9]. Lipopolysaccharide (LPS), a PAMP for bacteria, is recognized by TLR4 [5, 6, 10]. Once PAMPs are recognized by PRRs, type I interferons (IFNs) are produced, leading to the induction of IFN-stimulated genes (ISGs), which include many antiviral genes [5, 6].

In contrast to the similarities in the immune system between humans and mice, the immune system of bats is assumed to be quite different from that of humans in various aspects [11–13]. Genome analysis of Egyptian fruit bats showed expansion and diversification of immune-related genes, including type I IFN genes [14]. Transcriptome analysis showed that type I IFNs in the Australian black flying fox (*Pteropus alecto*) are constitutively expressed in unstimulated tissues, leading to the constitutive expression of ISGs [15]. These observations suggest that immunity in bats may be stronger than that in other mammals. In contrast, some studies have proposed

that immune responses in bats are dampened, resulting in bats exhibiting stronger tolerance to various viruses [12, 14, 16]. In particular, it is known that critical molecules involved in viral DNA sensing, such as cGAS, AIM2, and IFI16, are dampened or genetically lost in some bat species, including Egyptian fruit bats [16, 17]. These differences in innate immunity between humans and bats could be one of the reasons why viral pathogenicity differs between these two mammals.

Previous works have highlighted the uniqueness of the bat immune system using genomic analysis [14, 15, 17], transcriptome analysis [4, 18–20], and molecular biological experiments that reconstituted a part of the bat immune system in cell culture systems [16, 21, 22]. However, it remains unclear how and to what extent the innate immune response to pathogenic stimuli varies among mammals. Particularly, it is unclear how different innate immune responses are elicited by viral infections in different cell types in each mammal. Here, we used peripheral blood mononuclear cells (PBMCs) from four mammalian species including the above-mentioned Egyptian fruit bats and three pathogenic stimuli and conducted single-cell RNA sequencing (scRNA-seq) analysis to elucidate the differences in innate immune responses against pathogenic stimuli.

Results

Experimental design

To illuminate the differences in immune responses to infectious pathogens among mammalian species, we isolated PBMCs from four mammals including humans (*Homo sapiens*, Hs), chimpanzees (*Pan troglodytes*, Pt), rhesus macaques (*Macaca mulatta*, Mm), and Egyptian fruit bats (*Rousettus aegyptiacus*, Ra) (**Fig. 1A**). In this study, the Egyptian fruit bat was used as a representative model organism for bat species because this bat species is bred and available in captivity and is known to be a natural host of human pathogenic viruses, such as Marburg virus [3]. These PBMCs were inoculated with herpes simplex virus type 1 (HSV-1; a DNA virus), Sendai virus (SeV; an RNA virus), or lipopolysaccharide (LPS; a proxy for bacterial infection). We verified that these PBMCs could be infected with and/or respond to these viruses and LPS stimulation by quantifying viral RNAs and the upregulation of proinflammatory cytokines (e.g., IL1B and IL6), ISGs (e.g., EIF2AK2 and DDX58) and IFNB1 (**Fig. S1A–C**) at the level of mRNA transcripts.

To analyze immune responses to stimuli at single-cell resolution, we performed scRNA-seq analysis of 16 types of PBMC samples: four mammalian species (Hs, Pt, Mm, and Ra) versus four conditions (mock infection/stimulation, HSV-1 infection, SeV infection, and LPS stimulation) using the 10x Genomics Chromium platform at one day post-infection. Next, quality control (QC) was performed to exclude both cells with lower data quality and cells not targeted in this study (**Fig. S1D–G**) (See **Methods**). Before QC, there was a group of cells with low genes-per-cell and counts-per-cell in PBMCs of SeV-infected bats (**Fig. S1D–E**). Although one possible interpretation of this could be that SeV infection may have suppressed the gene expression in these cells, these cells were excluded to ensure the integrity of the downstream quantitative analysis. After filtering low-quality cells, a total of 40,717 cells from the 16 samples were used in the following analysis.

The cellular composition of PBMCs from primates and bats

We characterized the cellular composition of PBMCs from each mammalian species by annotating the cell type of individual single cells using tools available in Seurat [23, 24] and Azimuth [25] (See **Methods**). To establish a common classification system for the cells from the different mammalian species, we first identified cell types present in multiple species (**Fig. 1B and 1C**). As cell types detected in multiple species, naïve B cells, non-naïve B cells (including memory B cells and intermediate B cells), naïve CD4+ T cells, non-naïve CD4+ T cells (including central memory CD4+ T cells, effector memory CD4+ T cells, proliferating CD4+ T cells, and regulatory T cells), naïve CD8+ T cells, non-naïve CD8+ T cells (including central memory CD8+ T cells, effector memory CD8+ T cells, and proliferating CD8+ T cells), natural killer (NK) cells, mucosal-associated invariant T cells (MAITs), monocytes (Monos), conventional dendritic cells (cDCs), and plasmacytoid DCs (pDCs) were identified (**Fig. 1C**). Known marker genes for each cell type in humans were detected in the corresponding cell type in the unstimulated samples from the other animal species (**Fig. S2G**). Although most cell types were detected in all four species investigated, naïve CD8+ T cells and MAITs were undetectable in bat PBMCs, presumably because the cell numbers of these populations were relatively low in bats and/or the transcriptomic signatures of naïve CD4+ T cells and non-naïve CD8+ T cells were too similar in bats (hereafter we simply referred to Egyptian fruit bats as "bats") (**Fig. 1C**). This result was consistent with a previous study, in which clear clusters of naïve CD8+ T cells and MAITs were

not detected [26]. To establish a cellular classification system for the comparative transcriptome analysis, we defined six species-common cell types, namely, B cells, naïve T cells, killer TNK cells, Monos, cDCs, and pDCs, according to similarities in expression patterns (**Fig. S2H**).

The ratio of the six cell types exhibited different changes upon exposure to the stimuli in the different species (**Fig. 1D**). The frequency of monocytes decreased after stimulation in all four species, whereas the frequency of B cells and killer TNK cells changed differently within and across the animal species. Upon stimulation, there was generally a notable increase in B cells and a decrease of killer TNK cells in the bat (and non-human primates) samples, but not in the human samples.

Immune response differs largely among animal species

To describe the differences in immune responses to various stimuli in specific cell types among animal species, we first calculated the average expression levels of appropriate genes in each condition (4 animal species × 4 stimuli × 6 cell types = 96 conditions). Using this “pseudobulk” transcriptome dataset, we first investigated which axis (i.e., animal species, stimulus, and cell type) was the most impactful element in shaping the expression patterns of immune cells. Thereby, we calculated the fold-change (FC) values of gene expression levels between unstimulated and corresponding stimulated conditions and performed principal component analysis (PCA) on the FC values. Hierarchical clustering analysis was subsequently performed according to principal components (PCs) 1–30. The transcriptome data branch according to the animal species and then branch according to the cell type followed finally by the stimulus (**Fig. 1E**). This suggests the difference in host species is the more impactful element in shaping the immune system, having a greater impact than the type of stimulus and cell type. In particular, bat PBMCs exhibited different transcriptomic patterns irrespective of the type of stimulus and cell type compared to the PBMCs from the other three species used. These results suggest that bats respond to pathogens in a different manner than primates.

Extraction of species-specific immune responses

We next characterized the differences in the immune responses to pathogenic stimuli among animal species. The FC values of our pseudobulk transcriptome dataset were represented by a four-mode tensor (4 animal species × 3 stimuli × 6 cell types × 7557

orthologous genes). To characterize this extraordinary high-dimensionality transcriptome dataset, we utilized Tucker decomposition, a method of tensor decomposition (**Fig. 2A**). In this analysis, we excluded cDC and pDC data due to many missing values. Tucker decomposition generated a core tensor and four-factor matrices (A1–A4) related to the four axes (animal species, stimulus, cell type, and gene). For example, the factor matrix A1 (for host species) included three latent factors (L1_1, L1_2, and L1_3), which could be regarded to represent common, bat-specific, and macaque-specific expression patterns, respectively (**Fig. 2B**).

To characterize species-specific immune responses, we developed a gene classification system according to the pattern of the species-associated latent factor in the tensor decomposition framework. First, we calculated the product of a core tensor and the three-factor matrices A2 (for stimulus), A3 (for cell type), and A4 (for gene) (**Fig. 2C and Fig. S3A–B**). Consequently, we obtained three cubic datasets with three axes—stimulus, cell type, and gene. These cubic data were related to L1_1 (for the common factor), L1_2 (for the bat-specific factor), or L1_3 (for the macaque-specific factor). Subsequently, we classified the genes into 10 categories according to their expression patterns in each cubic dataset (the results for the bat-specific (L1_2) and other factors (L1_1 and L1_3) are shown in **Fig. 2D, Fig. S3G, and Fig. S3I**, respectively). In the factor matrix A2 (for stimulus), the values for the latent factors related to HSV-1 and SeV were similar (**Fig. S3A**). Therefore, these two categories were integrated into the category "Virus" in the gene classification. Additionally, two cell type categories, NaiveT and KillerTNK, were integrated into the category "TNK" (**Fig. S3B**). The pattern for raw FC values supported that the gene classification by the tensor decomposition framework succeeded in extracting the characteristic patterns of gene expression alterations upon pathogenic stimuli (**Fig. S3J–L**).

Differential dynamics of pathogen sensing and immune responses

To highlight the uniqueness of immunity in bats compared to that in primates, we focused on the expression pattern represented by the bat-specific factor (L1_2) and performed Gene Ontology (GO) analysis on the 10 gene categories (**Fig. 2E**). In the gene category "ALL_high", which included genes upregulated particularly in bats regardless of the stimulus and cell type, GO terms related to innate immune responses, such as IFN signaling, DDX58/IFIH1-mediated induction of IFN, RIG-I like receptors

(RLRs) signaling pathways, and the antiviral mechanism by ISGs, were over-represented.

To dissect the "ALL_high" genes in the bat-specific factor, we further extracted the genes that belonged not only to the "ALL_high" category in the bat-specific factor but also to that in the common factor (L1_1). This fraction represented genes that were upregulated by stimuli in all species but whose induction levels were highest in bats. These genes included various PPRs, such as RIG-I-like receptors (RLRs) (RIG-I, LGP2, and MDA5) and cGAS, a DNA sensor, suggesting that these genes were upregulated to higher levels in bats than in the other species across the cell types and stimuli (**Fig. 2F**). There are two possible scenarios that could potentially explain these higher FC values observed in bats. One possibility is that expression levels of these genes after stimulation are higher than in primates. The second possibility is that basal expression levels of these genes in bats are lower than those in primates. Therefore, we calculated the relative expression levels of these genes in bats compared to humans and showed that the basal expression levels of these genes were lower in bats than in humans (**Fig. 2G**). These results suggest that the induction dynamics of these PRRs in bats are likely different from those in primates, possibly leading to the differences in the induction of immune responses.

Robust immune responses to a DNA virus in bats

As critical DNA sensors, such as cGAS, AIM2, IFI16, and TLR9, are dampened or genetically lost in bat species [16, 17, 27], it has been hypothesized that bats, including Egyptian fruit bats, cannot efficiently activate innate immune responses against DNA viruses. To test this hypothesis, we analyzed the IFN response upon HSV-1 (a DNA virus) infection. However, the expression levels of IFN-α genes were not examined because they were not annotated in the transcript model for the Egyptian fruit bat used in this study, and the expression of IFN-β genes were too low. Thus, even though the expression levels of IFN-I is the primary factor to examine the activity of the IFN response, we instead analyzed the induced levels of "core^{mamm} ISGs"—a set of genes that are commonly induced by type I IFNs across mammals that were defined in a previous study [28]. Intriguingly, we found that the core^{mamm} ISGs were upregulated upon HSV-1 infection in most cell types in bats (**Fig. 3A**). The induced levels were comparable to those induced by SeV (an RNA virus) infection and higher than those induced by LPS stimulation. Furthermore, the induced levels in bats were comparable

to those in primates. This suggests that immune cells in bats can sense and respond to HSV-1 infection even though critical DNA sensors are dampened.

To address the possibility that pathogen sensors other than DNA sensors contribute to the sensing of HSV-1 infection in bats, we examined the expression levels of various PRRs (**Fig. 3B**). The expression of some PRRs, including TLR3, a dsRNA sensor associated with HSV-1 sensing in humans and mice [29], was detected not only in primates but also in bats, suggesting the possibility that these PRRs compensate in the response to HSV-1 infection in bats (see **Discussion**).

Identification of bat-specific subsets of monocytes

Next, we investigated cellular subsets within the cell types that are characteristic in bats to explain the differences in immune responses among the species. We particularly searched for cellular subsets that specifically appeared after pathogenic stimulus exposure in each species according to the dimensionality reduction analysis of transcriptome data. In humans, chimpanzees, and macaques, no subset appeared in any cell type after stimulation (**Fig. S4A**). Similarly, such subsets were not identified in T/NK or B cells in bats. In contrast, we found that two subsets of bat monocytes (referred to as Clusters 5 and 7) specifically appeared after stimulation (**Fig. 4A**). To validate whether these subsets (Clusters 5 and 7) are unique in bats, we identified marker genes for these clusters and subsequently examined whether the marker genes were expressed in monocytes from the other animal species. The marker genes for Cluster 5 (referred to as C5 markers) were not highly expressed in any cluster of monocytes from primates (**Fig. 4B**). Furthermore, high expression levels of C5 markers in bat monocytes were found only after stimulation. This suggested that Cluster 5 was not only bat-specific but also specifically induced by pathogenic stimuli. Unlike the C5 markers, the marker genes for Cluster 7 (C7 markers) were highly expressed not only in bat Cluster 7 but also in some monocytes in primates (**Fig. 4C**). Although cells with higher expression of C7 markers were induced upon stimulation in both bats and primates, these cells in primates did not form a separate cluster similar to Cluster 7 in bats (**Fig. S4B**). Furthermore, the proportions of Clusters 5 and 7 differed depending on the stimulus: HSV-1-infected and LPS-stimulated samples showed the highest frequencies of Clusters 5 and 7, respectively (**Fig. 4D**).

To characterize these two clusters, we identified differentially expressed genes (DEGs) in Clusters 5 and 7 compared to the other clusters of bat monocytes. According to GO analysis, Cluster 5 is characterized by lower expression of ISGs (**Fig. 4E, 4F**). Additionally, Cluster 5 highly expresses known suppressors of the inflammatory response, such as DUSP1, DUSP5, and SOCS2 [30–32]. On the other hand, Cluster 7 can be characterized by a higher expression of various cytokines related to chemotaxis (**Fig. 4G**), including CXCL6, IL18BP, CXCL8, CCL2, CCL8, CCL13, CCL5, CXCL10, IL15, and IL4I1 (MSigDB [33]: GO:0060326) (**Fig. 4G, 4H**). Overall, we established that there are two unique subsets of bat monocytes with different characteristics (see **Discussion**).

Discussion

Differences in viral pathogenicity among host species are thought to be attributed to differences in immune responses against viral infections among the species [34]. However, it remains unclear how immune responses, particularly innate immunity against viral infections, differ among host species. In the present study, we performed scRNA-seq on 16 types of PBMC samples, derived from a combination of four host species and four infection conditions (**Fig. 1A**), and showed that the differences in the immune responses among the host species were more impactful than those among both the stimuli and the cell types (**Fig. 1E**). In particular, the transcriptomic changes resulting from pathogenic stimulation in bats differed from those in primates. It is also noteworthy that post-stimuli changes in the ratio of cell types differed between humans and bats (**Fig. 1D**). For further analysis, we established a bioinformatic pipeline to characterize species-specific immune responses from transcriptome profiles with extraordinarily high dimensions (4 animal species × 3 stimuli × 4 cell types × 7,557 orthologous genes) (**Fig. 2A**). We illuminate differences in innate immune systems among mammalian species that partly explain the differences in viral pathogenicity among host species.

It is known that two DNA sensing pathways mediated by cGAS-STING pathway [16] and PYHIN proteins, including AIM2 and IFI16 [17], are dampened in bats, including Egyptian fruit bats. In addition, a previous study using a cell line derived from big brown bats (*Eptesicus fuscus*) suggested that the TLR9-mediated DNA sensing pathway is also weakened in bats [27]. Based on these observations, it was hypothesized that the ability to sense DNA virus infection is weakened in bats [12, 13]. However, we showed that bat PBMCs robustly induced IFN responses upon infection with the DNA virus HSV-1 (**Fig. 3A**). This suggests that bats can initiate an innate immune response after infection with DNA viruses (at least HSV-1) and that bats have another pathway to sense DNA viruses. An alternative possibility is that the IFN response in response to HSV-1 infection was triggered by sensing viral molecules other than DNAs. It is known that, in humans and mice, dsRNA sensing by TLR3 plays an important role in responding to HSV-1 infection [29, 35]. Additionally, the Egyptian fruit bat genome encodes an intact TLR3 gene (NCBI Gene ID: 107510436), and bat immune cells express TLR3 (**Fig. 3B**). Furthermore, other RNA sensors, such as RIG-I, LGP2, and MDA5, were upregulated in bat cells similarly as in primate cells upon HSV-1 infection (**Fig. 3B**). These data suggest that TLR3 or other RNA sensors in bats

may compensate for weakened DNA sensing pathways, leading to IFN responses to HSV-1 infection.

To characterize the bat-specific innate immune responses based on ultrahigh-dimensionality transcriptome data (4 animal species × 4 stimuli × 6 cell types × 7,557 orthologous genes), we established an analytical framework utilizing tensor deconvolution (**Fig. 2A**). This framework could i) extract a species-specific effect on gene expression changes, ii) compare the effects among the cell types and the stimuli, and iii) classify genes according to the differential pattern of a species-specific effect among the cell types and the stimuli. Using this framework, we found that the expression levels of key DNA and RNA sensors, including cGAS, RIG-I, MDA5, and LGP2, were highly induced in bats compared with primates, regardless of the cell type or stimulus (**Fig. 2F**). Furthermore, the basal expression levels of these PRRs in bats were lower than those in humans (**Fig. 2G**). On the other hand, after stimulation, the expression levels of these PRRs in bats were comparable to those in humans. These results suggest that the induction dynamics of these PRRs in bats are likely different from those in primates, leading to the differences in the induction of immune responses. Indeed, several antiviral ISGs, such as IFI6 and IFIT3, exhibited expression dynamics similar to those of these PRRs (**Fig. 2F, 2G**). These differences could be one of the reasons why immune responses differ between bats and primates.

Another factor that can explain the differences in immune responses among host species is the presence of species-specific cellular subsets. In bat monocytes, we identified two subsets that were specifically induced by stimuli (i.e., Clusters 5 and 7) (**Fig. 4A**). Cluster 5 was a bat-specific subset induced preferentially by HSV-1 infection (**Fig. 4B, 4D**). Interestingly, even though Cluster 5 was induced after stimulation, Cluster 5 exhibited lower expression of ISGs and higher expression of immunosuppressive genes (DUSP1, DUSP5, and SOCS2) [30–32] (**Fig. 4E, 4F**). This observation suggests that the immune responses in Cluster 5 are downregulated presumably by negative feedback signaling and that Cluster 5 may contribute to controlling excessive immune activation in bats. On the other hand, Cluster 7 was identified as a monocyte subset that was mainly induced by LPS stimulation (**Fig. 4C, 4D**). Cluster 7 highly expressed several proinflammatory cytokines and chemokines (CXCL6, IL18BP, CXCL8, CCL2, CCL8, CCL13, CCL5, CXCL10, IL15, and IL4I1) (**Fig. 4G, 4H**). Cluster 7 may contribute to the recruitment of leukocytes since these cytokines are associated with the chemotaxis of neutrophils (CCL8, CXCL6, and

CXCL8), basophils (CXCL8, CCL2, CCL5, CCL8, and CCL13), eosinophils (CCL5, CCL8, and CCL13), monocytes (CCL5, CCL8, and CCL13), T cells (CCL5, CCL8, CCL13, CXCL8, and CXCL10), and NK cells (CCL5 and CCL8) in humans and mice [36, 37]. Based on the expression pattern of the marker genes for Cluster 7 (**Fig. 4C, S4B**), cellular subsets corresponding to Cluster 7 were also present in primate monocytes. However, these primate cells did not form a separate cluster in the dimensionality reduction analysis based on the transcriptome profile (**Fig. 4A**). These results suggest that the monocyte subset represented by Cluster 7 exhibits unique gene expression and thus may exert unique functions in bats. Although the specific functions of these monocyte subsets (Clusters 5 and 7) in immune responses in bats are still unclear, these unique subsets may contribute to bat-specific host immune responses.

Limitations of the study

In the present study, we elucidated differences in innate immune responses among host species from various aspects. However, we did not address differences in the outcomes of the innate immune responses, such as differences in viral pathogenicity. Another limitation is that the bioinformatic resources we used, such as gene annotation, gene ontology, and cellular annotation, have been developed in a human-centric way. Therefore, there is the possibility that immune responses induced by species-specific genes and cell types were overlooked. Moreover, because the results of this study rely on an analysis using a single bat species, the Egyptian fruit bat, it is unclear whether the observed bat-specific characteristics are conserved across bat species. Furthermore, we did not perform biological replicates of scRNA-seq in this study. Despite these limitations, we present valuable resources to illuminate differences in immune responses among host species, including Egyptian fruit bats, and clues to elucidate differences in viral pathogenicity among species. Further study to elucidate the functional consequences of these differences is needed to reveal the mechanisms by which bats can tolerate infections with various viruses.

Figure legends

Figure 1. scRNA-seq analysis of PBMCs from four animal species inoculated with pathogenic stimuli

(A) Schematic of the experimental design. See also **Fig. S1**.

(B) Uniform manifold approximation and projection (UMAP) plots representing the gene expression patterns of the cells from the four species. Each dot is colored according to the cell type. Gray dots indicate cells unassigned into any cell type. See also **Fig. S2**.

(C) Comparison of identified cell types among the species. Dot: detected, question mark: undetected. The definitions of six species-common cell types are shown on the right side. See also **Fig. S2H**.

(D) The cellular compositions of PBMC samples. The compositions according to the six common cell types are shown.

(E) Hierarchical clustering analysis of 48 pseudobulk datapoints (4 animal species x 3 stimuli x 4 cell types = 48 conditions) based on PC1-30 calculated from the fold-change values (respective stimulus versus unstimulated) for gene expression.

Figure 2. Characterization of species-specific immune responses using a tensor decomposition framework

(A) Tensor decomposition of the fold-change values for pseudobulk transcriptome data.

(B) Heatmap representing a latent factor matrix relating to species. Columns indicate the animal species, and rows indicate the latent factors representing species-common (L1_1), bat-specific (L1_2), and macaque-specific (L1_3) factors. See also **Fig. S3A–B**.

(C) Classification of genes according to the differential patterns of the latent factors related to species. For each of the species-common (L1_1), bat-specific (L1_2), and macaque-specific (L1_3) factors, the product of the core tensor and three latent factor matrices related to stimulus, cell type, and gene was calculated (left), and the genes were classified into 11 categories according to the binary patterns for each calculated product (right). See also **Fig. S3C–F**.

(D) Heatmap representing the values of the products calculated in **Figure 2C**. From the three products, the data related to the bat-specific factor (L1_2) are shown. Each

row indicates the respective gene. The color keys shown on the right of the heatmap indicate gene categories. See also **Fig. S3G–L**.

(E) GO terms enriched in each gene category relating to the bat-specific factor. GO terms with a false discovery rate (FDR) <= 0.1 and an odds ratio >= 1 are shown.

(F) Heatmap representing the induction levels of ALL_high genes for the bat-specific factor. Additional classification according to the gene classification of the species-common factors is shown to the right of the heatmap. Genes categorized as ALL_high in both the species-common factor and the bat-specific factor are shown on the right side. The colored circle indicates the functional category of the gene.

(G) Heatmap representing the relative expression levels (bats versus humans) of the genes shown in **Figure 2F**.

Figure 3. Robust immune responses to a DNA virus in bats

(A) Boxplot of the expression levels of core^{mamm} ISGs in every single cell. The Y-axis indicates the global expression level (GSVA score) of the core^{mamm} ISGs.

(B) Heatmap representing the mean expression levels of sensor genes. The mean values were calculated without using the information for the stimulus.

Figure 4. Identification of bat-specific subsets of monocytes

(A) UMAP plots representing the gene expression patterns of monocytes from the four species. The dots are colored according to the cell cluster defined for each animal species. See also **Fig. S4A**.

(B, C) UMAP plots representing the average expression levels of marker genes for Cluster 5 [C5markers] (B) and Cluster 7 [C7markers] (C). See also **Fig. S4B**.

(D) The cellular composition of bat monocytes. The composition is shown according to the cluster. The black frame indicates Clusters 5 and 7 in stimulated samples.

(E) Heatmap representing the mean expression levels of differentially expressed genes (DEGs) in Cluster 5 of bat monocytes.

(F) Summary of the GO terms enriched in DEGs in Cluster 5. GO terms enriched in up- and downregulated genes are shown in red and blue, respectively.

(G) Heatmap representing the mean expression levels of differentially expressed genes (DEGs) in Cluster 7 of bat monocytes.

(H) Summary of the GO terms enriched in DEGs in Cluster 7. GO terms enriched in up- and downregulated genes are shown in red and blue, respectively.

Methods

Cells

Vero cells (obtained from the Laboratory of Bernard Roizman, University of Chicago, USA)

LLC-MK2 cells (rhesus macaque kidney epithelial cells) (CCL-7, ATCC)

PBMC collection

Human peripheral blood was obtained from the arm vein. To obtain chimpanzee peripheral blood, a chimpanzee was anesthetized for a regular health examination. Anesthesia was induced with intramuscular administration of the combination of 0.012 mg/kg medetomidine (Meiji Seika Pharma Co., Ltd.), 0.12 mg/kg midazolam (Sand Co., Ltd.), and 3.5 mg/kg ketamine (Fujita Pharm, Tokyo) and maintained with constant rate infusion (4-10 mg/kg/h) of propofol (1% Diprivan, Sand Co., Ltd.). Peripheral blood was obtained from the femoral vein. To obtain rhesus macaque peripheral blood, a rhesus macaque was anesthetized. Anesthesia was induced with intramuscular administration of 8 mg/kg ketamine followed by deep anesthetization using an intravenous injection of sodium pentobarbital (30 mg/kg) (Kyoritsu Seiyaku). Peripheral blood was obtained by cardiac puncture before exsanguination and perfusion. Bat peripheral blood was obtained from the cephalic vein in the patagium. PBMCs were isolated from peripheral blood by density gradient centrifugation using Ficoll-Paque™ Plus (Cytiva, Cat# 17144003).

HSV-1 preparation and titration

HSV-1 (strain F; GenBank accession number: GU734771) [38] was prepared as previously described [29] and kindly provided by Dr. Yasushi Kawaguchi (The Institute of Medical Science, The University of Toyo, Japan). Briefly, Vero cells were infected with HSV-1 and the supernatant was collected and used without purification. To titrate viral infectivity, prepared virus was diluted 10-fold in Medium 199 (Thermo Fisher Scientific, Cat# 11825015) containing 1% fetal calf serum (FCS) (Nichirei Biosciences, Cat# 175012), and Vero cells were infected with dilutions of the virus at 37 °C. At one hour postinfection, the culture medium was replaced with Medium 199 containing 160 µg/ml human γ-globulin (Sigma Aldrich, G4386-25G), and the cells were cultured at 37 °C for 2–3 days. To calculate the viral titer [plaque forming unit (PFU)], the number of plaques per well was counted.

SeV preparation and titration

SeV (strain Cantrell, clone cCdi; GenBank accession number: AB855654) was prepared as previously described [39] and kindly provided by Dr. Takashi Irie (Hiroshima University, Japan). Briefly, LLC-MK2 cells were infected with SeV and the supernatant was collected and used without purification. To titrate viral infectivity, prepared virus was diluted 10-fold in Dulbecco's modified Eagle's medium (DMEM) (Sigma–Aldrich, Cat# D6046-500ML) containing 10% FCS, and LLC-MK2 cells were infected with dilutions of the virus at 37 °C. At one hour postinfection, the cells were washed with PBS and cultured with DMEM containing 10% FCS at 37 °C. At one day postinfection, the infected cells were fixed with acetone (Nacalai Tesque, Cat# 21914-03)/methanol (Nacalai Tesque, Cat# 00310-95). To calculate the viral titer [cell infectious unit (CIU)], the fixed cells were stained with a rabbit anti-SeV polyclonal antibody [40] as the primary antibody and an Alexa 488-conjugated goat anti-rabbit IgG antibody (Thermo Fisher Scientific, Cat# A-11008) as the secondary antibody, and the number of fluorescent foci per well was counted.

Infection and stimulation

One million PBMCs were maintained in 500 μl RPMI 1640 medium (Sigma–Aldrich, Cat# R8758-500ML) and infected with HSV-1 or SeV at a multiplicity of infection of 0.1. To mimic microbial infection, LPS (Sigma–Aldrich, Cat# L5024-10MG) was added at a final concentration of 200 ng/ml. At one day post infection, all types of infected/stimulated PBMCs were centrifuged, resuspended in PBS, and used for bulk RT–qPCR and scRNA-seq (see below).

RT–qPCR

RT–qPCR was performed as previously described [41]. Briefly, cellular RNA was extracted using the QIAamp RNA Blood Mini Kit (Qiagen, Cat# 52304) and then treated with an RNase-free DNase set (Qiagen, Cat# 79254). cDNA was synthesized using SuperScript III reverse transcriptase (Thermo Fisher Scientific, Cat# 18080044) and random primers (Thermo Fisher Scientific, Cat# 48190011). RT–qPCR was performed using Power SYBR Green PCR Master Mix (Thermo Fisher Scientific, Cat# 4367659) and the primers listed in **Table S1**. For RT–qPCR, the CFX Connect Real-Time PCR Detection System (Bio-Rad) was used.

Sequencing of scRNA-seq libraries

scRNA-seq libraries were constructed using the Chromium Next GEM Single Cell 3' Kit according to the manufacturer's instructions (10x Genomics). Briefly, cells, gel beads, and oil were loaded onto the Chromium platform to generate single-cell gel beads-in-emulsion (GEMs). Before loading, cell numbers and viability were confirmed. To acquire 5,000 cells recovery, 8,000 cells were loaded. Barcoded cDNAs were pooled for amplification, and adaptors and indices for sequencing were added. The evaluation was conducted using a BioAnalyzer (Agilent Technologies). The libraries were sequenced with paired-end reads using the Illumina NovaSeq6000 platform (RRID:SCR_016387) .

Genome sequence dataset

Genome sequences of the animal species including humans (GRCh38.p13, RefSeq accession: GCF_000001405.39), chimpanzees (Clint_PTRv2, RefSeq accession: GCF_002880755.1), rhesus macaques (Mmul_10, RefSeq accession: GCF_003339765.1), and Egyptian fruit bats (mRouAeg1.p, RefSeq accession: GCF_014176215.1) were obtained from NCBI RefSeq [42]. From the genome sequences, ALT contig sequences were excluded. The genome sequences of viruses including HSV-1 (strain: F, accession: GU734771.1) and SeV (strain: Cantell clone cCdi, accession: AB855654.1) were also obtained from NCBI RefSeq. A custom reference genome sequence for each animal species was generated by adding the genome sequences of HSV-1 and SeV to the genome sequence of the animal species.

Gene annotation and ortholog information

Gene annotations of humans (GRCh38.p13, Release 109.20200228), chimpanzees (Clint_PTRv2, Release 105), rhesus macaques (Mmul_10, Release 103), and Egyptian fruit bats (mRouAeg1.p, Release 101) were obtained from NCBI RefSeq. From the gene annotations, only the records for protein_coding, transcribed_pseudogene, lncRNA, pseudogene, antisense_RNA, ncRNA_pseudogene, V_segment, V_segment_pseudogene, C_region, C_region_pseudogene, J_segment, J_segment_pseudogene, and D_segment were extracted according to the CellRanger tutorial [43]. In addition, to quantify viral RNA abundance, the records for viruses were added. The whole viral genome was treated

as a single exon, and a total of four lines (the positive and negative strands of HSV-1 and SeV) were added.

A list of orthologous genes between humans and the other animal species (chimpanzees, rhesus macaques, and Egyptian fruit bats) was obtained from NCBI on July 26th, 2021 [44]. From the file, the records for orthologs between humans (taxonomy ID: 9606) and chimpanzees (taxonomy ID: 9598), rhesus macaques (taxonomy ID: 9544), or Egyptian fruit bats (taxonomy ID: 9407) were extracted.

The ortholog list from NCBI lacked information on some critical immune-related genes of Egyptian fruit bats, such as CD4 and IRF1. Therefore, we retrieved information from the Bat1K gene annotation [45, 46] downloaded from UCSC genome browser [47]: First, we made a custom gene annotation for Egyptian fruit bats by adding information from the Bat1K gene annotation to the RefSeq gene annotation. Second, we extracted exons in the Bat1K gene annotation that overlapped with exons in the RefSeq gene annotation by using the bedtools intersect command with the wao option (v2.30.0) [48]. In this step, the exons in the Bat1K gene annotation that did not overlap with the exons in the RefSeq gene annotation were also extracted and added to custom gene annotations as additional genes. Next, the exons that contained overlaps and had the same gene name (the same symbol or known to be an ortholog) were added to custom gene annotations as an alternative splicing variant of the gene. Then, the remaining overlapping exons were processed by determining which information (RefSeq or Bat1K) should be used preferentially. The criteria were as follows: i) genes whose symbols are not prefixed with “LOC” were given priority, ii) genes whose symbols are included in the human gene list were given priority, and iii) information from RefSeq was given priority otherwise. According to these criteria, the annotation with the higher priority (RefSeq or Bat1K) was selected and used in the custom gene annotation.

As a result of the integration of gene annotations, the number of orthologous genes in the custom gene annotation of bats increased from 16374 to 16903. Importantly, immune-related genes that were not defined in the RefSeq gene annotation, such as TLR1, IRF1, and CD4, were added to the custom gene annotation.

Considering the orthologous relationships, we prepared three types of gene sets for each animal species: i) “all genes”, including all genes in the animal species; ii) “genes shared with humans”, including genes with orthologs in humans; and iii) “common genes”, genes shared among the four analyzed animal species. Unless

otherwise noted, “all genes” were used up to cell annotation, and “common genes” were used after cell annotation.

Processing scRNA-seq data for generating count matrices

Gene expression count matrices for scRNA-Seq data were generated using CellRanger (RRID:SCR_023221) (v6.0.1) (10x Genomics) [49, 50]. First, we built a custom reference for each animal species from the custom reference genome sequence and custom gene annotation using the “cellranger mkref” command. Subsequently, we generated unique molecular identifier (UMI)-based count matrices from the raw scRNA-seq data and custom references using the “cellranger count” command with default settings.

Quality control (QC) of scRNA-seq data

First, we removed cells with abnormal genes per cell (genes/cell) and counts per cell (counts/cell) values using the Seurat package (RRID:SCR_016341) (v4.0.4) [23, 24]: Cells with 800–5,000 genes/cell or 1,200–25,000 counts/cell were extracted. The thresholds were determined based on the distributions of genes/cell and counts/cell before QC (**Fig. S1D–E**). Second, we annotated the cell type of individual cells using Azimuth (v0.4.3) [25], a reference-based cell annotation prediction program, and then, cells annotated as erythrocytes, platelets, hematopoietic stem cells, or innate lymphoid cells were excluded as nontargeted cells in the present study. This is because erythrocytes and platelets are probably residuals after experimental PBMC extraction, and hematopoietic stem cells and innate lymphoid cells are not the major cell types in the analysis of innate immune responses using PBMCs. In this step, the gene annotation “genes shared with humans” (see **Gene annotation and ortholog information**) for each animal species was used. Finally, regarding genes/cell and counts/cell values, cells with >3 |Z score| were excluded as outliers.

Data integration, visualization, and cell clustering

Data integration, visualization, and cell clustering for each animal species were performed using the Seurat package. In these processes, the expression levels of HSV-1 and SeV were not used.

Data integration is a method merging the gene expression count matrices obtained from different experimental conditions while removing batch effects. We

integrated the count matrices from the four different conditions for each animal species. In the data integration, SCTransform (RRID:SCR_022146) (a modeling framework for the normalization and variance stabilization of molecular count data from scRNA-seq data) was performed using the SCTransform function for each count matrix. Next, to extract 2000 genes with higher variance and thus greater information for integration, the four count matrices were processed using the SelectIntegrationFeatures function. Next, we used the PrepSCTIntegration function to transform normalized counts into counts per 10,000 counts in the cell (CP10k). After that, we used the FindIntegrationAnchors function with the setting Mock as a reference to find “Integration anchors”. Finally, we integrated the four normalized count matrices using the IntegrateData function with the option ‘normalization.method=”SCT”’.

For visualization, we first performed principal component analysis (PCA) using the RunPCA function. Then, UMAP (RRID:SCR_018217) [51] was performed with the RunUMAP function. In this step, principal components (PC) 1-50 were used, and the parameter “n.neighbors” was set individually for each animal species (Hs: 20, Pt: 20, Mm: 50, and Ra: 40).

To define cell clusters in each animal species, we performed graph-based unsupervised clustering (**Fig. S2A**). First, the FindNeighbors function was used, and then, the FindClusters function was used. In these steps, the parameter ‘k.param’ for FindNeghbors was set individually for each animal species (Hs: 12, Pt: 10, Mm: 10, and Ra: 20). The parameter ‘resolution’ for FindClusters was also set individually for each animal species (Hs: 2.0, Pt: 2.2, Mm: 1.7, Ra: 1.2).

Cell annotation

Regarding each cluster identified by graph-based unsupervised clustering in the section “**Data integration, visualization, and cell clustering**” (**Fig. S2A**), 11 cell types were manually annotated according to i) the predicted cell type by Azimuth (**Fig. S2B**), ii) the distances between each cluster (**Fig. S2C**), and iii) the correspondence of clusters between animal species (**Fig. S2D–F**). First, reference-based cell type prediction was performed using Azimuth for the mock data from each animal species (**Fig. S2B**). In this step, the gene annotation “genes shared with humans” (see **Gene annotation and ortholog information**) for each animal species was used. We checked the enrichment of each predicted cell type in each cluster by Azimuth. Second, we checked the similarities between clusters by hierarchical clustering (**Fig. S2C**)

using the mean values of PCs 1-50 among the individual cells (see **Data integration, visualization, and cell clustering**) in each cluster. Notably, PCA was performed using the expression levels of “all genes” (see **Gene annotation and ortholog information**). The Euclidian distance was used for clustering by Ward’s method. Third, to check the correspondence between clusters in each animal species, we performed data integration, clustering, and visualization for mock data from all four animal species (**Fig. S2D–F**). In the integration, the mock data from humans were used as reference data. In this step, the gene annotation “common genes” (see **Gene annotation and ortholog information**) was used.

After categorizing cells into 11 cell types, the 11 cell types were coarse-grained into 6 cell types based on the results of hierarchical clustering analysis (see **Hierarchical clustering**). The six cell types were used in the subsequent analysis.

Hierarchical clustering

To examine the similarities in expression patterns among the conditions (4 animal species × 4 stimuli × 6 cell types = 96 conditions), hierarchical clustering analysis was performed. In this analysis, the 5,000 genes with the highest median absolute deviation (mad) values were used (**Fig. S2H**). First, the average expression levels of the respective genes in each condition were calculated. Next, PCA was performed using the average expression profiles. Third, using PCs 1-30, the distance matrix for the 96 conditions was generated using 1−Pearson’s correlation coefficient. Finally, hierarchical clustering by Ward’s method was performed using the distance matrix.

To determine which factor (e.g., animal species, stimulus, or cell type) was the most impactful on the gene expression in immune cells, hierarchical clustering was performed using induction patterns upon stimulation (**Fig. 1E**). Unlike for the results shown in **Fig. S2H**, FC values were used to perform PCA. This analysis used 7557 genes, the union of the top 6000 genes related to total expression levels in the expression profiles of each animal species. The FC expression values (stimulated vs. unstimulated conditions) of those genes were calculated for each cell type in each animal species. To avoid generating infinite FC values, the data for genes with zero expression in mock data were set at the minimum nonzero expression level in the mock data. Finally, hierarchical clustering was performed using the method described above.

Tensor decomposition

To extract species-specific/common induction patterns upon stimulation from transcriptome data with complex structures (4 animal species × 3 stimuli × 4 cell types × 7557 orthologous genes), we used tensor decomposition (**Fig. 2A**). As the input data for tensor decomposition, the FC values of 7557 genes, the union of the top 6000 genes related to total expression levels in the expression profiles of each animal, were used. The calculation method for FC values is described in the section "**Hierarchical clustering**". The standardized FC values for each condition were represented as a 4-mode tensor (animal species × stimulus × cell type × orthologous gene). To perform Tucker decomposition (TD), a method of tensor decomposition, we used TensorLy (v0.6.0) [52]. We performed TD via higher-order orthogonal iteration (HOI) with the parameter 'init="svd"'. In HOI, the size of the core tensor (ranks) was set as [animal species: 3, stimulus: 2, cell type: 3, gene: 15]. The number of iterations was set as 100.

Gene classification using the tensor decomposition results

A schematic of the gene classification using tensor decomposition is shown in **Fig. 2C and Fig. S3C–F**. Briefly, we selected the candidate gene categories that had patterns of values (high, mid, or low) (**Fig. S3C**) that matched the ideal pattern (**Fig. S3D**) and then selected the gene category with the best "similarity score" (**Fig. S3E**) from the candidates as the gene category for that gene (**Fig. S3F**).

Initially, the product of the core tensor and the three factor-matrices, A2 (for stimulus), A3 (for cell type), and A4 (for gene), was calculated to obtain three cubic data with three axes, stimulus, cell type, and gene, using the ttl function of rTensor (v1.4.8) [53]. Each cubic data point indicated information related to species-common, bat-specific, and macaque-specific factors (**Fig. 2B**). Next, since the values of latent factors related to HSV-1 and SeV were similar (**Fig. S3A**), these two categories were integrated into the category "Virus" by calculating mean values. Additionally, since the values of latent factors related to NaiveT and KillerTNK were similar (**Fig. S3B**), these two categories of cell types were integrated into the category "TNK" by calculating mean values. Thus, hereafter, the category of stimuli included virus and LPS, and the category of cell types included B cells, TNK cells and Monos.

Then, in each cubic data, genes were classified into 11 categories (**Fig. 2C**) through the following three steps. Briefly, from the candidate gene categories that had patterns of values (high, mid, or low) (**Fig. S3C**) that matched the ideal pattern (**Fig. S3D**), the gene category with the lowest “similarity score” (**Fig. S3E**) was selected as the gene category for that gene (**Fig. S3F**).

In the first step (**Fig. S3C**), the values in each cubic data were normalized, and the genes were classified into three classes (high, mid, and low) according to the ranking of values in each condition (stimulus × cell type). First, six column vectors in the TD results for the 6 conditions (2 stimuli × 3 cell types) were normalized by dividing them by the 90th percentile for the individual vectors. After the division step, to suppress the effect of abnormally high or low values, data with > 1 or < -1 were assigned as 1 and -1, respectively. Next, the genes were categorized into three classes based on the rule that if the rank of a value was greater than the 80th percentile or smaller than the 20th percentile, it was categorized as “high” or “low”, respectively; otherwise, it was categorized as “mid”.

In the second step (**Fig. S3E**), a “similarity score” was calculated to represent the similarity between the genewise pattern of the TD results and the “ideal patterns” for each gene category. The “ideal patterns” were defined as vectors composed of 1, 0, and -1 for 16 gene categories (Virus_high, LPS_low, Virus_low, LPS_high, B_high, TNKM_low, B_low, TNKM_high, TNK_high, BM_low, TNK_low, BM_high, M_high, BTNK_low, M_low, and BTNK_high) (**Fig. S3D**). The “similarity score” was defined as the sum of the residual squares between the two vectors, the genewise vector of normalized values from the TD results (**Fig. S3C**) and the “ideal patterns” (**Fig. S3D**). According to the definition, the “similarity scores” for every combination of genes and gene categories were calculated. After calculating all similarity scores, to obtain the threshold for checking if a gene should be recognized as a gene in that category, the 20th percentile of the similarity score in the vector for each gene category was calculated.

In the third step (**Fig. S3F**), the gene category for each gene was determined. First, the candidate gene categories for each gene were filtered according to the pattern assigned in the first step (**Fig. S3C**). If the pattern (high/mid/low) of all 6 conditions was high or low, the gene was categorized as ALL_high or ALL_low, respectively. If the pattern of a gene matched the “ideal pattern” of a gene category, the gene category was added as a candidate gene category for the gene. For example,

if the pattern of gene A was (Virus_B: high, Virus_TNK: high, Virus_M: high, LPS_B: high, LPS_TNK: low, LPS_M: mid), the candidate gene category for gene A was "Virus_high" and "B_high" because all virus-infected data were assigned as "high" and all B-cell data were assigned as "high" (**Fig. S3D**). Second, the gene category with the lowest "similarity score" among the candidate gene categories was selected as the tentative gene category. In this selection, if the "similarity score" was higher than the threshold of the gene category (**Fig. S3E**), the gene was categorized as "Others" (See gene B in **Fig. S3F**) because the pattern for the gene was recognized as being too different from the "ideal pattern". If no candidate gene category was available, the gene was also classified as "Others" (See gene C in **Fig. S3F**). Finally, the final gene category was determined by integrating similar gene categories (**Fig. S3F**). For instance, the categories Virus_high and LPS_low were integrated into the category Virus_high because both categories indicated that virus-infected data were higher than LPS-stimulated data (See gene D in **Fig. S3F**). As a result of the gene classification process, genes were categorized into one of 11 categories (**Fig. 2C, S3D**).

GO term enrichment analysis

Gene Ontology (GO) analysis was performed with Fisher's exact test. This analysis used the GO canonical pathways and GO biological processes defined by MSigDB (RRID:SCR_022870) (v7.3) [30]. Adjusted P values were calculated using the Benjamini–Hochberg (BH) method.

Calculation of gene set variation analysis (GSVA) scores

The gene set-wise expression scores used in **Figs. 3A, 4B, 4C, and S4B** were calculated using GSVA (RRID:SCR_021058) (v1.38.2) [54, 55] with the algorithm "ssgsea".

Identification of differentially expressed genes (DEGs) and marker genes

In bat monocytes, DEGs were identified in Cluster 5 or Cluster 7 compared to the other clusters using the FindMarkers function of Seurat packages. A gene that met the following three criteria was considered a DEG: 1) the false discovery rate (FDR) calculated using the BH method was less than 0.05, 2) the average log2FC was greater than 1 or less than -1, and 3) the proportion of expressing cells was greater than 0.2.

The marker genes of Cluster 5 and Cluster 7 of bat monocytes (RaC5marker and RaC7marker, respectively) were defined as upregulated DEGs in Cluster 5 (**Fig. 4E**) and Cluster 7 (**Fig. 4G**), respectively.

Availability of source code and requirements

Project name: scRNA-seq_PBMC_Animals_Aso_et_al

Project homepage: https://github.com/TheSatoLab/scRNA-seq_PBMC_Animals_Aso_et_al [56]

Operating system: Linux

Programming languages: bash, R, Python

License: CC0-1.0

Data availability

The raw and processed single-cell RNA-seq data have been deposited in the Gene Expression Omnibus (GEO) database (GSE218199) and are publicly available. Original data to describe figures in this paper have been deposited at Mendeley [57] and are publicly available. All additional supporting data are available in the *GigaScience* database [59].

Declarations

List of abbreviations

cDCs: conventional dendritic cells; CIU: cell infectious unit; CP10k: counts per 10,000 counts in the cell; DEGs: differentially expressed genes; DMEM: Dulbecco's modified Eagle's medium; dsRNAs: double-stranded RNAs; FC: fold-change; FCS fetal calf serum; FDR: false discovery rate; GEMs: gel beads-in-emulsion; GEO: Gene Expression Omnibus; GSVA gene set variation analysis; GO: Gene Ontology; HOI: higher-order orthogonal iteration; Hs: *Homo sapiens*; HSV-1: herpes simplex virus type 1; IFNs: interferons; ISGs: IFN-stimulated genes; LPS: Lipopolysaccharide; mad: median absolute deviation; MAITs: mucosal-associated invariant T cells; Mm: *Macaca mulatta*; Monos: monocytes; NK: natural killer; PAMPs: pathogen-associated molecular patterns; PBMCs: peripheral blood mononuclear cells; PCs: principal components; PCA: principal component analysis; pDCs: plasmacytoid dendritic cells; PFU plaque forming unit; PRRs: pattern recognition receptors; Pt: *Pan troglodytes*; Ra: *Rousettus aegyptiacus*; RLRs: RIG-I-like receptors; scRNA-seq: single-cell RNA

sequencing; SeV: Sendai virus; TD: Tucker decomposition; UMAP: uniform manifold approximation and projection; UMI: unique molecular identifier; QC: quality control

Ethics statement

All protocols involving specimens from animals were performed in accordance with the Science Council of Japan’s Guidelines for the Proper Conduct of Animal Experiments. The protocols were approved by the Institutional Animal Care and Use Committee of Kyoto University (approval IDs: 2017-B-5, 2019-C-9, 2019-162, 2019-177, and 2020-C-5). All protocols involving specimens from humans recruited at Kyoto University were reviewed and approved by the Institutional Review Boards of Kyoto University (approval ID: G1089). All protocols for the use of human specimens were reviewed and approved by the Institutional Review Boards of The Institute of Medical Science, The University of Tokyo (approval ID: 2019-55) and Kyoto University (approval ID: G1089).

Consent for publication

All human subjects provided written informed consent.

Competing interests

The authors declare no competing interests.

Funding

This study was supported in part by AMED SCARDA Japan Initiative for World-leading Vaccine Research and Development Centers "UTOPIA" (JP223fa627001, to K.S.), AMED SCARDA Program on R&D of new generation vaccine including new modality application (JP223fa727002, to K.S.); AMED Research Program on Emerging and Re-emerging Infectious Diseases (JP22fk0108146, to Y.Kashima and K.S.; JP21fk0108494 to K.S.; 21fk0108425, to K.S.; 21fk0108432, to K.S.); AMED Research Program on HIV/AIDS (JP22fk0410039, to K.S.); JST PRESTO (JPMJPR22R1, to J.I.); AMED Moonshot Research and Development Program (JP21zf0127005, to H.O.); JST CREST (JPMJCR20H4, to K.S.); JSPS KAKENHI Grant-in-Aid for Early-Career Scientists (20K15767, to J.I.; 19K20394, to H.O.); JSPS Core-to-Core Program (A. Advanced Research Networks) (JPJSCCA20190008, to K.S.); JSPS Research Fellow DC1 (20J23299, to H.A.).

Author’s contributions

H.A. mainly performed bioinformatics analysis. J.I. and H.O. supervised the bioinformatics analysis. Y.Kashima mainly performed the experiments. Y.S., Y.Koyanagi, and K.S. supervised the experiments. K.S. and Y.Koyanagi provided reagents. K.S. conceived and designed the experiments. H.A. and J.I. wrote the initial manuscript. All authors reviewed and edited the manuscript.

Acknowledgements

We would like to thank Naoko Misawa, Akiko Oide, Mai Suganami, and Kazumi Abe (The University of Tokyo), for technical support, Hiroo Imai (Kyoto University) for providing primate PBMCs, Ayuko Morita (Kyoto City Institute of Health and Environmental Sciences) for providing bat PBMCs, Yasushi Kawaguchi (The University of Tokyo) for providing HSV-1, Takashi Irie (Hiroshima University) for providing SeV, and Human Genome Center (the Institute of Medical Science, the University of Tokyo) for providing the super-computing resource SHIROKANE (http://sc.hgc.jp/shirokane.html). This work was supported by the Cooperative Research Program of the Primate Research Institute, Kyoto University (2019-c9).

Author's information

H.A.'s current affiliation: Department of AI Systems Medicine, M&D Data Science Center, Tokyo Medical and Dental University, Tokyo 113-8510, Japan

Additional Files

Figure S1. Validation of viral infectivity and the innate immune response (related to Figure 1)

(A) Heatmap of the induction levels of genes related to the IFN response and inflammation. The rows indicate genes, and the columns indicate combinations of species, stimulus, and dose. The color represents the log2 Fold Change of ddCt upon stimulation measured by qRT–PCR. "rep. 1" and "rep. 2" indicate biological replicates. (B-C) Heatmap of the expression levels of viral genes (B: HSV-1; C: SeV) measured by qRT–PCR. The rows indicate viral genes, and the columns indicate combinations of species and doses. "rep. 1" and "rep. 2" indicate biological replicates. The color represents the ddCt values based on the expression levels of GAPDH.

(D-G) Violin plots of (D) the numbers of detected genes per cell before QC, (E) numbers of counted reads per cell before QC, (F) numbers of detected genes per cell after QC, and (G) numbers of counted reads per cell after QC.

Figure S2. Heterogeneous expression patterns in the four animal species (related to Figure 1)

(A-B) UMAP plots representing the gene expression patterns of PBMCs from the four species. Each dot is colored according to the results of unsupervised clustering (A) and reference-based label transfer (B).

(C) Heatmaps showing pairwise Euclid distances representing the gene expression differences among clusters. The distances were calculated using PCs 1-50 of the gene expression data.

(D-E) UMAP plots representing the gene expression patterns of PBMCs from the mock samples for the four species. Each dot is colored according to the results of unsupervised clustering using the integrated data for the four mock samples (D) or the four samples from each animal shown in **Figure S2A** (E).

(F) Heatmaps showing pairwise Euclid distances representing the gene expression differences among clusters shown in **Figure S2D**. The distances were calculated using PCs 1-30 of the gene expression data.

(G) Dot plots representing the expression patterns of marker genes for each cell type defined by Azimuth [58]

(H) Hierarchical clustering analysis of 48 pseudobulked FC gene expression datapoints (4 animal species x 4 stimuli x 11 cell types = 176 conditions).

Figure S3. Classification of genes according to species-specific expression patterns (related to Figure 2)

(A) Heatmap representing a latent factor matrix related to stimuli. The columns indicate stimuli, and the rows indicate latent factors representing stimulus-common (L2_1) and virus vs. LPS (L2_2) factors.

(B) Heatmap representing a latent factor matrix related to cell types. The columns indicate cell types, and the rows indicate latent factors representing cell type-common (L3_1), monocyte-specific (L3_2), and B-cell-specific (L1_3) factors.

(C) Summary of the normalization of values and patterning according to the ranking of the values. First, six column vectors (2 stimuli × 3 cell types) in the TD results were

normalized by dividing them by the 90th percentile of the individual vectors. Then, data with > 1 or < -1 were assigned as 1 and -1, respectively. Next, the genes were categorized into three classes (high, mid, and low) based on the rule that if the rank of a value was greater than the 80th percentile or smaller than the 20th percentile, it was categorized as “high” or “low”, respectively; otherwise, it was categorized as “mid”.

(D) Summary of the ideal patterns for each gene category used in the gene classification in **Figure 2C**.

(E) Summary of the calculation of the similarity score and establishment of the threshold for the gene classification in **Fig. S3F**. The sum of the residual squares between two vectors, the genewise vector of normalized values from the TD results (**Fig. S3C**) and the “ideal patterns” (**Fig. S3D**) were calculated. Then, the threshold used in **Fig. S3F** was obtained by calculating the 20th percentile of the similarity score for the vector for each gene category.

(F) Summary of gene classification. By comparing patterns from the TD results (**Fig. S3C**) and the ideal patterns (**Fig. S3D**), candidate gene categories were selected. Next, the gene category with the lowest “similarity score” among the candidate gene categories was selected as the tentative gene category. In this selection, if the “similarity score” was higher than the threshold of the gene category (**Fig. S3E**), the gene was categorized as “Others” (gene B). If no candidate gene category was available, the gene was also classified as “Others” (gene C). Finally, the final gene category was determined by integrating similar gene categories (genes A and D).

(G-I) Heatmap representing the values of the products calculated in **Figure 2C**. The data relating to (G) the species-common factor (L1_1), (H) the bat-specific factor (L1_2), and (I) the macaque-specific factor (L1_3) are shown. Each row indicates the respective gene. The color keys shown on the right of the heatmap indicate gene categories.

(J-L) Heatmap representing the FC values in the input tensor. The orders of the rows are the same as in (J) **Figure S3G**, (K) **Figure S3H**, and (L) **Figure S3I**. Each row indicates the respective gene. The color keys shown on the right of the heatmap indicate gene categories.

Figure S4. Identification of species-specific cell types (related to Figure 4)

(A) UMAP plots representing the expression patterns of every single cell. Dimensionality reduction was performed for each combination of the four species and three cell types.

(B) UMAP plots representing the average expression levels of marker genes for Cluster 7 [C7markers].

Table. S1. Primers used for RT–qPCR (related to the Methods)

The sequences of the primers used for RT–qPCR are listed.

References

1. Huff JL, Barry PA. B-virus (Cercopithecine herpesvirus 1) infection in humans and macaques: potential for zoonotic disease. *Emerg Infect Dis*. 2003;9(2):246-250.
2. Letko M, Seifert SN, Olival KJ, et al. Bat-borne virus diversity, spillover and emergence. *Nat Rev Microbiol*. 2020;18(8):461-471.
3. Towner JS, Amman BR, Sealy TK, et al. Isolation of genetically diverse Marburg viruses from Egyptian fruit bats. *PLoS Pathog*. 2009;5(7):e1000536.
4. Guito JC, Prescott JB, Arnold CE, et al. Asymptomatic infection of Marburg virus reservoir bats is explained by a strategy of immunoprotective disease tolerance. *Curr Biol*. 2021;31(2):257-270.e5.
5. Akira S, Uematsu S, Takeuchi O. Pathogen recognition and innate immunity. *Cell*. 2006;124(4):783-801.
6. Takeuchi O, Akira S. Pattern recognition receptors and inflammation. *Cell*. 2010;140(6):805-820.
7. Hansen JD, Vojtech LN, Laing KJ. Sensing disease and danger: a survey of vertebrate PRRs and their origins. *Dev Comp Immunol*. 2011;35(9):886-897.
8. Lu Y, Su F, Li Q, et al. Pattern recognition receptors in Drosophila immune responses. *Dev Comp Immunol*. 2020;102:103468.
9. Dempsey A, Bowie AG. Innate immune recognition of DNA: A recent history. *Virology*. 2015;479-480:146-152.
10. Poltorak A, He X, Smirnova I, et al. Defective LPS signaling in C3H/HeJ and C57BL/10ScCr mice: mutations in Tlr4 gene. *Science*. 1998;282:2085-2088.
11. Schountz T, Baker ML, Butler J, et al. Immunological control of viral infections in bats and the emergence of viruses highly pathogenic to humans. *Front Immunol*. 2017;8:1098.
12. Gorbunova V, Seluanov A, Kennedy BK. The world goes bats: Living longer and tolerating viruses. *Cell Metab*. 2020;32(1):31-43.
13. Banerjee A, Baker ML, Kulcsar K, et al. Novel insights into immune systems of bats. *Front Immunol*. 2020;11:26.
14. Pavlovich SS, Lovett SP, Koroleva G, et al. The Egyptian rousette genome reveals unexpected features of bat antiviral immunity. *Cell*. 2018;173(5):1098-1110.e18.

15. Zhou P, Tachedjian M, Wynne JW, et al. Contraction of the type I IFN locus and unusual constitutive expression of IFN-α in bats. *Proc Natl Acad Sci USA*. 2016;113(10):2696-2701.
16. Xie J, Li Y, Shen X, et al. Dampened STING-dependent interferon activation in bats. *Cell Host Microbe*. 2018;23(3):297-301.e4.
17. Ahn M, Cui J, Irving AT, et al. Unique loss of the PYHIN gene family in bats amongst mammals: Implications for inflammasome sensing. *Sci Rep*. 2016;6:21722.
18. Irving AT, Zhang Q, Kong PS, et al. Interferon regulatory factors IRF1 and IRF7 directly regulate gene expression in bats in response to viral infection. *Cell Rep*. 2020;33(5):108345.
19. De La Cruz-Rivera PC, Kanchwala M, Liang H, et al. The IFN response in bats displays distinctive IFN-stimulated gene expression kinetics with atypical RNASEL induction. *J Immunol*. 2018;200(1):209-217.
20. Lin HH, Horie M, Tomonaga K. A comprehensive profiling of innate immune responses in Eptesicus bat cells. *Microbiol Immunol*. 2022;66(3):97-112.
21. Ahn M, Anderson DE, Zhang Q, et al. Dampened NLRP3-mediated inflammation in bats and implications for a special viral reservoir host. *Nat Microbiol*. 2019;4(5):789-799.
22. Goh G, Ahn M, Zhu F, et al. Complementary regulation of caspase-1 and IL-1β reveals additional mechanisms of dampened inflammation in bats. *Proc Natl Acad Sci USA*. 2020;117(46):28939-28949.
23. Seurat (2021). Seurat (version 4.0.4). https://github.com/satijalab/seurat/releases/tag/v4.0.4.
24. Hao Y, Hao S, Andersen-Nissen E, et al. Integrated analysis of multimodal single-cell data. *Cell*. 2021;184(13):3573-3587.e29.
25. Azimuth (2020). Azimuth (version 0.4.3). https://github.com/satijalab/azimuth/releases/tag/v0.4.3.
26. Friedrichs V, Toussaint C, Schäfer A, et al. Landscape and age dynamics of immune cells in the Egyptian rousette bat. *Cell Rep*. 2022;40(10):111305.
27. Banerjee A, Rapin N, Bollinger T, et al. Lack of inflammatory gene expression in bats: a unique role for a transcription repressor. *Sci Rep*. 2017;7(1):2232.

28. Shaw AE, Hughes J, Gu Q, et al. Fundamental properties of the mammalian innate immune system revealed by multispecies comparison of type I interferon responses. *PLoS Biol*. 2017;15(12):e2004086.
29. Sato R, Kato A, Chimura T, et al. Combating herpesvirus encephalitis by potentiating a TLR3-mTORC2 axis. *Nat Immunol*. 2018;19(10):1071-1082.
30. Shah S, King EM, Chandrasekhar A, et al. Roles for the mitogen-activated protein kinase (MAPK) phosphatase, DUSP1, in feedback control of inflammatory gene expression and repression by dexamethasone. *J Biol Chem*. 2014;289(19):13667-13679.
31. Li QJ, Chau J, Ebert PJ, et al. miR-181a is an intrinsic modulator of T cell sensitivity and selection. *Cell*. 2007;129(1):147-161.
32. Posselt G, Schwarz H, Duschl A, et al. Suppressor of cytokine signaling 2 is a feedback inhibitor of TLR-induced activation in human monocyte-derived dendritic cells. *J Immunol*. 2011;187(6):2875-2884.
33. MSigDB (version 7.3). GSEA and MSigDB team, Broad Institute, Massachusetts. 2022. https://www.gsea-msigdb.org/gsea/msigdb/. Accessed 1 Sept 2022.
34. Seal S, Dharmarajan G, Khan I. Evolution of pathogen tolerance and emerging infections: A missing experimental paradigm. *Elife*. 2021;10:e68874.
35. Lafaille FG, Pessach IM, Zhang SY, et al. Impaired intrinsic immunity to HSV-1 in human iPSC-derived TLR3-deficient CNS cells. *Nature*. 2012;491:769-773.
36. López-Cotarelo P, Gómez-Moreira C, Criado-García O, et al. Beyond chemoattraction: Multifunctionality of chemokine receptors in leukocytes. *Trends Immunol*. 2017;38(12):927-941.
37. Abcam. Chemokines and their receptors. Abcam, UK. 2021. https://docs.abcam.com/pdf/immunology/chemokines_poster.pdf. Accessed 1 Sept 2022.
38. Ejercito PM, Kieff ED, Roizman B. Characterization of herpes simplex virus strains differing in their effects on social behaviour of infected cells. *J Gen Virol*. 1968;2(3):357-364.
39. Yoshida A, Kawabata R, Honda T, et al. A single amino acid substitution within the paramyxovirus Sendai virus nucleoprotein is a critical determinant for production of interferon-beta-inducing copyback-type defective interfering genomes. *J Virol*. 2018;92(5):e02094-17.

40. Kiyotani K, Sakaguchi T, Kato A, et al. Paramyxovirus Sendai virus V protein counteracts innate virus clearance through IRF-3 activation, but not via interferon, in mice. *Virology*. 2007;359(1):82-91.

41. Yamada E, Nakaoka S, Klein L, et al. Human-specific adaptations in Vpu conferring anti-tetherin activity are critical for efficient early HIV-1 replication in vivo. *Cell Host Microbe*. 2018;23(1):110-120.e7.

42. RefSeq. National Center for Biotechnology Information, Maryland. 2020. https://www.ncbi.nlm.nih.gov/refseq/. Accessed 6 Nov 2020.

43. 10X Genomics. Build a custom reference (cellranger mkref). 10X Genomics, California. 2020. https://support.10xgenomics.com/single-cell-gene-expression/software/pipelines/latest/using/tutorial_mr. Accessed 6 Nov 2020.

44. The report of orthologous genes. National Center for Biotechnology Information, Maryland. 2021. https://ftp.ncbi.nih.gov/gene/DATA/gene_orthologs.gz. Accessed 26 July 2021.

45. Jebb D, Huang Z, Pippel M, et al. Six reference-quality genomes reveal evolution of bat adaptations. *Nature*. 2020;583:578-584.

46. Bat1K Project. https://bat1k.com (2018). Accessed 14 July 2021.

47. UCSC genome browser. University of California, California. 2000. https://genome.ucsc.edu. Accessed 14 July 2021.

48. Quinlan AR, Hall IM. BEDTools: a flexible suite of utilities for comparing genomic features. *Bioinformatics*. 2010;26(6):841-842.

49. Cell Ranger (2021). Cell Ranger (version 6.0.1). https://support.10xgenomics.com/single-cell-gene-expression/software/downloads/6.0.

50. Zheng GXY, Terry JM, Belgrader P, et al. Massively parallel digital transcriptional profiling of single cells. *Nat Commun*. 2017;8:14049.

51. McInnes L, Healy J, Melville J. UMAP: Uniform manifold approximation and projection for dimension reduction. 2018. arXiv:1802.03426. https://doi.org/10.48550/arXiv.1802.03426.

52. TensorLy (2021). TensorLy (version 0.6.0). https://github.com/tensorly/tensorly/releases/tag/0.6.0.

53. rTensor (2022). rTensor (version 1.4.8). https://github.com/rikenbit/rTensor/releases/tag/v1.4.8.

54. GSVA (2021). GSVA (version 1.38.2). https://www.bioconductor.org/packages/release/bioc/html/GSVA.html.

55. Hänzelmann S, Castelo R, Guinney J. GSVA: gene set variation analysis for microarray and RNA-seq data. *BMC Bioinform*. 2013;14:7.

56. Aso H, Ito J, Ozaki H, et al. scRNA-seq_PBMC_Animals_Aso_et_al. *GitHub*. 2023. https://github.com/TheSatoLab/scRNA-seq_PBMC_Animals_Aso_et_al.

57. 57. Single-cell_transcriptome_analysis_illuminating_the_characteristics_of_species-specific_innate_immune_responses_against_viral_infections_Aso_et_al. Mendeley Data. 2023. DOI: 10.17632/kg3dfkyjv5.1

58. Azimuth references (Human - PBMC). The Human BioMolecular Atlas Program (HuBMAP), National Institutes of Health, Maryland. 2020. https://azimuth.hubmapconsortium.org/references/#Human%20-%20PBMC. Accessed 26 July 2021.

59. Aso H, Ito J, Ozaki H, et al. Supporting data for "Single-cell transcriptome analysis illuminating the characteristics of species-specific innate immune responses against viral infections" GigaScience Database. 2023. http://dx.doi.org/10.5524/102448.

Figure 1

Click here to access/download;Figure;Fig1.tif

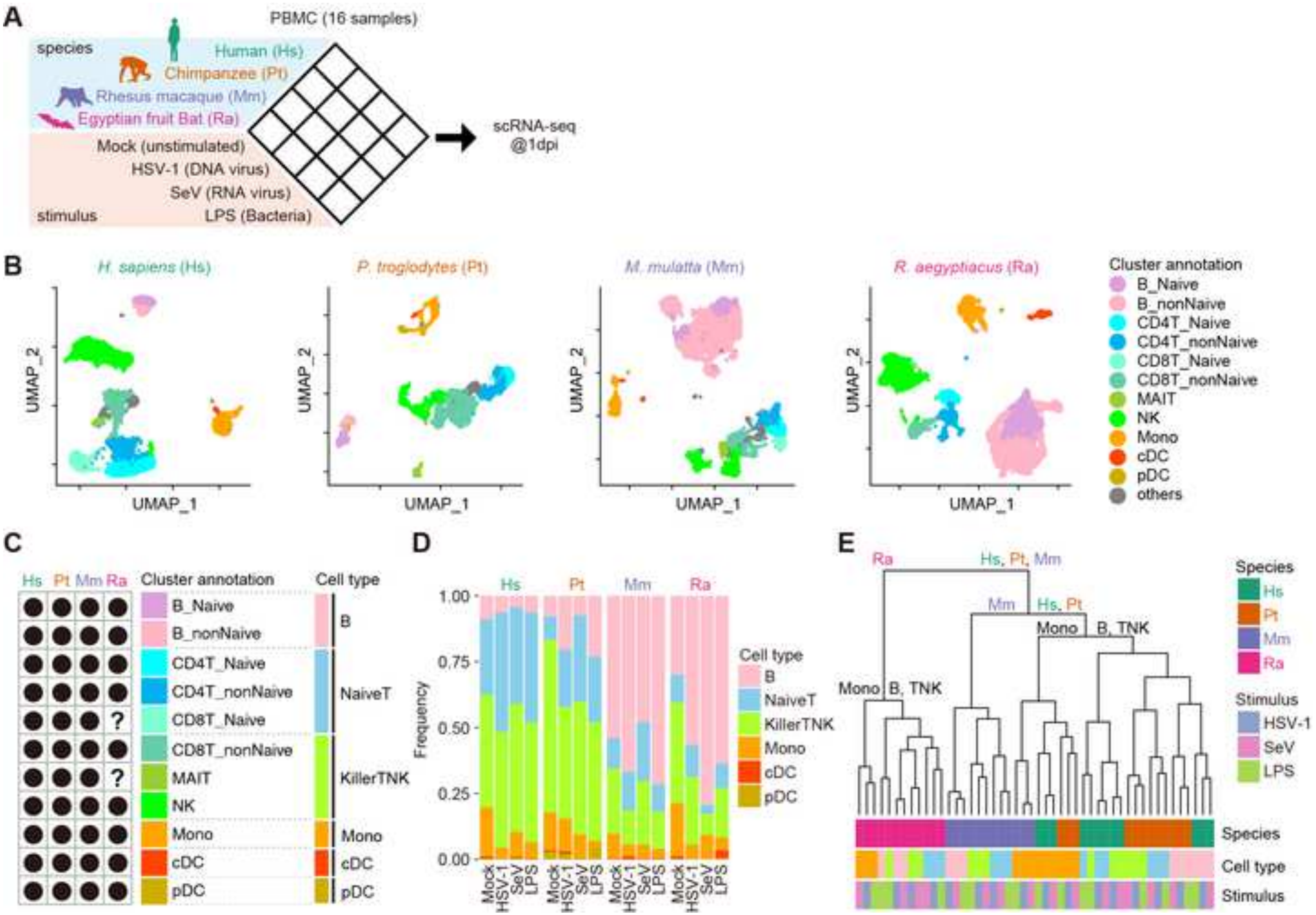

Figure 2 Click here to access/download;Figure;Fig2.tif

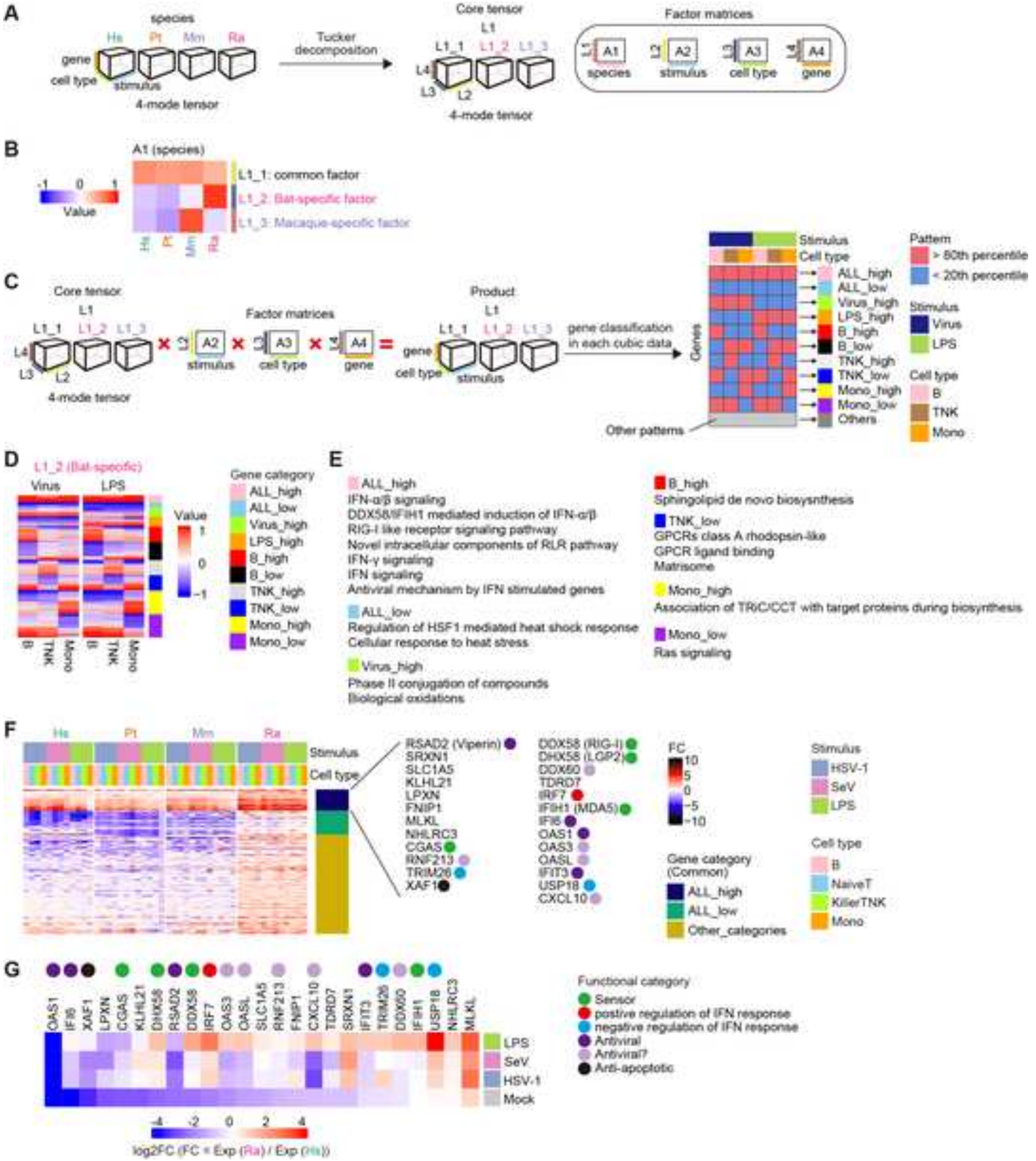

Figure 3

Click here to access/download;Figure;Fig3.tif

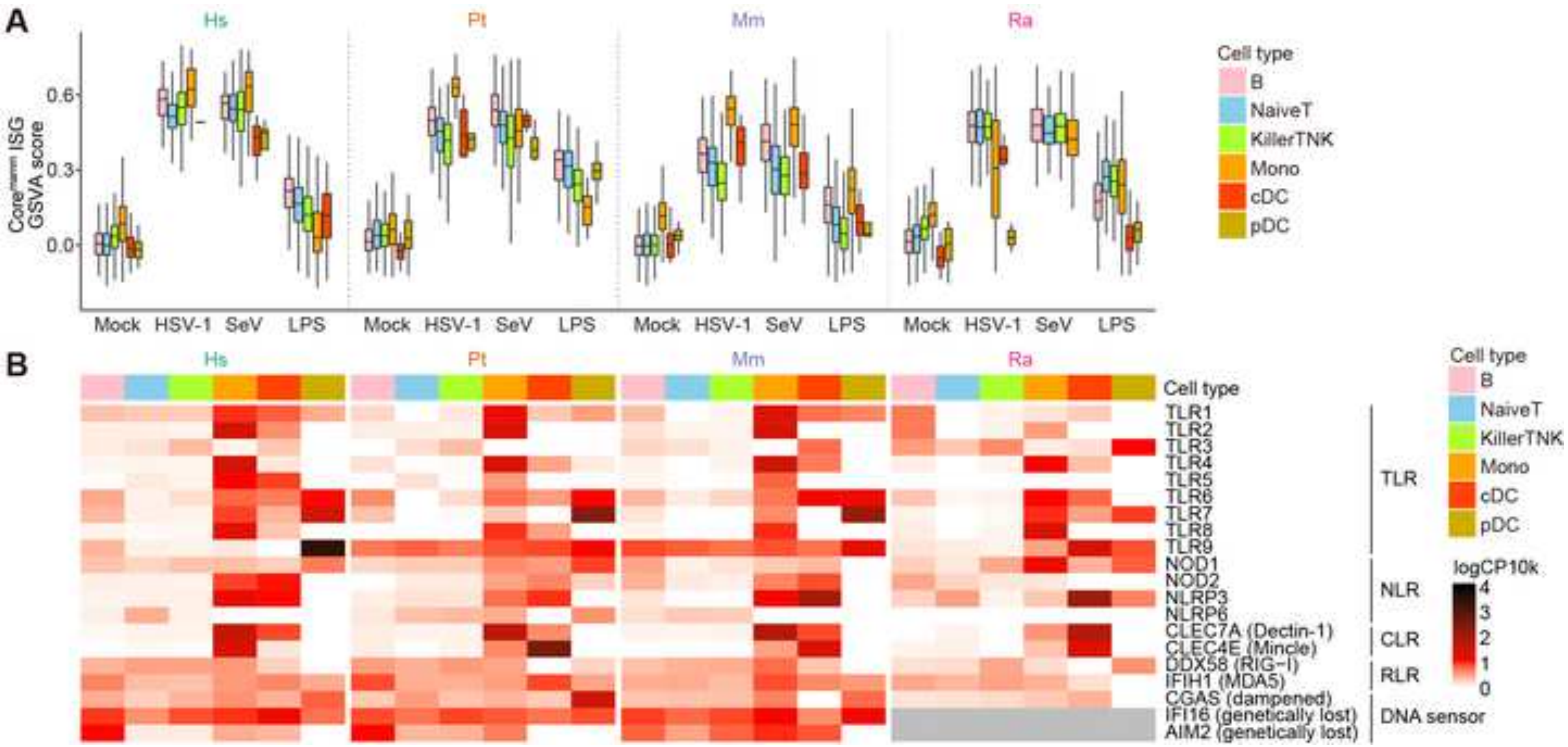

Figure 4

Click here to access/download;Figure;Fig4.tif

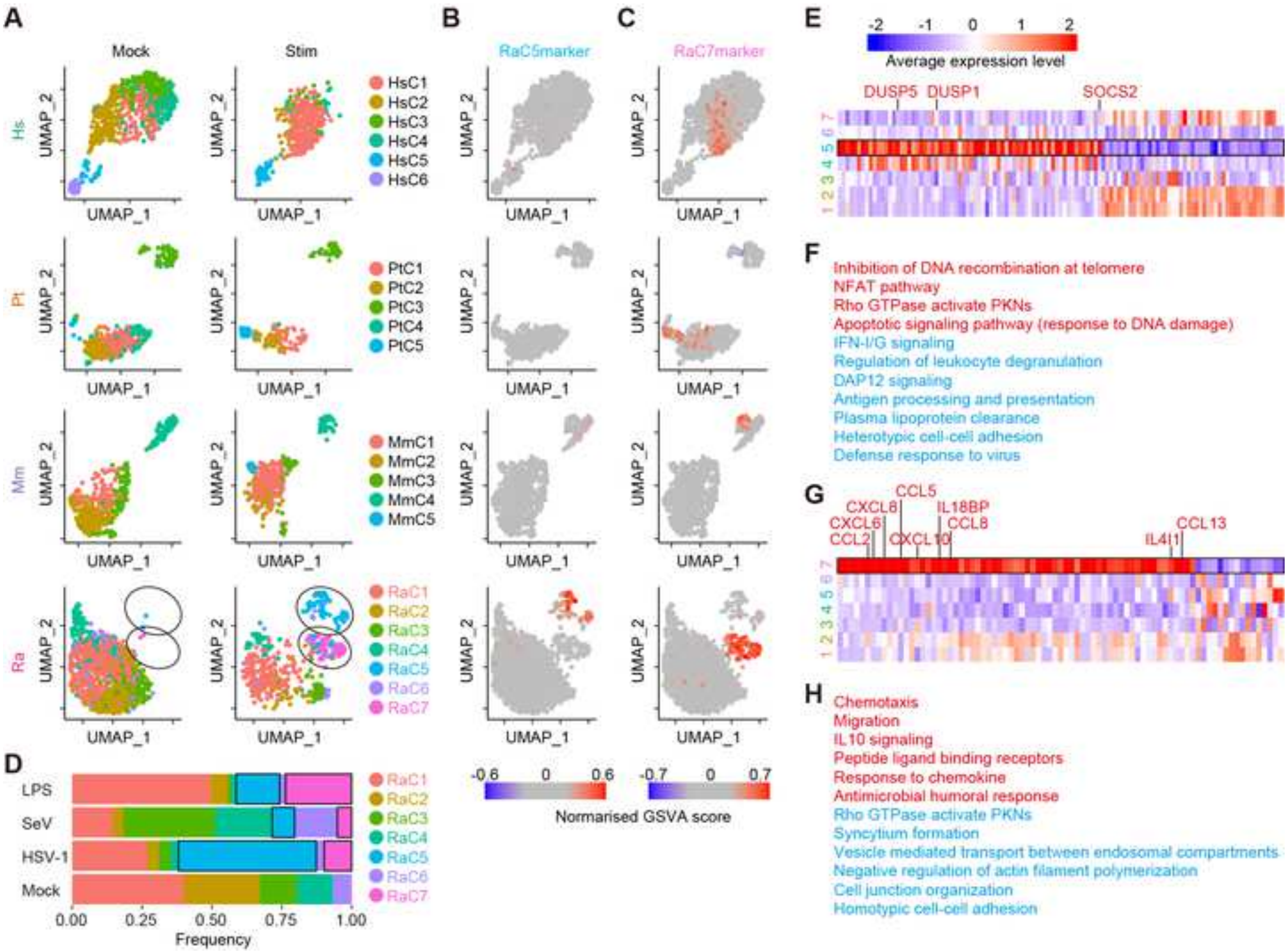

Reply to the reviewers

Click here to access/download

Supplementary Material

230812ResponseLetter_v12.docx

Figure S1

Click here to access/download
Supplementary Material
FigS1.tif

Figure S2

Click here to access/download
Supplementary Material
FigS2.tif

Figure S3

Click here to access/download
Supplementary Material
FigS3.tif

Figure S4

Click here to access/download

Supplementary Material

FigS4.tif

Table S1

Click here to access/download
Supplementary Material
TableS1.xlsx

Click here to access/download
Supplementary Material
230812draft_v32.docx

Personal Cover
Click here to access/download;Personal Cover;230813 CL_Aso.docx

August 13, 2023

Editor,
GigaScience

Please find enclosed our revised manuscript entitled "**Single-cell transcriptome analysis illuminating the characteristics of species-specific innate immune responses against viral infections**", by Aso et al., for the consideration of publication in ***GigaScience***.

According to the comments raised by the two referees, we modified the manuscript. We hope that the editor will find our study important, and consider it suitable for publication in ***GigaScience***.

Sincerely,

Kei
****
Kei Sato, Ph.D.
Professor, The Institute of Medical Science, The University of Tokyo, Japan.
Email: KeiSato@g.ecc.u-tokyo.ac.jp
****
