# Supplementary figures and images for: Single-cell transcriptome analysis illuminating the characteristics of species-specific innate immune responses against viral infections
Source: Gigascience. 2023 Oct 17;12:giad086. doi: 10.1093/gigascience/giad086 (PMC10580374; doi:10.1093/gigascience/giad086)

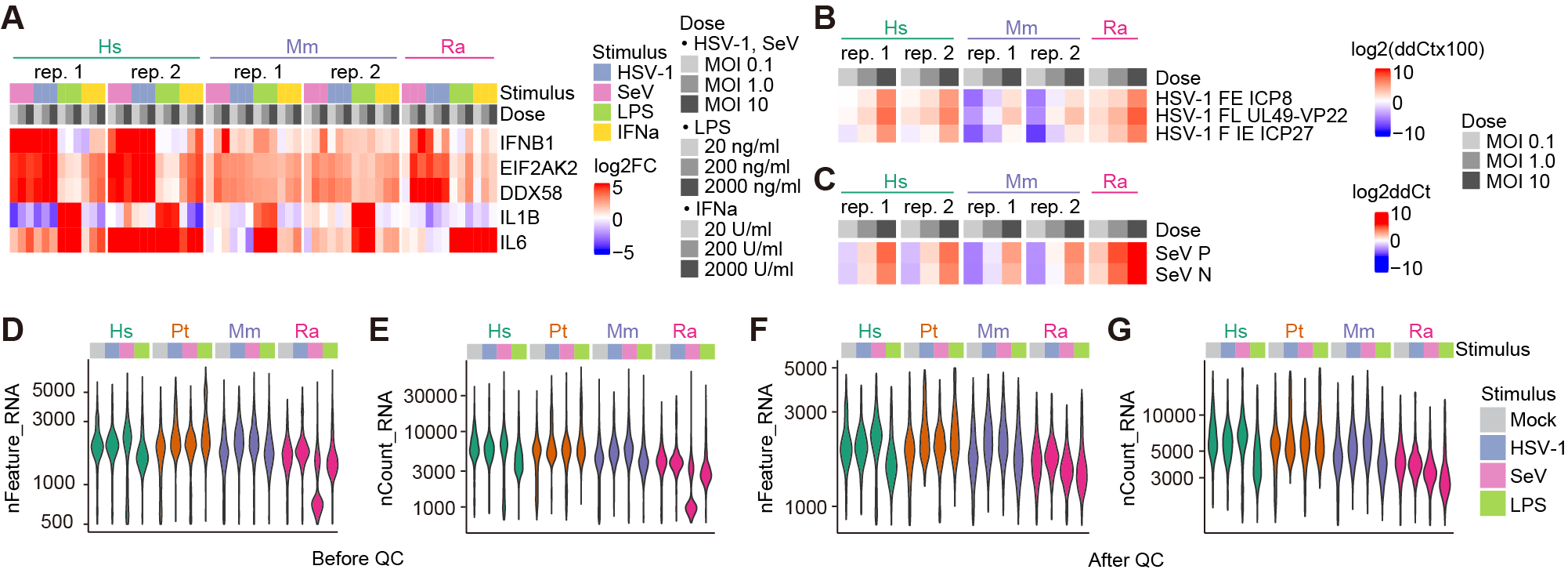

Supplement: giad086_Supplemental_Files [file giad086_supplemental_files.zip › FigS1.tif]

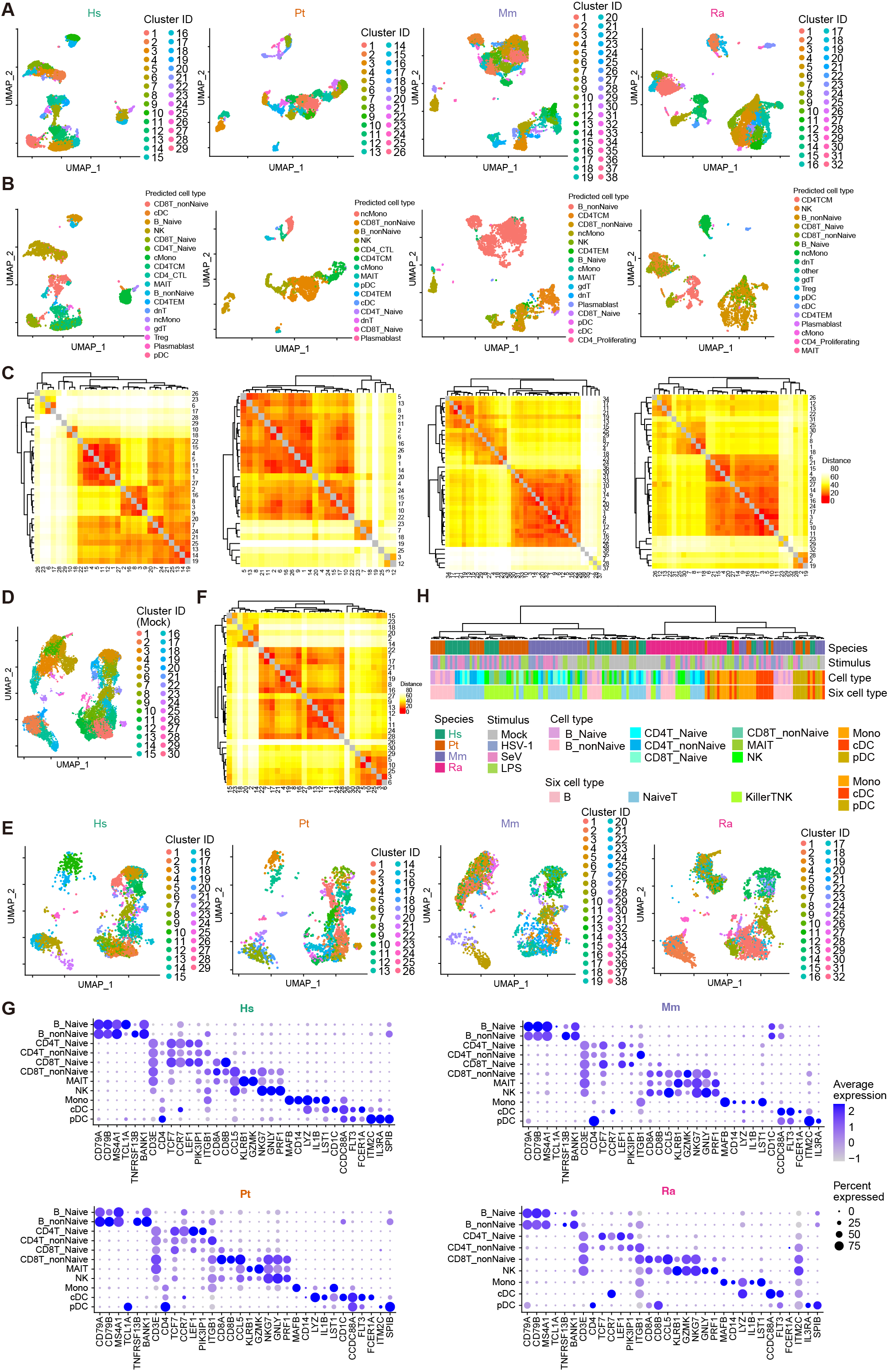

Supplement: giad086_Supplemental_Files [file giad086_supplemental_files.zip › FigS2.tif]

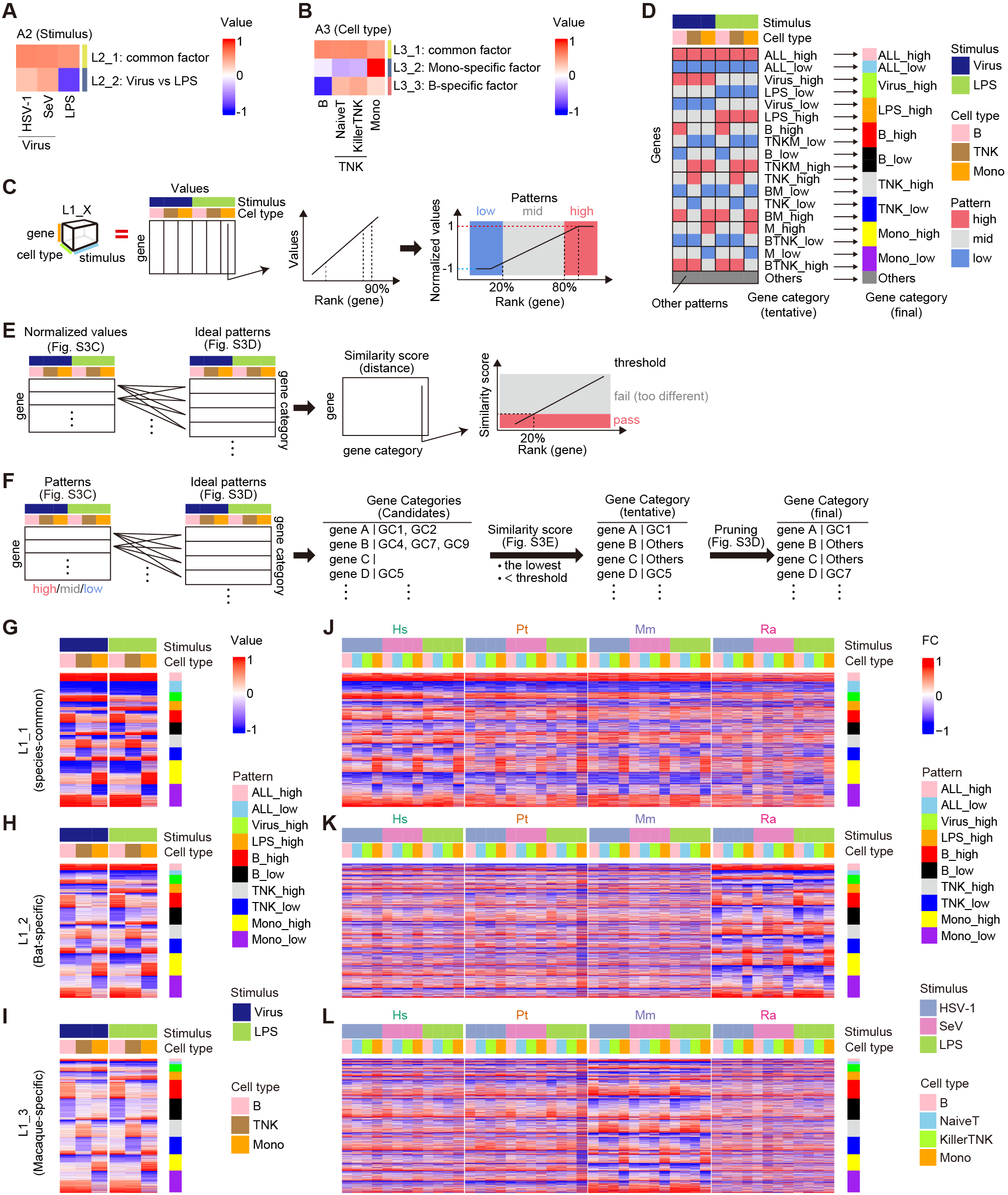

Supplement: giad086_Supplemental_Files [file giad086_supplemental_files.zip › FigS3.tif]

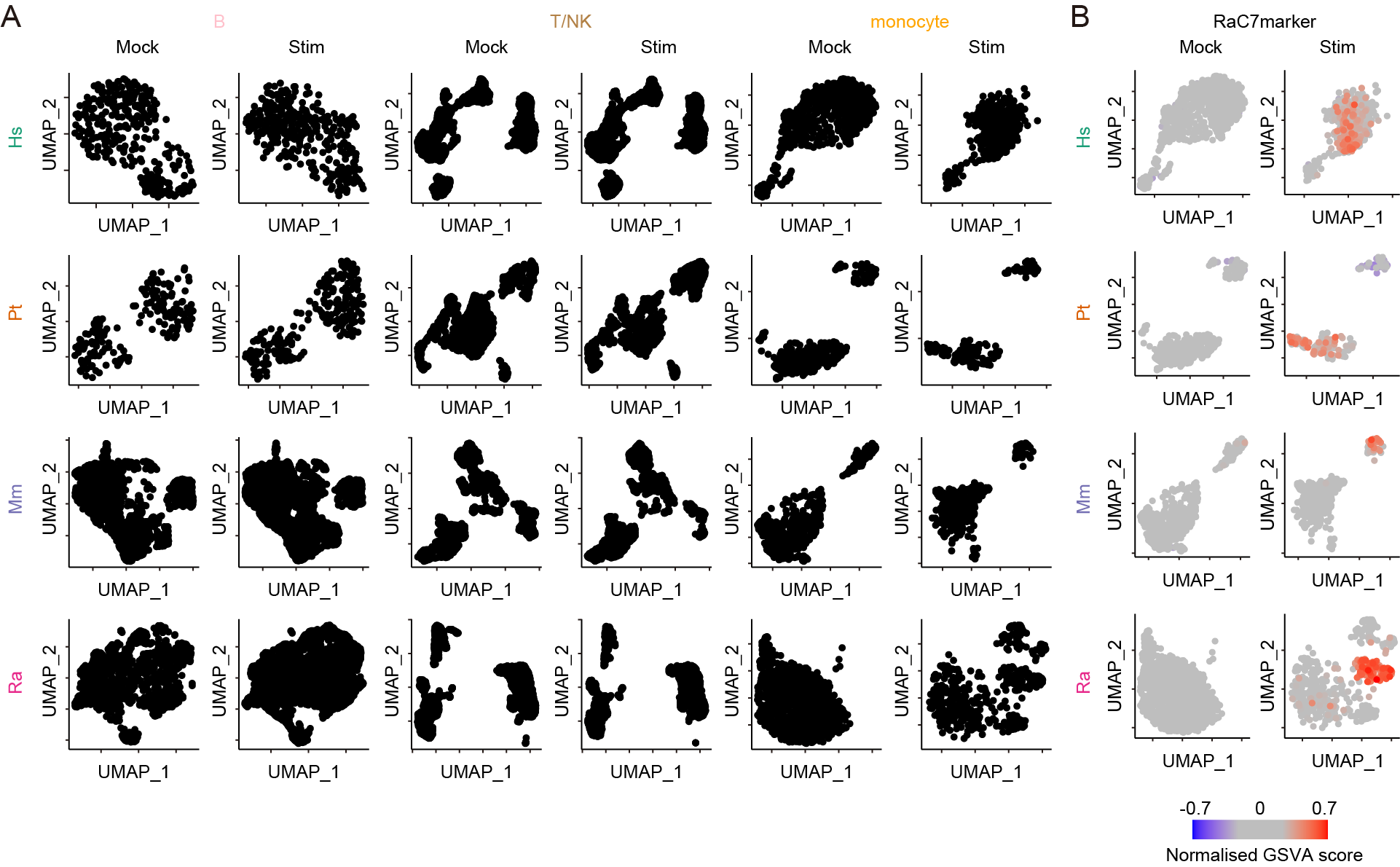

Supplement: giad086_Supplemental_Files [file giad086_supplemental_files.zip › FigS4.tif]
