# Supplementary material for: Single-cell transcriptome analysis illuminating the characteristics of species-specific innate immune responses against viral infections
Source: Gigascience. 2023 Oct 17;12:giad086. doi: 10.1093/gigascience/giad086 (PMC10580374; doi:10.1093/gigascience/giad086)
Supplement: giad086_Response_to_Reviewer_Comments_Original_Submission [file giad086_response_to_reviewer_comments_original_submission.pdf]

Author's Response To Reviewer Comments

*'Respond to Reviewers' is also prepared as a supplemental material (230812ResponseLetter_v12.docx). Please also find this file as well.

Reviewer #1: Hirofumi Aso and colleagues provide a manuscript entitled 'Single-cell transcriptome analysis illuminating the characteristics of species specific innate immune responses against viral infections'. The aim was to describe differences in innate immune responses of peripheral blood mononuclear cells (PBMCs) from different primates and bats against various pathogenic stimuli (different viruses and LPS). A major conclusion from the study is that differences in the immune response between primate and bat PBMCs are more pronounced than those between DNA, RNA viruses or LPS, or between the cell types.

The topic is of interest as the immunological basis for how bats appear to be largely disease resistant to some viruses that cause severe infections in humans is not well understood. One notion by others has been that bats have a larger spectrum of interferon (IFN) type I related genes, some of which are expressed constitutively even in unstimulated tissue, and there, trigger the expression of IFN stimulated genes (ISGs). Alongside, enhanced ISG levels may need to be compensated for in bats. Accordingly, bats may exhibit reduced diversity of DNA sensing pathways, as well as absence of a range of proinflammatory cytokines triggered in humans upon encountering acute disease causing viruses.

The study here uses single-cell RNA sequencing (scRNA-seq) analysis, and transcript clustering algorithms to explore the profile of different innate immune responses upon viral infections of PBMCs from H sapiens, Chimpanzee, Rhesus macaque, and Egyptian fruit bat. Most commonly referred to cell types were detected in all four species, although naïve CD8+ T cells were not detected in bat PBMCs, which led the authors to focus on B cells, naïve T cells, killer T/NK cells, monocytes, cDCs, and pDCs. The study used three pathogenic stimuli, Herpex simplex virus 1 (HSV1), Sendai virus (SeV), and lipopolysaccharide (LPS).

Specific comments

The text is well written, concise, and per se interesting, but I have a few questions for clarification.
Thank you very much for your high evaluation. We have responded to the points you raised and hope you recognize the improvements we have made.

1) Can the authors provide quality and purity control data for the virus inocula to document virus homogeneity? E.g., neither the methods, nor the indicated ref 26 specify if or how HSV1 was purified. Same is true for SeV where the provided ref 34 does not indicate if virus was purified or not. If virus inocula were not purified then it remains unclear to what extent the effects on the PBMCs described in the study here were due to virus or some other component in the inoculum. Conditions using inactivated inoculum might help to clarify this issue.
Thank you for pointing this out and we apologize for the lack of clarity in our descriptions regarding this point. In this study, we did not purify either HSV-1 or SeV. Thus, it is possible that something in the supernatant of virus-producing cells (including viruses) could affect the innate immune responses in PBMCs. However, given that the use of the supernatants without purification is a conventional virological method, any unanticipated effects would be much smaller than the effects of viral infection. To make it clear, we have added a description about this information in the revised manuscript in Materials and Methods (Lines 509-510, 522-523).

Lines 509-510: “Briefly, Vero cells were infected with HSV-1 and the supernatant was collected and used without purification.”

Lines 522-523: “Briefly, LLC-MK2 cells were infected with SeV and the supernatant was collected and used without purification.”

2) What was the infection period? Was it the same for all viruses?
Samples were analyzed at one day post infection. This is a condition shared by all viruses and stimuli, described in Lines 539-541 (Method: Infection and stimulation) in the revised manuscript. To emphasize that they are the same conditions, minor corrections have been made to the revised manuscript (Lines 129-133 and Lines 539-541).

Lines 129-133: "To analyze immune responses to stimuli at single-cell resolution, we performed scRNA-seq analysis of 16 types of PBMC samples: four mammalian species (Hs, Pt, Mm, and Ra) versus four conditions (mock infection/stimulation, HSV-1 infection, SeV infection, and LPS stimulation) using the 10x Genomics Chromium platform at one day post infection."

Lines 539-541: "At one day post infection, all types of infected/stimulated PBMCs were centrifuged, resuspended in PBS, and used for bulk RT–qPCR and scRNA-seq (see below)."

3) Upon stimuli application, there was a noteable expansion of B cells and a compression of killer T / NK cells in the bat but not the human samples, as well as compression of monocytes, the latter observed in all four species. Can the authors comment on this observation?
Thank you for your valuable suggestion. According to the reviewer's comment, we have edited the main text in the revised manuscript as follows (Lines 168-175, 326-328):

Lines 168-175: "The frequency of monocytes decreased after stimulation in all four species, whereas the frequency of B cells and killer TNK cells changed differently among the animal species and stimuli. Upon stimulation, there was generally a notable increase in B cells and a decrease of killer TNK cells in the bat (and non-human primates) samples, but not in the human samples. After SeV infection, the frequency of B cells was decreased in all four species. On the other hand, after HSV-1 infection, the frequency of B cells was decreased in only humans."

Lines 326-328: "It is also noteworthy that changes in cell composition differed between humans and bats (Fig. 1D)."

4) Lines 78-79: I do not think that TLR9 ought to be classified as a cytosolic DNA sensor. Please clarify.
Thank you for pointing this out. It is true that this wording was incorrect. Therefore, we fixed the issues in the manuscripts (Lines 80-82).

Lines 80-82: "Cytosolic Extrachromosomal DNAs, a PAMP for DNA viruses, are recognized by cytosolic DNA sensors, such as (e.g., cGAS, AIM2, and IFI16), and endosomal DNA sensors (e.g., TLR9)5,6,9 [5, 6, 9]."

5) Line 117: please clarify that the upregulation of proinflammatory cytokines, ISGs and IFNB1 was measured at the level of transcripts not protein.
Thank you for pointing this out. According to the reviewer's suggestion, we clarified that the upregulation of those factors was measured at the level of mRNA transcripts (Lines 127-128).

Lines 127-128: "IFNB1 (Fig. S1A–C) at the level of mRNA transcripts."

6) Line 244: DNA sensors. Authors report that bats responded well to DNA viruses, although some of their DNA sensing pathways (e.g., STING downstream of cGAS, AIM2 or IFI16) were attenuated compared to primates (H sapies, Chimpanzee, Macaque). And they elute to the dsRNA PRR TLR3. But I am not sure if TLR3 is the only PRR to compensate for attenuated DNA sensing pathways. The authors might want to explicitly discuss if other RNA sensors, such as RIG-I-like receptors (RIG-I, LGP2, MDA5) were upregulated similarly in bats as in primate cells upon inoculation with HSV1.
Thank you for the important suggestions. As you have pointed out, it is possible that PRRs other than TLR3 compensates for weak DNA sensing pathways for HSV-1. We specially mentioned TLR3 in the original manuscript because the importance of human TLR3 in the response to HSV-1 has been demonstrated (Sato, Kato et al., Nature Immunology, 2018). However, we agree with your opinion that other possibilities should also be mentioned and have added them in the revised manuscript (Lines 344-354).

Lines 344-354: "An alternative possibility is that the IFN response in response to HSV-1 infection was

triggered by sensing viral molecules other than DNAs: iIt is known that, in humans and mice, dsRNA sensing by TLR3 plays an important role in responding to HSV-1 infection26,31. Furthermore Additionally, the Egyptian fruit bat genome encodes an intact TLR3 gene (NCBI Gene ID: 107510436), and bat immune cells express TLR3 (Fig. 3B). Furthermore, other RNA sensors, such as RIG-I, LGP2, and MDA5, were upregulated in bat cells similarly as in primate cells upon HSV-1 infection (Fig. 3B). These data suggest that in bats, bat TLR3 or other RNA sensors in bats may compensate for the immune responses induced by weakened DNA sensing pathwaysors, leading to IFN responses to HSV-1 infection."

7) Is it known how much TLR3 protein is expressed in bat PBMCs under resting and stimulated conditions? Same question for the DNA and RNA sensor proteins, e.g., cGAS, AIM2 or IFI16, RIG-I, LGP2, MDA5, or effector proteins, such as STING.
To our knowledge, there are no publications quantifying the expression of TLR3 or other sensor genes in bats at the protein level. This is mainly due to the lack of antibodies recognizing these proteins of bats.

8) Can authors clarify if cGAS is part of the attenuated DNA sensors in the bat samples under study here? And it would be nice to see the attenuated response of DNA sensing pathways in the bat samples, as suspected from the literature, including STING downstream of cGAS, or AIM2 and IFI16.
Thank you for these suggestions. We address these two points as follows:

> Can authors clarify if cGAS is part of the attenuated DNA sensors in the bat samples under study here?
In a previous study (Xie et al., Cell Host & Microbe, 2018), the cGAS signaling pathway is reduced by a mutation in STING (not in cGAS), and this mutation is conserved in bat species including Rousettus aegyptiacus, the bat species analyzed in this study. Therefore, the cGAS signaling pathway should be reduced in the bat PBMC samples in this study. Thus, to clarify this point, we modified the text in the revised manuscript (Lines 335-337).

Lines 335-337: "It is known that two DNA sensing pathways mediated by cGAS-STING pathway [16]16 and PYHIN proteins, including AIM2 and IFI16 [17]17, are dampened in bats, including Egyptian fruit bats."

> And it would be nice to see the attenuated response of DNA sensing pathways in the bat samples, as suspected from the literature, including STING downstream of cGAS, or AIM2 and IFI16.
We agree this is a good idea. Unfortunately, it is difficult to evaluate the activity of each DNA sensing pathway using the limited number of bat samples in this study. Further studies are required.

9) What are the expression levels of IFN-I and related genes in the bat cells among the different stimuli?
Thank you very much for your constructive comments. We agree it is important to examine the expression level of IFN-I itself when discussing innate immune responses. Unfortunately, however, we could not obtain the expression level of IFN-α genes in bat samples because IFN-α genes were not included in the gene annotations of Rousettus aegyptiacus (both RefSeq and Bat1K). In addition, although we tried to measure the expression level of IFN-α genes in bat samples using the custom gene annotation including bat IFN-α genes, we failed to detect the expression of these genes (Figure R1A–F). Therefore, we did not focus on the expression of IFN-I genes in this study. Instead, we discussed the expression level of ISGs as a surrogate for the activity of IFN-I pathway as shown in Fig. 3A. We added this explanation in Results in the revised manuscript (Lines 261-268).

Lines 261-268: "To test this hypothesis, we analyzed the IFN response upon HSV-1 (a DNA virus) infection. However, the expression levels of IFN-α genes were not examined because they were not annotated in the transcript model for the Egyptian fruit bat used in this study, and the expression of IFN-β genes were too low. Thus, even though the expression levels of IFN-I is the primary factor to examine the activity of the IFN response, we instead by analyzing analyzed the induced levels of "coremamm ISGs"—a set of genes that are commonly induced by type I IFNs across mammals that were defined in a previous study 25 [28]."

Note: Although HSV-1-infected bat sample showed high expression levels of IFNA3 and IFNB1 (Figure R1E), only one cell expressed most of IFNA3 and IFNB1 (Figure R1F). Therefore, this cell was determined to be an outlier.

Figure R1. Difficulties in quantitative analysis of expression levels of IFN-ɑ in bat samples
(A) Schematic of the IFN-I loci near the KLHL9 locus, originated from Zhou et al. [PMID: 26903655]. (B) Information extracted from the transcript model (gtf file) of Rousettus aegyptiacus in the initial manuscript. (C) Results of blastn for IFN-ɑ sequences near the KLHL9 locus. Query: Rousettus aegyptiacus IFN-ɑ (GenBank: AB259762.1). Subject: mRouAeg1.p "NW_023416306.1:61702666-62433637". Red indicates the alignment patterns with high mapping scores. (D) Information extracted from custom gene annotation used in E and F. (E, F) Results of mapping and counting by CellRanger using custom gene annotation. (E) Sum of counts for each bat sample. (F) Distribution of counts per cell for IFNA3 (top) and IFNB1 (bottom) in HSV-1-infected bat sample.

10) Technical point: where can the raw scRNA-seq data be found?
As stated in Data and code availability, the raw scRNA-seq data is found in NCBI GEO (GSE218199). To clarify this, we modified the text in the revised manuscript (Lines 837-838).

Lines 837-838: "The raw and processed Ssingle-cell RNA-seq data have been deposited in the Gene Expression Omnibus (GEO) database (GSE218199) and are publicly available."

Reviewer #2: This paper gives a good introduction on bats as reservoirs of several viral infections, which studies have shown is due to the uniqueness of their immune system. They and others suggest that bats immune system is dampened exhibiting tolerance to various viruses. This gives the study a good rationale as to why study the bats immune system, compared to other mammals. They also give a good rationale as to why they used single-cell sequencing, to allow the identification of various cell types and the differences in these cell types. From their finding the main conclusions are that differences in the host species are more impactful; than those among the different stimuli. They also suggest that bats initiate an innate immune response after infection with DNA viruses through an alternative pathway. For example, the induction dynamics of PRRs seems to be different in their dataset. They also suggest this could be due to the presence of species-specific cellular subsets.

1. Interesting model system and a good comparison of bats with other mammals.
2. Good technique in using single-cell sequencing, with a clear rationale as to why it was chosen. This advances knowledge on what was already known about bats immune system, but the species-specific cellular subsets are new.
3. Interesting technique to go through the bulk transcriptomic data in four species and four conditions. This allowed findings of the most important genes/pathways.
4. Good rationale / flow of experiments from one to another
5. I liked that they investigated stimuli from different pathogens , including DNA, RNA virus and bacteria and still show that bats had a different immune system, in the different stimuli.
Thank you for your appreciation on various points. Based on your comments, we have made modifications to improve the manuscript.

Minor comments
1. Do they speculate this occurrence in is this just in Egyptian Fruit bats or all species of bats?
Although we consider that the differences between Rousettus aegyptiacus and primates observed in this study mainly originate from those between bats and primates, we agreed that there should be differences among bat species. To clarify this point, we added some descriptions to the Discussion (Limitations of the study) in the revised manuscript (Lines 407-410).

Lines 407-410: "Moreover, because the results of this study rely on an analysis using a single bat species, the Egyptian fruit bat, it is unclear whether the observed bat-specific characteristics are conserved across bat species."

2. Mentioned in the introduction why they used the egyptian fruit bats - which are a model organism, but this could help people who are not in this field understand exactly why use these bats. Advantages? Location? Proximity to the various viruses based on the fact they are mostly found in endemic regions such as Africa etc.
Thank you for mentioning this important point. Indeed, the description of the reason for using the Egyptian Fruit bats was insufficient. The first reason why we selected this bat species is that they were available because they were captive bred. The second reason is that they are a species known to be asymptomatically infected with human pathogenic viruses, such as Marburg virus. We have stated this point in the Introduction and Results in the revised manuscript (Lines 107-111 and Lines 119-122).

Lines 107-111: “Here, we used peripheral blood mononuclear cells (PBMCs) from four mammalian species including the Egyptian fruit bats and three pathogenic stimuli and conducted single-cell RNA sequencing (scRNA-seq) analysis to elucidate the differences in innate immune responses against pathogenic stimuli.”

Lines 119-122: “In this study, the Egyptian fruit bat was used as a representative model organism for bat species because this bat species is bred and available in captivity and is known as natural host of human pathogenic viruses, such as Marburg virus [3].”

3. Can they include viral load in each species?
"Viral load" was not quantified, but the amount of RNA in the collected samples was quantified by RT-qPCR (Fig. S1B–C).

4. It is not clear which scRNAseq tools were used for data analysis in identifying the types of cells. Or did they use already established database based on markers?
Thank you for this comment. Details of the method can be found in the Cell Annotation section of Methods. In short, this study uses Seurat and Azimuth to annotate each cluster resulting from unsupervised clustering based on several pieces of information (Fig. S2A–F). In Azimuth, we also use reference data created based on markers (https://azimuth.hubmapconsortium.org). However, since it was written only in Methods not written in Results, we have now added a description in Results in the revised manuscript (Lines 143-145).

Lines 143-145: “We characterized the cellular composition of PBMCs from each mammalian species by annotating the cell type of individual single cells using tools available in Seurat [23, 24] and Azimuth [25] (See Methods).”

Anonymous Reviewer: I find after reading the MS that under my normal reviewer conditions I would have no choice but to reject the manuscript.

The structure and design of the MS is ok normally however the actual data has problems in that there is only n=1 sample. For each species (n=4) and each condition (n=4) there are indeed n=16 lots of single cell data. This however means there isn't even a single repeat. There is so much variation within bats, let alone between treatments or between species that simply n=1 for any scientific experiment is not acceptable. Further there are 40,717 cells detected between 16 separate experiments leaving a very low number of cells.

If you look at the raw data for fig S1D, you can see the cell number for bats is incredibly low.
Rousettus_aegyptiacus Mock B 1549
Rousettus_aegyptiacus Mock NaiveT 541
Rousettus_aegyptiacus Mock KillerTNK 1960
Rousettus_aegyptiacus Mock Mono 1035
Rousettus_aegyptiacus Mock cDC 57
Rousettus_aegyptiacus Mock pDC 10
Rousettus_aegyptiacus HSV1 B 767
Rousettus_aegyptiacus HSV1 NaiveT 166
Rousettus_aegyptiacus HSV1 KillerTNK 347
Rousettus_aegyptiacus HSV1 Mono 71

Rousettus_aegyptiacus HSV1 cDC 3
Rousettus_aegyptiacus HSV1 pDC 2
Rousettus_aegyptiacus SeV B 1160
Rousettus_aegyptiacus SeV NaiveT 48
Rousettus_aegyptiacus SeV KillerTNK 116
Rousettus_aegyptiacus SeV Mono 137

Gigascience prides itself on high-quality datasets but this set is way too minimal. I feel the data quality is well below GigaScience's level. Its a real pity as the manuscript is otherwise written and presented well - with a logical flow and decent structure for analysing the data.
Regarding biological replicates:
We agree with the importance of biological replicates, but it is cost-prohibitive to perform scRNA-seq for 16 samples with replicates. For clarity, we have now added the fact that we did not have biological replicates in the Limitations of the study section (Line 410).

Line 410: "Furthermore, we did not perform biological replicates of scRNA-seq in this study."

Regarding your point about low cell numbers:
The average cell numbers were about 2,500 cells/run after quality control (QC). Technically, it is possible to obtain higher cell numbers (e.g. more than 10,000 cells/run) before QC, but the higher the value, the higher the percentage of cell multiplets. That's why in this study, we targeted a concentration preparation of 5,000 cells/run before QC. Furthermore, since this study used stimulation by viral infection and cells from animals other than humans, it was expected that the value would be lower than the theoretical value. Considering these factors, we believe that the average of 2500 cells/run after QC, which is 1/2 of the target value before QC, is sufficiently high. Based on these considerations and your feedback, we have now added descriptions of conditions (cell concentration and number of target cells) to Methods in the revised manuscript (Lines 557-558).

Lines 557-558: "Before loading, cell numbers and viability were confirmed. To acquire 5,000 cells recovery, 8,000 cells were loaded."

There are problems with the methods aswell when the authors made certain decisions without any explanation or discussion (or reference for similar) e.g. "Cells with 800–5,000 genes/cell or 1,200–25,000 counts/cell were extracted." "Second, we removed nontargeted cells in the present study." "Finally, regarding genes/cell and counts/cell values, cells with >3 |Z score| were excluded.". This could then bias the data (and indeed seems to whereby the majority of lower-count cells for SeV are removed before and after QC - this obviously suggests Sev-infection reduces gene transcription in cells but these cells are filtered out.
Thank you for pointing this out. The cell filtering pipeline and its threshold have been set to ensure valid comparisons. As you mentioned, there was a group of cells with significantly low genes/cell and counts/cell values in Bat SeV. One possible interpretation of this could be that SeV infection may suppress the gene expression of these cells. However, it is reasonable to exclude these cells in which lower number of genes are detected from the analysis because it would be problematic to perform the analysis if these cells are included in the downstream analysis. Thus, we added the explanation why these cells were excluded in Results (Lines 133-139) and Methods (Lines 637-649) in the revised manuscript. Next, the cell types excluded from the analysis are erythrocytes, platelets, hematopoietic stem cells, and innate lymphoid cells. For erythrocytes and platelets, we removed them because these cell types are not members of PBMCs. Hematopoietic stem cells and innate lymphoid cells were excluded because they are not major cell types when discussing innate immune responses in PBMCs. We have also now added an explanation about this in Methods in the revised manuscript (Lines 637-649).

Lines 133-139: "Next, quality control (QC) was performed to exclude both cells with lower data quality and cells not targeted in this study (Fig. S1D–G) (See Methods). Before QC, there was a group of cells with low genes-per-cell and counts-per-cell in PBMCs of SeV-infected bats (Fig. S1D–E). Although one possible interpretation of this could be that SeV infection may suppressed the gene expression in these cells, these cells were excluded to ensure the integrity of the downstream quantitative analysis."

Lines 637-649: “The thresholds were determined based on the distributions of genes/cell and counts/cell before QC (Fig. S1D–E). Second, we removed nontargeted cells in the present study. We annotated the cell type of individual cells using Azimuth (v0.4.3) [25], a reference-based cell annotation prediction program (https://azimuth.hubmapconsortium.org), and then, cells annotated as erythrocytes, platelets, hematopoietic stem cells, or innate lymphoid cells, and platelets were excluded as nontargeted cells in the present study. This is because erythrocytes and platelets are probably residuals after experimental PBMC extraction, and hematopoietic stem cells and innate lymphoid cells are not the major cell types in the analysis of innate immune responses using PBMCs. In this step, the gene annotation “genes shared with humans” (see Gene annotation and ortholog information) for each animal species was used. Finally, regarding genes/cell and counts/cell values, cells with >3 |Z score| were excluded as outliers.”

The very limited dataset then also has different methods for PBMC extraction applied to different species (particularly whereby the anaesthesia used in chimpanzee is known to affect profile of PBMCs). Yet these cells are all dumped together for comparisons of human only or bat only genes.
As you pointed out, the method of obtaining PBMCs differs from animal to animal. However, this is in accordance with the characteristics of the animals, so it is unavoidable from an ethical point of view. Also, the main analysis is about the differences between bats and mammals and does not mention chimpanzee-specific characteristics. Furthermore, the results that chimpanzee cells showed similar expression patterns to those of humans and different from those of macaques and bats are consistent with the fact that chimpanzees are evolutionally close to humans. The results also support the idea that anesthesia did not have a significant effect. In conclusion, we believe that the method of PBMC acquisition in this study was appropriate.

There are other minor problems whereby the count normalization (post-transformation) is merged for all 4 species but after that differen settings (by species) a used in FindClusters (the idea of normalization should overcome this and allow similar settings to be used).
Because the hyperparameters of FindClusters are particularly sensitive to cell number, we used the hyperparameters that were adjusted for the cell number in each animal in this study.

They also perform statistics via FIshers exact test (despite n=1) for GO analysis in a rather unusual and creative way for GO of X against all others (rather than mock vs treatment) due to the lack of replicates.
In this study, we used an overlap-based (over-representation-based) GO enrichment analysis, which tests whether the list of genes with a specific GO term are significantly overlapped with the list of genes of interest using Fisher’s exact test. In our knowledge, this is a widely used method of GO enrichment analysis as described in Zhao and Rhee et al. (https://doi.org/10.1016/j.tig.2023.01.003). In Fig. 2E, GO term enrichment analysis was performed to check which GO terms are enriched in each gene list (ALL_high, Virus_high, TNK_low, etc.). In Fig. 4F and 4H, GO term enrichment analysis was performed on the list of DEGs in RaC5 and RaC7. Given the nature of GO term enrichment analysis, the issue of biological replicates is irrelevant because the number of genes (not the number of samples) is considered by Fisher’s exact test in this context.
