# Supplementary material for: Single-cell transcriptome analysis illuminating the characteristics of species-specific innate immune responses against viral infections
Source: Gigascience. 2023 Oct 17;12:giad086. doi: 10.1093/gigascience/giad086 (PMC10580374; doi:10.1093/gigascience/giad086)
Supplement: giad086_Reviewer_1_Report_Original_Submission — Urs Greber -- 3/14/2023 Reviewed [file giad086_reviewer_1_report_original_submission.pdf]

**Reviewer Report**

**Title: Single-cell transcriptome analysis illuminating the characteristics of species-specific innate immune responses against viral infections**

**Version: Original Submission** **Date:** 3/14/2023

**Reviewer name: Urs Greber**

**Reviewer Comments to Author:**

Hirofumi Aso and colleagues provide a manuscript entitled 'Single-cell transcriptome analysis illuminating the characteristics of species specific innate immune responses against viral infections'. The aim was to describe differences in innate immune responses of peripheral blood mononuclear cells (PBMCs) from different primates and bats against various pathogenic stimuli (different viruses and LPS). A major conclusion from the study is that differences in the immune response between primate and bat PBMCs are more pronounced than those between DNA, RNA viruses or LPS, or between the cell types. The topic is of interest as the immunological basis for how bats appear to be largely disease resistant to some viruses that cause severe infections in humans is not well understood. One notion by others has been that bats have a larger spectrum of interferon (IFN) type I related genes, some of which are expressed constitutively even in unstimulated tissue, and there, trigger the expression of IFN stimulated genes (ISGs). Alongside, enhanced ISG levels may need to be compensated for in bats. Accordingly, bats may exhibit reduced diversity of DNA sensing pathways, as well as absence of a range of proinflammatory cytokines triggered in humans upon encountering acute disease causing viruses.
The study here uses single-cell RNA sequencing (scRNA-seq) analysis, and transcript clustering algorithms to explore the profile of different innate immune responses upon viral infections of PBMCs from H sapiens, Chimpanzee, Rhesus macaque, and Egyptian fruit bat. Most commonly referred to cell types were detected in all four species, although naÃ¯ve CD8+ T cells were not detected in bat PBMCs, which led the authors to focus on B cells, naÃ¯ve T cells, killer T/NK cells, monocytes, cDCs, and pDCs. The study used three pathogenic stimuli, Herpex simplex virus 1 (HSV1), Sendai virus (SeV), and lipopolysaccharide (LPS).
Specific comments
The text is well written, concise, and per se interesting, but I have a few questions for clarification.
1) Can the authors provide quality and purity control data for the virus inocula to document virus homogeneity? E.g., neither the methods, nor the indicated ref 26 specify if or how HSV1 was purified. Same is true for SeV where the provided ref 34 does not indicate if virus was purified or not. If virus inocula were not purified then it remains unclear to what extent the effects on the PBMCs described in the study here were due to virus or some other component in the inoculum. Conditions using inactivated inoculum might help to clarify this issue.
2) What was the infection period? Was it the same for all viruses?
3) Upon stimuli application, there was a noteable expansion of B cells and a compression of killer T / NK cells in the bat but not the human samples, as well as compression of monocytes, the latter observed in all four species. Can the authors comment on this observation?

4) Lines 78-79: I do not think that TLR9 ought to be classified as a cytosolic DNA sensor. Please clarify.
5) Line 117: please clarify that the upregulation of proinflammatory cytokines, ISGs and IFNB1 was measured at the level of transcripts not protein.
6) Line 244: DNA sensors. Authors report that bats responded well to DNA viruses, although some of their DNA sensing pathways (e.g., STING downstream of cGAS, AIM2 or IFI16) were attenuated compared to primates (H sapies, Chimpanzee, Macaque). And they elute to the dsRNA PRR TLR3. But I am not sure if TLR3 is the only PRR to compensate for attenuated DNA sensing pathways. The authors might want to explicitly discuss if other RNA sensors, such as RIG-I-like receptors (RIG-I, LGP2, MDA5) were upregulated similarly in bats as in primate cells upon inoculation with HSV1.
7) Is it known how much TLR3 protein is expressed in bat PBMCs under resting and stimulated conditions? Same question for the DNA and RNA sensor proteins, e.g., cGAS, AIM2 or IFI16, RIG-I, LGP2, MDA5, or effector proteins, such as STING.
8) Can authors clarify if cGAS is part of the attenuated DNA sensors in the bat samples under study here? And it would be nice to see the attenuated response of DNA sensing pathways in the bat samples, as suspected from the literature, including STING downstream of cGAS, or AIM2 and IFI16.
9) What are the expression levels of IFN-I and related genes in the bat cells among the different stimuli?
10) Technical point: where can the raw scRNA-seq data be found?

**Methods**

Are the methods appropriate to the aims of the study, are they well described, and are necessary controls included? Choose an item.

**Conclusions**

Are the conclusions adequately supported by the data shown? Choose an item.

**Reporting Standards**

Does the manuscript adhere to the journal's guidelines on minimum standards of reporting? Choose an item.

Choose an item.

**Statistics**

Are you able to assess all statistics in the manuscript, including the appropriateness of statistical tests used? Choose an item.

**Quality of Written English**

Please indicate the quality of language in the manuscript: Choose an item.

**Declaration of Competing Interests**

Please complete a declaration of competing interests, considering the following questions:

- Have you in the past five years received reimbursements, fees, funding, or salary from an organisation that may in any way gain or lose financially from the publication of this manuscript, either now or in the future?
- Do you hold any stocks or shares in an organisation that may in any way gain or lose financially from the publication of this manuscript, either now or in the future?
- Do you hold or are you currently applying for any patents relating to the content of the manuscript?
- Have you received reimbursements, fees, funding, or salary from an organization that holds or has applied for patents relating to the content of the manuscript?
- Do you have any other financial competing interests?
- Do you have any non-financial competing interests in relation to this paper?

If you can answer no to all of the above, write 'I declare that I have no competing interests' below. If your reply is yes to any, please give details below.

I declare that I have no competing interests

I agree to the open peer review policy of the journal. I understand that my name will be included on my report to the authors and, if the manuscript is accepted for publication, my named report including any attachments I upload will be posted on the website along with the authors' responses. I agree for my report to be made available under an Open Access Creative Commons CC-BY license (http://creativecommons.org/licenses/by/4.0/). I understand that any comments which I do not wish to be included in my named report can be included as confidential comments to the editors, which will not be published.

Choose an item.

To further support our reviewers, we have joined with Publons, where you can gain additional credit to further highlight your hard work (see: https://publons.com/journal/530/gigascience). On publication of this paper, your review will be automatically added to Publons, you can then choose whether or not to claim your Publons credit. I understand this statement.

Yes Choose an item.
