# Supplementary material for: Single-cell transcriptome analysis illuminating the characteristics of species-specific innate immune responses against viral infections
Source: Gigascience. 2023 Oct 17;12:giad086. doi: 10.1093/gigascience/giad086 (PMC10580374; doi:10.1093/gigascience/giad086)
Supplement: giad086_Reviewer_2_Report_Original_Submission — Doreen Ikhuva Lugano -- 5/23/2023 Reviewed [file giad086_reviewer_2_report_original_submission.pdf]

Reviewer Report

Title: Single-cell transcriptome analysis illuminating the characteristics of species-specific innate immune responses against viral infections

Version: Original Submission **Date:** 5/23/2023

Reviewer name: Doreen Ikhuva Lugano

Reviewer Comments to Author:

This paper gives a good introduction on bats as reservoirs of several viral infections, which studies have shown is due to the uniqueness of their immune system. They and others suggest that bats immune system is dampened exhibiting tolerance to various viruses. This gives the study a good rationale as to why study the bats immune system, compared to other mammals. They also give a good rationale as to why they used single-cell sequencing, to allow the identification of various cell types and the differences in these cell types. From their finding the main conclusions are that differences in the host species are more impactful; than those among the different stimuli. They also suggest that bats initiate an innate immune response after infection with DNA viruses through an alternative pathway. For example, the induction dynamics of PRRs seems to be different in their dataset. They also suggest this could be due to the presence of species-specific cellular subsets.
1. Interesting model system and a good comparison of bats with other mammals.
2. Good technique in using single-cell sequencing, with a clear rationale as to why it was chosen. This advances knowledge on what was already known about bats immune system, but the species-specific cellular subsets are new.
3. Interesting technique to go through the bulk transcriptomic data in four species and four conditions. This allowed findings of the most important genes/pathways.
4. Good rationale / flow of experiments from one to another
5. I liked that they investigated stimuli from different pathogens , including DNA, RNA virus and bacteria and still show that bats had a different immune system, in the different stimuli.
Minor comments
1. Do they speculate this occurrence in is this just in Egyptian Fruit bats or all species of bats?
2. Mentioned in the introduction why they used the egyptian fruit bats - which are a model organism, but this could help people who are not in this field understand exactly why use these bats. Advantages? Location? Proximity to the various viruses based on the fact they are mostly found in endemic regions such as Africa etc.
3. Can they include viral load in each species?
4. It is not clear which scRNAseq tools were used for data analysis in identifying the types of cells. Or did they use already established database based on markers?

Methods

Are the methods appropriate to the aims of the study, are they well described, and are necessary controls included? Choose an item.

Conclusions

Are the conclusions adequately supported by the data shown? Choose an item.

Reporting Standards

Does the manuscript adhere to the journal's guidelines on minimum standards of reporting? Choose an item.

Choose an item.

Statistics

Are you able to assess all statistics in the manuscript, including the appropriateness of statistical tests used? Choose an item.

Quality of Written English

Please indicate the quality of language in the manuscript: Choose an item.

Declaration of Competing Interests

Please complete a declaration of competing interests, considering the following questions:

- Have you in the past five years received reimbursements, fees, funding, or salary from an organisation that may in any way gain or lose financially from the publication of this manuscript, either now or in the future?
- Do you hold any stocks or shares in an organisation that may in any way gain or lose financially from the publication of this manuscript, either now or in the future?
- Do you hold or are you currently applying for any patents relating to the content of the manuscript?
- Have you received reimbursements, fees, funding, or salary from an organization that holds or has applied for patents relating to the content of the manuscript?
- Do you have any other financial competing interests?
- Do you have any non-financial competing interests in relation to this paper?

If you can answer no to all of the above, write 'I declare that I have no competing interests' below. If your reply is yes to any, please give details below.

I declare that I have no competing interests

I agree to the open peer review policy of the journal. I understand that my name will be included on my report to the authors and, if the manuscript is accepted for publication, my named report including any attachments I upload will be posted on the website along with the authors' responses. I agree for my

report to be made available under an Open Access Creative Commons CC-BY license (http://creativecommons.org/licenses/by/4.0/). I understand that any comments which I do not wish to be included in my named report can be included as confidential comments to the editors, which will not be published.

Choose an item.

To further support our reviewers, we have joined with Publons, where you can gain additional credit to further highlight your hard work (see: https://publons.com/journal/530/gigascience). On publication of this paper, your review will be automatically added to Publons, you can then choose whether or not to claim your Publons credit. I understand this statement.

Yes Choose an item.
